# Functional Characterization of *Pseudoidium neolycopersici* Photolyase Reveals Mechanisms Behind the Efficacy of Nighttime UV on Powdery Mildew Suppression

**DOI:** 10.3389/fmicb.2020.01091

**Published:** 2020-05-29

**Authors:** Ranjana Pathak, Åshild Ergon, Arne Stensvand, Hans Ragnar Gislerød, Knut Asbjørn Solhaug, Lance Cadle-Davidson, Aruppillai Suthaparan

**Affiliations:** ^1^Department of Plant Sciences, Faculty of Biosciences, Norwegian University of Life Sciences, Ås, Norway; ^2^Division of Biotechnology and Plant Health, Norwegian Institute of Bioeconomy Research, Ås, Norway; ^3^Faculty of Environmental Sciences and Natural Resource Management, Norwegian University of Life Sciences, Ås, Norway; ^4^Grape Genetics Research Unit, Agricultural Research Service, United States Department of Agriculture, Geneva, NY, United States

**Keywords:** absorption spectra, action spectra, cryptochrome, CPD photolyase, DNA damage

## Abstract

Powdery mildews can be controlled by brief exposure to ultraviolet (UV) radiation with devastating effect on their developmental stages including conidia germination. The treatment effect can be impaired by subsequent exposure to UV-A/blue light. UV-A/blue light-activated photolyase may be responsible for this and therefore we tested the function of three cryptochrome/photolyase family (CPF)-like genes (OINE01015670_T110144, OINE01000912_T103440, and OINE01005061_T102555) identified in the obligate biotrophic fungus *Pseudoidium neolycopersici*, the cause of tomato powdery mildew. A photolyase-deficient mutant of *Escherichia coli* transformed with coding sequence of OINE01000912_T103440 and exposed to brief (UV)-C treatment (peak emission at 254 nm) showed photoreactivation and cell survival when exposed to subsequent blue light, indicating complementation of photolyase activity. In contrast, the same photolyase-deficient *E. coli* transformed with the coding sequences of other two CPF-like genes did not survive this treatment, even though their expression were confirmed at protein level. This confirmed that OINE01000912_T103440 is a gene encoding photolyase, here named *PnPHR1*, with functionality similar to the native photolyase in *E. coli*, and classified as a class I cyclobutane pyrimidine dimer (CPD) photolyase. Modeling of the 634-amino acid sequence of *PnPHR1* suggested that it is capable of binding flavin adenine dinucleotide (FAD) and methenyltetrahydrofolate (MTHF). However, spectroscopic data of the protein produced in an *E. coli* expression system could only reveal the presence of a reduced form of FAD, i.e., FADH^–^ as an intrinsic chromophore. Within the tested wavelength range of 365–525 nm, the survival of photolyase-deficient mutant *E. coli* transformed with *PnPHR1 s*howed a broad action spectrum from 365 to 454 nm. This was very similar to the previously characterized action spectrum for survival of *P. neolycopersici* conidia that had been treated with UV-C. Quantitative RT-PCR revealed that the expression of *PnPHR1* in *P. neolycopersici* conidia was induced by UV-C, and peak expression occurred 4 h after brief UV-C treatment. The expression of *PnPHR1* was repressed when incubated in red light after the UV-C treatment, but not when incubated in UV-A/blue light. The results may explain why the disease-reducing effect of short wavelength UV is impaired by exposure to UV-A and blue light.

## Introduction

Powdery mildews are caused by obligate biotrophic fungal pathogens in the order Erysiphales. Nearly 10,000 plant species can be infected by these fungi (Glawe, 2008; Braun and Cook, 2012), and the disease can be devastating if not properly managed. Tomato powdery mildew, caused by *Pseudoidium neolycopersici* (formerly known as *Oidium neolycopersici*), is of great economic importance worldwide in greenhouse and field-grown tomatoes (Jankovics et al., 2008; Li et al., 2012). The pathogen typically thrives in protected cultivation environments where there is poor ventilation, high humidity and moderate temperatures, and it is difficult to eradicate once established. Several control measures, including fungicide treatments and breeding programs to develop resistant cultivars, are being employed for better disease management (Li et al., 2012).

Extensive research has examined the potential of different spectral qualities, irradiance levels and duration of irradiance in control of powdery mildews for a wide range of crops (Suthaparan et al., 2010, 2012a, 2016a; Van Delm et al., 2014; Janisiewicz et al., 2016). Short wavelength (<290 nm) ultraviolet radiation (UV) and red light have shown great potential against powdery mildews (Suthaparan et al., 2012a, 2014, 2016b, 2018). However, the efficacy of UV against powdery mildew varies depending on the spectral quality of the subsequent growth light. Brief exposure to UV during dark or in combination with red light showed higher disease control efficacy than exposure to UV in the presence of light with wavelengths shorter than 500 nm. Furthermore, using growth lights with an increased proportion of wavelengths below 500 nm immediately following the UV treatment decreased the efficacy of the nighttime UV treatment against powdery mildew (Suthaparan et al., 2017, 2018). This confirmed that recovery of germination in *P. neolycopersici* conidia treated with brief UV is dependent on the wavelength, and indicates the presence of possible light-mediated repair mechanisms of UV-induced damage in the powdery mildews (Suthaparan et al., 2018).

Ultraviolet has deleterious effects on all life forms, ranging from bacteria to higher plants, animals, and humans. DNA is the cellular component which is most significantly affected by UV (Weber, 2005). The predominant UV-induced DNA damage is the formation of pyrimidine dimers (Sancar, 1994). UV induces two major types of lesions in DNA, cyclobutane pyrimidine dimers (CPDs) and (6–4) photoproducts, which constitute 80–90% and 10–20% of the damage, respectively (Sinha and Häder, 2002; Sancar, 2008). If the damage is not repaired, it can result in an arrested cell cycle due to blocking of replication and transcription (Sancar, 1994). Different DNA damage repair mechanisms include nucleotide excision repair, recombination repair, mutagenic repair, and photolyase-mediated repair. Out of these, photolyase-mediated repair (also known as photoreactivation, or photorepair) is the simplest and most rapid mechanism because of the involvement of a single enzyme (Sinha and Häder, 2002). It is also the only repair mechanism regulated by light (Essen and Klar, 2006). Photolyases absorb energy in the near UV to blue regions (300–500 nm) and use this energy to catalyze the repair of CPDs and (6–4) photoproducts (Sinha and Häder, 2002; Thompson and Sancar, 2002). All photolyases contain a common catalytic cofactor, flavin adenine dinucleotide (FAD), and an additional second cofactor, which is methenyltetrahydrofolate (MTHF) in the majority of species, and 8-hydroxy-7, 8-didemethyl-5-deazariboflavin (8-HDF) in a limited number of species. Depending on their substrate binding specificity, photolyases have been categorized into CPD photolyases and (6–4) photolyases (Weber, 2005; Essen and Klar, 2006).

Photolyases have been structurally and functionally characterized in many life forms, including filamentous fungi and plants (Sancar et al., 1987b; Yajima et al., 1991; Waterworth et al., 2002). However, there are no reports on the functional characteristics of photolyases in obligate biotrophic fungi. Using next generation transcriptome sequencing of *Erysiphe necator*, the cause of grape powdery mildew, the presence and expression of putatively photo-responsive genes similar to phytochromes, cryptochromes, white collar, and photolyases have been identified (Suthaparan et al., 2012b). Preliminary analysis of whole genome and transcriptome sequencing of *P. neolycopersici* as well as *Podosphaera xanthii* and *Podosphaera aphanis*, the causal agents of powdery mildew in cucumber and strawberry, respectively, confirmed the presence of genes similar to all major classes of photoreceptors, including photolyase, cryptochrome, white collar, phototropin, phytochrome, and UVR8 (Pathak et al., 2017). Three genes similar to the blue light-absorbing cryptochrome/photolyase family (CPF)-like genes were identified in each of these three species, but the function of the gene products has not been characterized (Pathak et al., 2017). Despite a high degree of sequence homology, photolyases and cryptochromes perform two distinct functions, photolyase repair DNA damage caused by UV while cryptochromes play a key role in circadian entrainment in animals and regulate growth and development in plants (Thompson and Sancar, 2002; Zhang et al., 2017).

The objectives of this study were to identify the functional photolyase gene among three putative CPF-like genes in *P. neolycopersici*, to characterize its action and absorption spectra, and to test for gene induction in response to UV.

## Materials and Methods

### Phylogenetic Analysis of CPF-Like Genes

Amino acid sequences of 137 CPF-like genes (Supplementary Data S2) were retrieved from National Centre for Biotechnology Information^1^. These were, together with predicted amino acid sequences of the three CPF-like genes from *P. neolycopersici* (OINE01015670_T110144, OINE01000912_T103440, and OINE01005061_T102555) subjected to phylogenetic analysis using the MEGAX software package (Kumar et al., 2018). Multiple sequence alignment was done by ClustalW with default parameters and an unrooted tree of sequence data was constructed by implementing neighbor-joining algorithm with 1000 bootstrap replicates.

### Cloning of Putative CPF-Like Genes

The full-length coding regions of three putative CPF-like genes (OINE01015670_T110144, OINE01000912_T103440, and OINE01005061_T102555) identified in the *P. neolycopersici* genome (Suthaparan A. et al., unpublished) were PCR-amplified from cDNA (Supplementary Table S1), cloned into a pCR^TM^ 2.1-TOPO^®^ TA cloning vector (Thermo Fisher Scientific, United States) and sequenced. The sequences (NCBI GeneBank accession numbers MT277362, MT277363, and MT277364) were adapted to the codon usage in *E. coli* using OptimumGene^TM^ and synthesized (GenScript, United States). The synthetic genes PN5670, PN0912, and PN5061, with *Sph*I and *Xma*I restriction sites, were excised and ligated into the Sphl/Xmal sites of the pQE-30Xa (AmpR) expression vector with 6× His-tag at the N-terminus (Qiagen, Germany). The resulting three constructs were named pQE-30Xa_PN5670, pQE-30Xa_PN0912, and pQE-30Xa_PN5061 and maintained in *E. coli* DH5α in 15% glycerol at −80°C, for the following survival assays.

### Survival Assay With Wild Type and Photolyase-Deficient *E. coli* Strains Transformed With Putative CPF-Like Genes

Two *E. coli* strains were used to test the photoreactivation activity of the three putative CPF-like genes: KY1056 (*recA56, phr^+^*), which is photolyase-proficient, and KY1225 (*recA56, phr*^–^), which is photolyase-deficient. The two *E. coli* host strains were first transformed with the pREP4 (Kan^R^) repressor plasmid (Qiagen, Germany), carrying a lac repressor that tightly regulates the lac promoter-controlled expression of recombinant proteins in the pQE-30Xa (Amp^R^) vector. The pREP4 (Kan^R^) transformed strains were subsequently transformed with pQE-30Xa with and without gene inserts, resulting in the following transgenic strains: (i) KY1056_pQE-30Xa (without gene insert), (ii) KY1225_pQE-30Xa (without gene insert), (iii) KY1225_pQE-30Xa_PN5670, (iv) KY1225_pQE-30Xa_PN0912, and (v) KY1225_pQE-30Xa_PN5061. KY1056_pQE-30Xa and KY1225_pQE-30Xa were used as positive and negative controls, respectively. Transformed cultures were maintained on Lysogeny Broth (LB) agar (15% w/v) supplemented with 100 μg/ml ampicillin and 25 μg/ml kanamycin as selective antibiotics.

For all UV-C treatments described in this study, 120 cm fluorescent tubes with UV emission peak at 254 nm (120 W germicidal UV-C tubes; Light Tech, United States) were used. A preliminary experiment confirmed that exposure to UV-C (254 nm, 2 ± 0.2 μmol/m^2^/s) for 10 s was sufficient to have a significant effect on survival of *E. coli* strains KY1056 and KY1225 (Supplementary Figure S1). For a qualitative survival assay, transgenic *E. coli* strains were grown to saturation at 28°C overnight. Fresh LB medium supplemented with selective antibiotics was inoculated using saturated colonies with the dilution of 1:100, and further grown at 28°C until they reached an optical density of 0.5 at 600 nm wavelength (OD_600_ = 0.5). To induce gene expression, isopropyl-1-thio-D-galactopyranoside (IPTG) was added to a final concentration of 20 μg/ml in the cultures, and the cultures were allowed to continue growing for an additional 4 h. Then cultures were diluted to OD_600_ = 0.05 and plated on LB agar only supplemented with required antibiotics. No IPTG was used in LB agar plates at any stage of the experiment. Immediately after plating, Petri dishes without lids were exposed to either (i) UV-C treatment (254 nm) of 2 ± 0.2 μmol m^–2^ s^–1^ for 10 s or (ii) complete darkness. Petri dishes were sealed immediately after UV-C exposure and incubated at 25°C with either, (i) 2 h of blue light with an irradiance of 25 ± 5 μmol m^–2^ s^–1^ (peak at 454 nm) (15 W GreenPower LED module HF blue; Philips, Netherlands) or (ii) 2 h of complete darkness. All samples were subsequently incubated in darkness at 37°C overnight. On the next day, plates were assessed for surviving colonies. At this stage of experiment, growing cultures of all three constructs (pQE-30Xa_PN5670, pQE-30Xa_PN0912, and pQE-30Xa_PN5061) were tested for protein expression by western blot (Supplementary Figure S2). It was shown that all three constructs were expressing under similar conditions used in survival assay but only pQE-30Xa_PN0912 has photolyase like activity.

Based on the results of the above qualitative survival assay, the *E*. *coli* strain with a functional photolyase gene (KY1225_pQE-30Xa_PN0912) was selected for the quantitative assay. KY1056_pQE-30Xa and KY1225_pQE-30Xa (both without gene inserts) were again used as positive and negative controls, respectively. The experiment was performed as described above, except that the plated bacteria were first incubated at 37°C overnight in dark without any light treatment. On the next day, single colonies were picked and transferred to new Petri dishes with LB agar containing selective antibiotics (50 colonies per Petri dish). Plates were then exposed to the different treatments as described above. On the following day, the number of surviving colonies were recorded. Four replicate plates per treatment were used, and the experiment was performed twice.

### Structure Model of Putative Photolyase of *P. neolycopersici*

The amino acid sequence of the *P. neolycopersici* photolyase gene (OINE01000912_T103440) identified in the *E. coli* survival assay was used in modeling of its three-dimensional (3D) structure. A 3D structure model was generated using the protein structure and function prediction server RaptorX (Källberg et al., 2012). RaptorX can take the advantage of the sequence conservation in known photolyase proteins and generated a reliable homology model of the conserved region of the protein, based on experimentally determined structures of photolyases in Protein data bank (PDB^2^). To predict the presence of the type of second cofactor (MTHF or 8-HDF), the generated 3D structure model of *P. neolycopersici* was visualized and superimposed with photolyases from *E. coli* containing 5, 10-MTHF (PDB id: 1DNP) or *Anacystis nidulans* (PDB id: 1QNF) containing 8-HDF, using the PyMOL software (PyMOL Molecular Graphics System, Version 2.0 Schrödinger, LLC). An amino acid sequence alignment of the putative *P. neolycopersici* photolyase and the *E. coli* and *A. nidulans* photolyases was made by Clustal Omega (Sievers et al., 2011).

### Expression and Purification of a Putative *P. neolycopersici* Photolyase

Expression and purification of the putative *P. neolycopersici* photolyase (PN0912) was done by GenScript (United States) as follows. pQE30-Xa_PN0912 does not yield sufficient protein for purification. Hence, the linear pET-30a (Kan^R^) expression plasmid (Novagen, Germany) containing gene PN0912 with 6× His-tag at C- terminus, was used for overexpression using *E. coli* BL21 (DE3) cells. A single colony was used to inoculate 4 ml of LB containing kanamycin and incubated overnight at 37°C and 200 rpm. Four milliliter of an overnight-grown culture was used to inoculate 1 L of auto-induced medium of 5052 containing selective antibiotics, and incubated at 37°C. When an OD_600_ value of 1.2 was reached, the culture was induced with IPTG at a final concentration of 0.5 mM, and allowed to grow for 16 h at 15°C at 200 rpm. Then cells were harvested by centrifugation at 4,000 rpm for 20 min. The pellet was resuspended in a lysis buffer (50 mM Tris–HCl, 150 mM NaCl, 1 mM TCEP, pH 8.0). The cell lysate was prepared by sonication at 600 W for 10 min with 3 s bursts and 6 s cooling intervals followed by centrifugation at 13,000 rpm for 30 min at 4°C to pellet the cells. The supernatant was discarded, and inclusion bodies were collected. Protein was obtained from inclusion bodies and purified by Ni-NTA affinity chromatography according to the manufacturer’s protocol (Qiagen, Germany). All the protein purification steps were carried out at 4°C to avoid any degradation. Protein fractions were analyzed on 10% SDS-PAGE (GenScript, United States) stained with Coomassie Brilliant Blue G-250 (Bio-Rad, United States), and western blot analysis was done using a mouse anti-His monoclonal antibody (GenScript, United States).

### Photolyase Absorption and Action Spectra

Photolyase protein with 85% purity was subjected to spectroscopic measurements (Supplementary Figure S3). To identify the presence and type of chromophores responsible for photo-repair activity, the absorption, excitation and emission spectra were measured at 22°C with a Synergy H1 hybrid multi-mode plate reader (BioTek, United States). The excitation spectra (300–500 nm) of the purified protein and the denatured protein supernatant were measured at an emission wavelength of 530 nm, while the emission spectra (400–650 nm) were measured with an excitation wavelength of 370 nm. To release its chromophores, the protein was heated at 95°C for 5 min. The precipitated protein was then removed by centrifugation, and the supernatant was collected and used to measure the absorption spectrum of the released chromophores within the range of 300–550 nm wavelength. To obtain more precise information about the second chromophore (MTHF), fluorescence excitation and emission spectra were measured.

The photolyase action spectrum was determined *in vivo*. A separate, quantitative survival assay with the *E*. *coli* strain containing the photolyase gene (KY1225_pQE-30Xa_PN0912), was carried out in a similar manner as described in 2.3 with the exception that we here used five different incubation conditions: dark, 365, 400, 454, and 525 nm. Immediately after non-UV or UV-C (254 nm, 2 ± 0.2 μmol m^–2^ s^–1^ for 10 s) treatments, plates were transferred to complete darkness or one of the four different incubation wavelengths with an irradiance of 25 ± 5 μmol m^–2^ s^–1^ for 2 h at 25°C. After 2 h incubation in specified conditions, plates were incubated in darkness at 37°C overnight, and on the following day, the number of surviving colonies were recorded. The four incubation wavelengths were obtained from the following sources: UV-A, peak 365 nm, full width at half maximum (FWHM) 14 nm (RAY22 UV-A LEDs, Fluence Bioengineering, Austin, TX, United States); UV-A/blue, peak 400 nm, FWHM 14 nm (RAY22 UV-A LEDs, Fluence Bioengineering, Austin, TX, United States); blue, peak 454 nm, FWHM 23 nm (15 W GreenPower LED module HF blue; Philips, Netherlands) and green, peak 525 nm, FWHM 14 nm (RAY44 Green LEDs, Fluence Bioengineering, Austin, TX, United States) (Supplementary Figure S4).

A similar set up with slight modifications was used to study the action spectra for germination recovery of *P. neolycopersici* conidia treated with brief UV. Immediately after inoculation of *P. neolycopersici* conidia in water agar (1% w/v), Petri dishes without lids were exposed to either complete darkness (non-UV) or UV-C (peak 254 nm, 8 ± 0.2 μmol m^–2^ s^–1^ for 30 s) treatments. Immediately after treatments, plates were sealed and transferred to complete darkness or one of four different incubation wavelengths (365, 400, 454, and 525 nm) with an irradiance of 50 ± 5 μmol m^–2^ s^–1^ for 16 h followed by 8 h of darkness at temperature and relative humidity (RH) of 20 ± 1°C and 75 ± 5%, respectively. Germination of conidia was assessed 24 h after inoculation (Suthaparan et al., 2018).

### Analysis of Photolyase Gene Expression

Inoculum of a *P. neolycopersici* isolate (isolate was collected from Akershus county, Norway named as As_PN) was maintained on powdery mildew susceptible tomato cv. Espero, grown at a 16 h photoperiod at 20 ± 2°C and 75 ± 5% RH. For inoculation of tomato plants, diseased leaves were shaken thoroughly in distilled water containing Tween-20 (20 μl/L) and sprayed onto 2-week-old healthy plants using a handheld sprayer. Conidia of 9-day-old inoculum of *P. neolycopersici* was dusted on 2% (w/v) water agar in Petri dishes (54 dishes). After dusting, half of the dishes (27) were exposed to UV-C (254 nm, 8 ± 0.2 μmol m^–2^ s^–1^) for 30 s without lids, and the other half were exposed to complete darkness for 30 s without lids.

Immediately after this, the Petri dishes were sealed and distributed among one of the following three different incubation treatments: (i) darkness, (ii) UV-A/blue light (peak 400 nm, 50 ± 5 μmol m^–2^ s^–1^) (RAY22 UV-A LEDs, Fluence Bioengineering, Austin, TX, United States), or (iii) red light (peak 660 nm, 50 ± 5 μmol m^–2^ s^–1^) (10 W GreenPower LED module HF Deep Red; Philips, Netherlands). Samples were collected at three different time points during incubation: (i) 30 s, (ii) 4 h, and (iii) 8 h. Samples from three replicate Petri dishes were collected at each time point per treatment (biological replicates). Conidia were collected into 2 ml Eppendorf tubes using a microscopy glass slide to gently scrape the surface of the water agar. Samples were flash frozen with liquid nitrogen and then stored at −80°C until use. Total RNA was extracted using the Plant RNA reagent (Invitrogen, United States) followed by DNase treatment (Turbo DNA free^TM^ kit, Invitrogen, United States) and purification (PureLink RNA kit, Ambion, United States) as described in the manufacturer’s protocol. The quantity and quality of the RNA was estimated by NanoDrop^TM^ 2000 (Thermo Fisher Scientific, United States) and BioAnalyzer 2100 (Agilent Technologies Inc., United States). cDNA was synthesized from 500 ng of RNA using Superscript^TM^ IV VILO Master Mix (Invitrogen, United States).

Real-time PCR was performed using SYBR^TM^ Select Master Mix (Thermo Fisher Scientific, United States) on the 7500 Fast Real-Time PCR system (Applied Biosystems, United States). qPCR primers for *P. neolycopersici* photolyase (OINE01000912_T103440) (target gene) and alpha-tubulin gene (OINE01013217_T107300) (internal control) (Supplementary Data S1) were designed using Primer3 (v.0.4.0) (Supplementary Table S1). Standard curves were generated with one of the cDNA samples with serial dilution to a factor of 10. The following two-step PCR program of 50°C for 2 min, 95°C for 2 min, 45 cycles of 95°C for 15 s and 60°C for 1 min was used with three technical replicates per sample. Photolyase expression of *P. neolycopersici* was first normalized to alpha tubulin expression in the same sample, and then expression was calculated relative to the non-UV treated control samples, using the 2^–(ΔΔCt)^ method (Livak and Schmittgen, 2001).

### Recording Environmental Conditions and Data Analysis

Spectral composition and level of irradiance for all radiation sources used in this study were measured by an Optronic model 756 spectroradiometer (Optronic Laboratories, Orlando, FL, United States). Air temperature and RH inside controlled environment chambers were recorded in 5 min interval using a Priva greenhouse computer coupled with dry and wet bulb thermo sensors (Priva, Zijlweg, Netherlands). Analysis of variance for fold change gene expression of *P. neolycopersici* photolyase was done by using general linear model (Minitab Version 18.0, Minitab Corp., State College, PA, United States). Treatment means were separated by Tukey’s pairwise comparison at *P* = 0.05. All figures were drawn using SigmaPlot 10 (Systat Software, Inc., Chicago, IL, United States) unless otherwise specified.

## Results

### Phylogenetic Analysis

**FIGURE 1:**
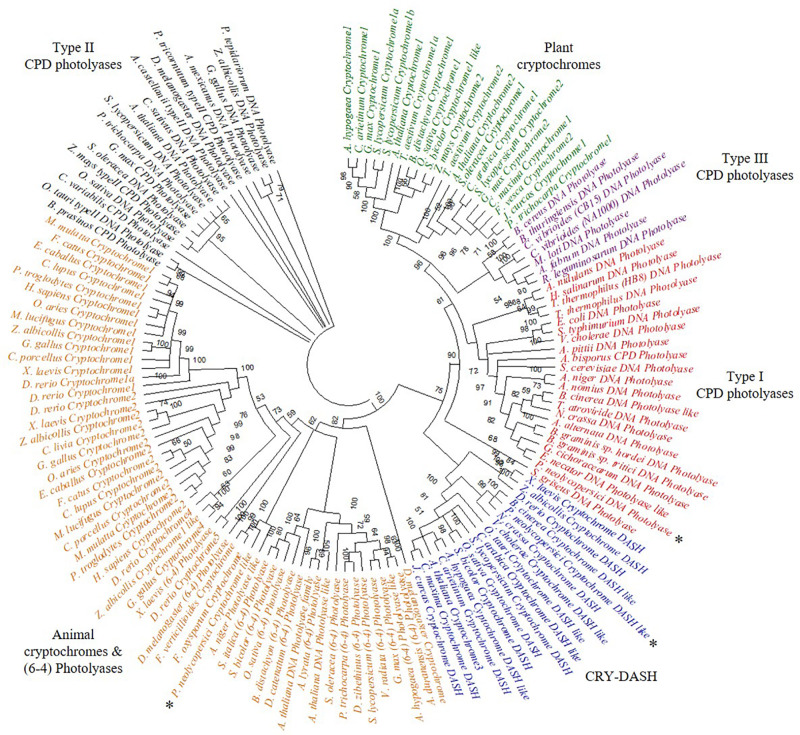
Phylogenetic analysis of cryptochrome/photolyase family (CPF)- like genes, including three CPF- like genes, OINE01015670_T110144 (*P. neolycopersici* cryptochrome like), OINE01000912_T103440 (*P. neolycopersici* photolyase like) and OINE01005061_T102555 (*P. neolycopersici* cryptochrome DASH like), all indicated by asterisk (*). An unrooted phylogenetic tree was constructed using neighbor-joining method. Bootstrap probability of 1000 replicates are expressed in percentage.

The phylogeny results suggest that *P. neolycopersici* gene OINE01015670_T110144 was clustered within the (6-4) photolyases and animal cryptochromes and appeared to be most closely related to other fungal cryptochromes in *Fusarium oxysporum* and *Aspergillus niger*. *P. neolycopersici* OINE01000912_T103440, falls within the clade of class I CPD photolyases with high bootstrap percentage and was closely related to fungal class I CPD photolyases, particularly from *E. necator* and *Golovinomyces cichoracearum*. The third CPF-like member from *P. neolycopersici*, OINE01005061_T102555, clustered within the CRY-DASH clade, and was closely related to the fungal *Neurospora crassa* and *Botrytis cinerea* cryptochrome DASH genes and the bacterial *Vibrio cholerae* cryptochrome DASH-like gene (Figure 1).

### Identification of a Functional Photolyase Gene of *P. neolycopersici* in *E. coli*

**FIGURE 2:**
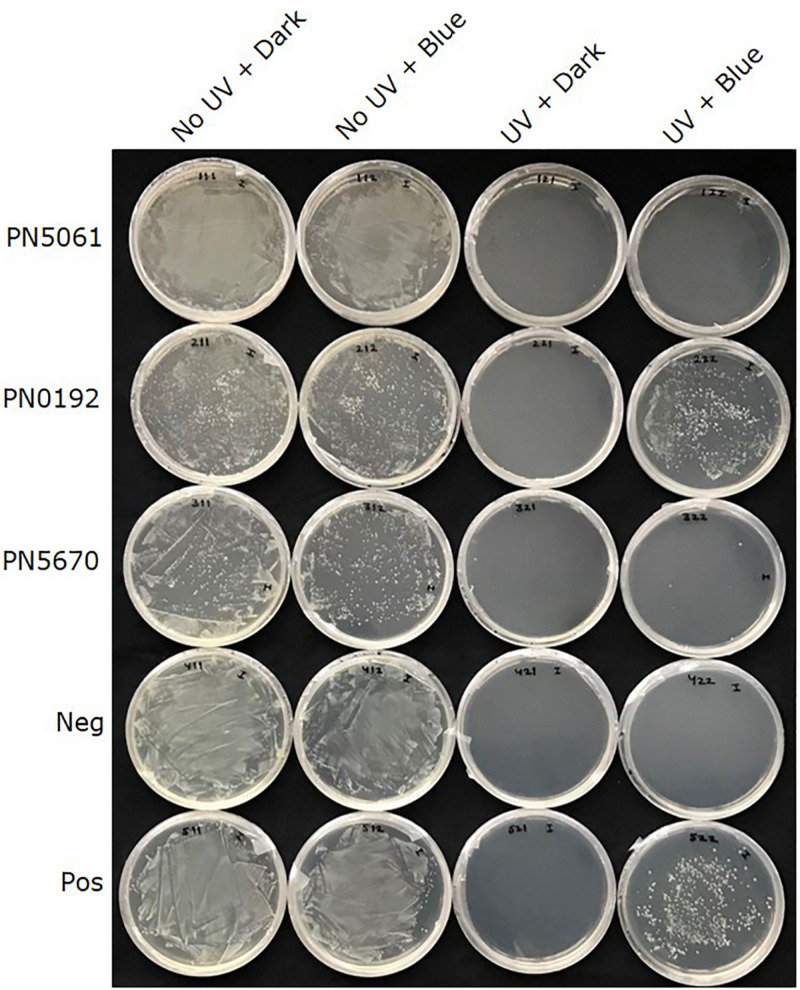
Qualitative survival assay of *Escherichia coli* strains KY1056 (photolyase-proficient, wild type) and KY1225 (photolyase-deficient, mutant) transformed with an empty pQE30-Xa vector served as positive (Pos) and negative (Neg) controls, respectively, and KY1225 transformed with the pQE30-Xa vector harboring codon optimized synthetic genes of PN5670, or PN0912, or PN5061. Actively growing transgenic strains were plated on LB agar (Amp^R^, Kan^R^) and exposed to either darkness or UV-C (254 nm of 2 ± 0.2 μmol m^– 2^ s^– 1^) for 10 s, followed by incubation in either darkness or blue light (454 nm of 25 ± 5 μmol m^– 2^ s^– 1^) for 2 h at 25°C and then overnight incubation at 37°C.

All three putative CPF- like genes (OINE01015670_T110144, OINE01000912_T103440, and OINE01005061_T102555) were tested for photoreactivation activity. In the qualitative survival assay, none of the tested *E*. *coli* strains survived when they were treated with brief UV-C followed by complete darkness. However, when the UV-C treatment was followed by 2 h in blue light (454 nm), KY1225 cells harboring the pQE-30Xa_PN0912 plasmid had a high survival rate, similar to that of the positive control. This treatment gave the lowest survival rate for KY1225 cells harboring either pQE-30Xa_PN5670 or pQE-30Xa_PN5061 plasmids, similar to that observed in the negative control (Figure 2).

**FIGURE 3:**
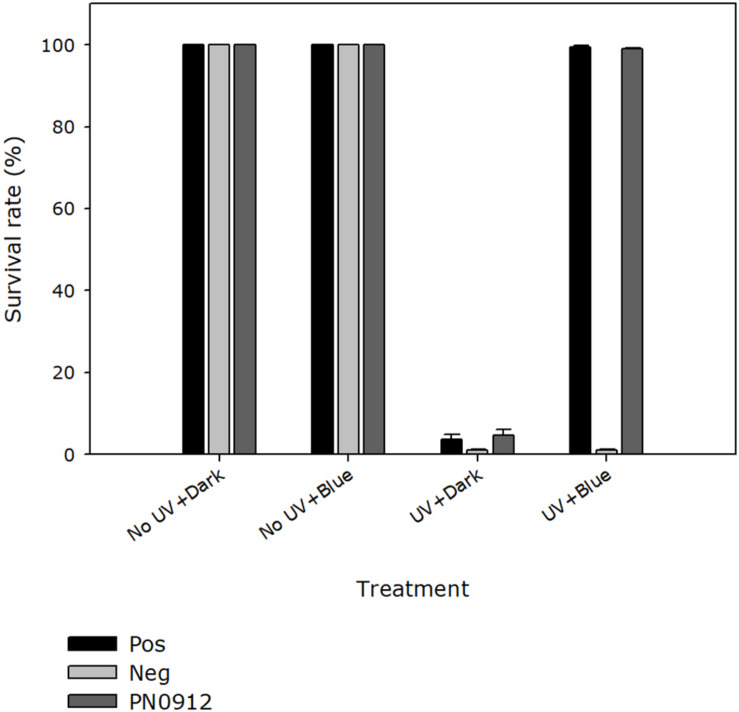
Quantitative survival activity assay of the *Pseudoidium neolycopersici* putative photolyase gene, PN0912. *Escherichia coli* strains KY1056 (photolyase-proficient, wild type) and KY1225 (photolyase-deficient, mutant) transformed with an empty pQE30-Xa expression vector served as positive (Pos) and negative (Neg) controls, respectively. KY1225 transformed with the expression vector containing the gene PN0912. Actively growing transgenic strains were plated on LB agar and exposed to either darkness or UV-C (254 nm of 2 ± 0.2 μmol m^– 2^ s^– 1^) for 10 s, followed by incubation in either darkness or blue light (454 nm of 25 ± 5 μmol m^– 2^ s^– 1^) for 2 h at 25°C and then overnight incubation at 37°C. The presence of photolyase activity was assessed by the presence of surviving colonies on Petri dishes containing LB agar (Amp^R^, Kan^R^). Percent survival of *E. coli* strain KY1225_pQE30Xa_PN0912 (PN0912) expressing a putative photolyase gene as well as the positive and negative control was calculated. Plates inoculated with fifty colonies each were exposed to the different treatments, and the number of surviving colonies were recorded. Each value is the mean ± standard error of two experiments with four replicate Petri dishes each (*n* = 8).

In the quantitative survival assay, *E*. *coli* strain KY1225 harboring pQE-30Xa_PN0912 and the positive control had a colony survival rate of more than 98% when they were treated with UV-C followed by blue light (454 nm) incubation, whereas a survival rate of 4.5% was observed when they were treated with UV-C followed by complete darkness (Figure 3 and Supplementary Figure S5). UV-C treatment, incubation wavelength and UV-C treatment × incubation wavelength, all had significant effect (*P* < 0.0001) on cell survival.

### Structural Prediction Model for *P. neolycopersici* Photolyas

**FIGURE 4:**
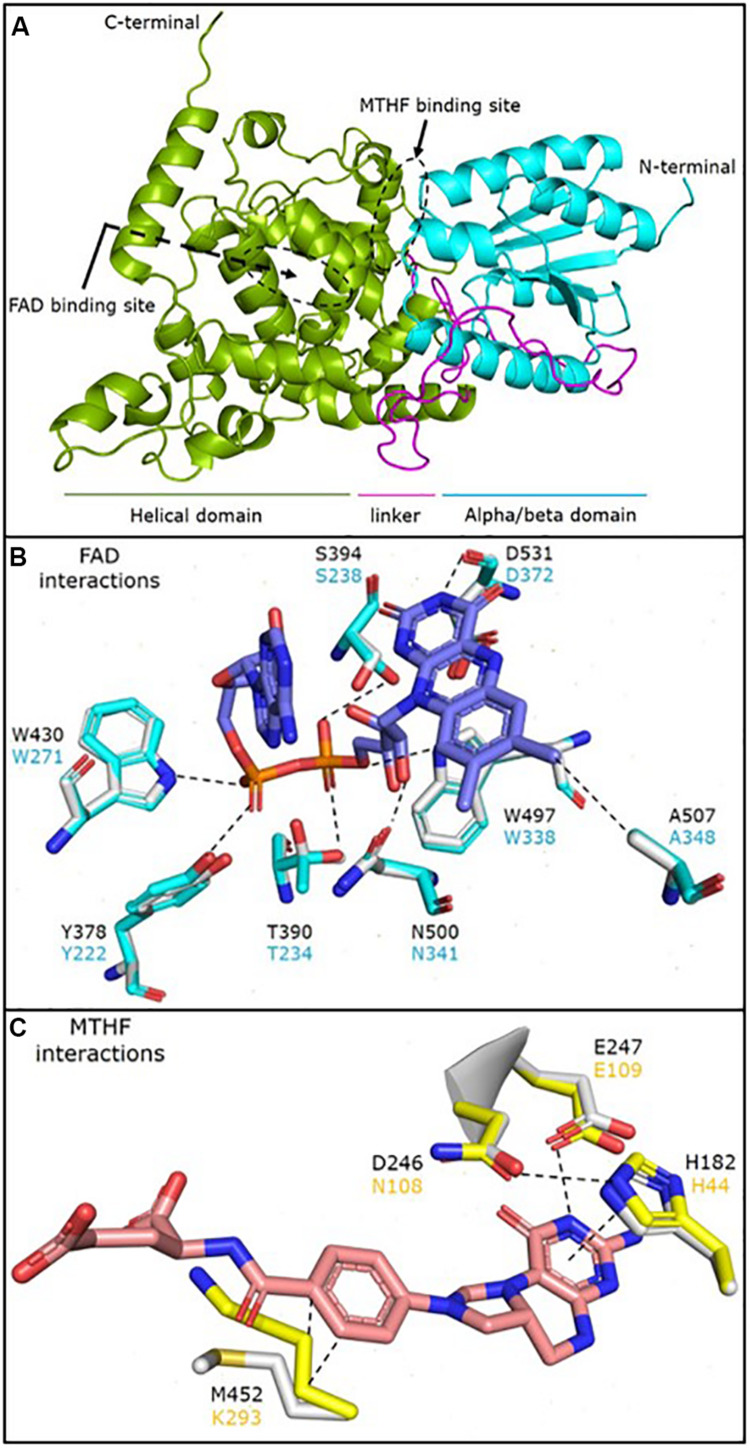
Cartoon representation of the predicted structural model of the *Pseudoidium neolycopersici* photolyase (OINE01000912_T103440), made using the protein structure and function prediction program RaptorX **(A)**. The structure shows an N-terminal alpha/beta domain (cyan) and a C-terminal helical domain (green) connected by a long linker (magenta). The flavin adenine dinucleotide (FAD) and 5, 10-methenyltetrahydrofolate (MTHF) binding sites are also observed on the protein surface (black dashed circles). Molecular interactions of **(B)** FAD and **(C)** MTHF with the neighboring residues in the *Escherichia coli* photolyase (cyan and yellow) superimposed onto the *P. neolycopersici* photolyase (light gray structure and black font). The dashed lines represent a stabilizing interaction between the two moieties. FAD is stabilized via extensive network of H-bonds with neighboring residues with the flavin head and phosphate groups. MTHF is stabilized by a stacking interaction with H182 and H-bond with E247, while the M452 stabilizes the MTHF tail by hydrophobic interactions. A nearly perfect overlay of the interacting residues suggests that the mode of FAD and MTHF binding in *P. neolycopersici* photolyase is similar to *E. coli* photolyase.

The structural prediction model for *P. neolycopersici* photolyase consisted of an N-terminal alpha/beta domain and a C-terminal helical domain with a long connecting loop (Figure 4A). Due to the lack of any sequence homology of the 130 amino acids in the N-terminal of the putative photolyase, only a structure model of amino acids 131–634 could be generated with high confidence (*P* < 0.0001). At the surface of the photolyase, two cavities were clearly exhibited, one at the center of the helical domain and the other in the cleft between the two domains (Figure 4A and Supplementary Figure S6). A 3D structural superimposition of *P. neolycopersici* photolyase with the *E. coli* photolyase (Park et al., 1995) showed perfect alignment of FAD and MTHF binding sites into the cavities without any clashes with neighboring residues (Supplementary Figure S6). However, a structural superimposition with the 8-HDF-containing photolyase from *A. nidulans* (Tamada et al., 1997) only demonstrated an alignment of the FAD binding site.

Analysis of the amino acid residues involved in the interactions of FAD and MTHF in the photolyase structure showed that all residues involved in FAD binding in *P. neolycopersici* photolyase were completely conserved to FAD binding in *E*. *coli* (Figure 4B) and *A*. *nidulans* (Supplementary Figure S7). The photolyase of *P. neolycopersici* accommodated MTHF in the binding site via His and Glu residues homologous to the photolyase of *E. coli*. However, the N108 (Asn) and K293 (Lys) residues were replaced by D246 (Asp) and M452 (Met) residues, respectively (Figure 4C and Supplementary Figure S7).

### Absorption, Excitation and Emission Spectra of *P. neolycopersici* Photolyase

**FIGURE 5:**
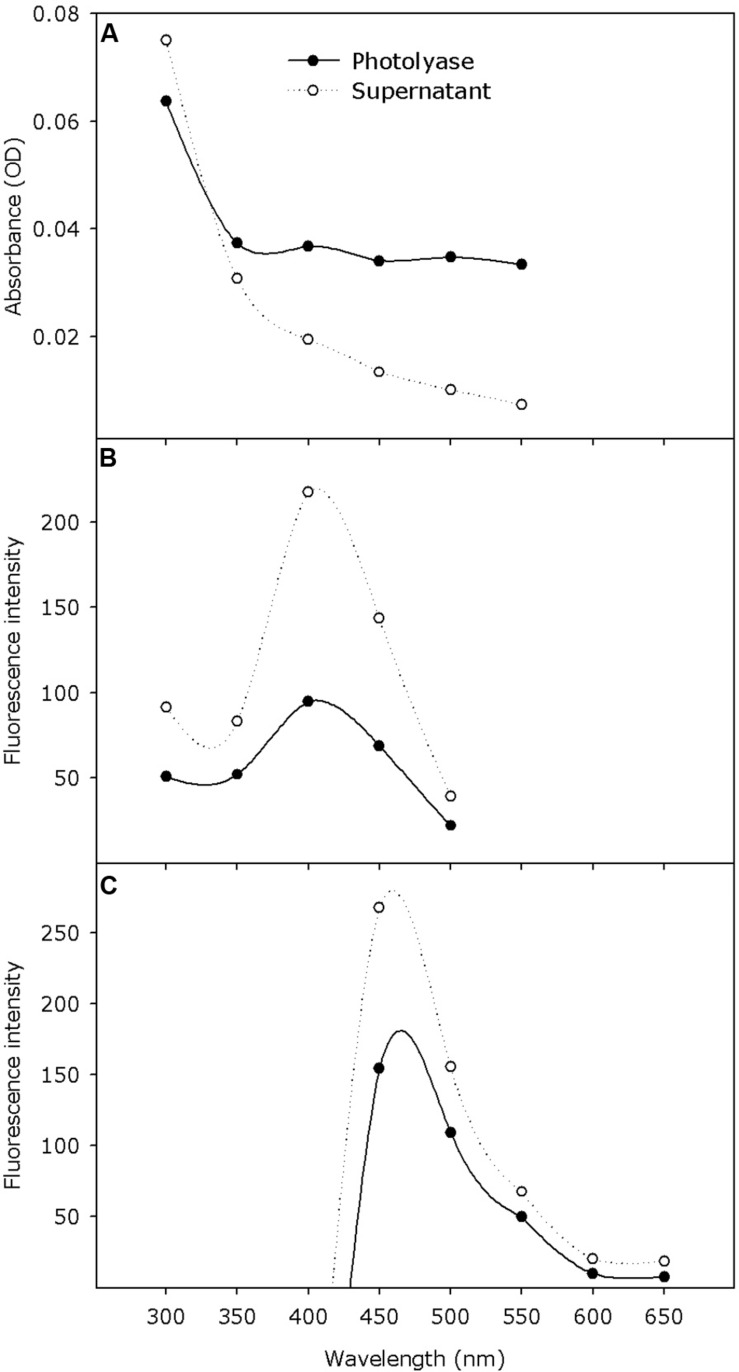
Absorption and fluorescence excitation and emission spectra of purified *Pseudoidium neolycopersici* photolyase protein (solid line) and denatured protein supernatant (dotted line) containing the released chromophore. **(A)** Absorption spectra (scans from 300–550 nm) were recorded at pH 8.0 in storage buffer. **(B)** Excitation spectra (scans from 300–500 nm) were recorded with the emission wavelength adjusted to yield maximum fluorescence at 530 nm. **(C)** Emission spectra (scans from 400–650 nm) were recorded with the excitation wavelength at 370 nm.

The absorption spectra showed some absorbance in the measured wavelength range (300–550 nm) with the tendency of strong absorbance around 300 nm (Figure 5A). The excitation spectra showed that the maximum fluorescence (measured at 530 nm) was obtained at an excitation wavelength of 410 nm (Figure 5B). Upon excitation with 370 nm wavelength, an emission peak at 460 nm was observed (Figure 5C).

### Action Spectra of *P. neolycopersici* Photolyase

**FIGURE 6:**
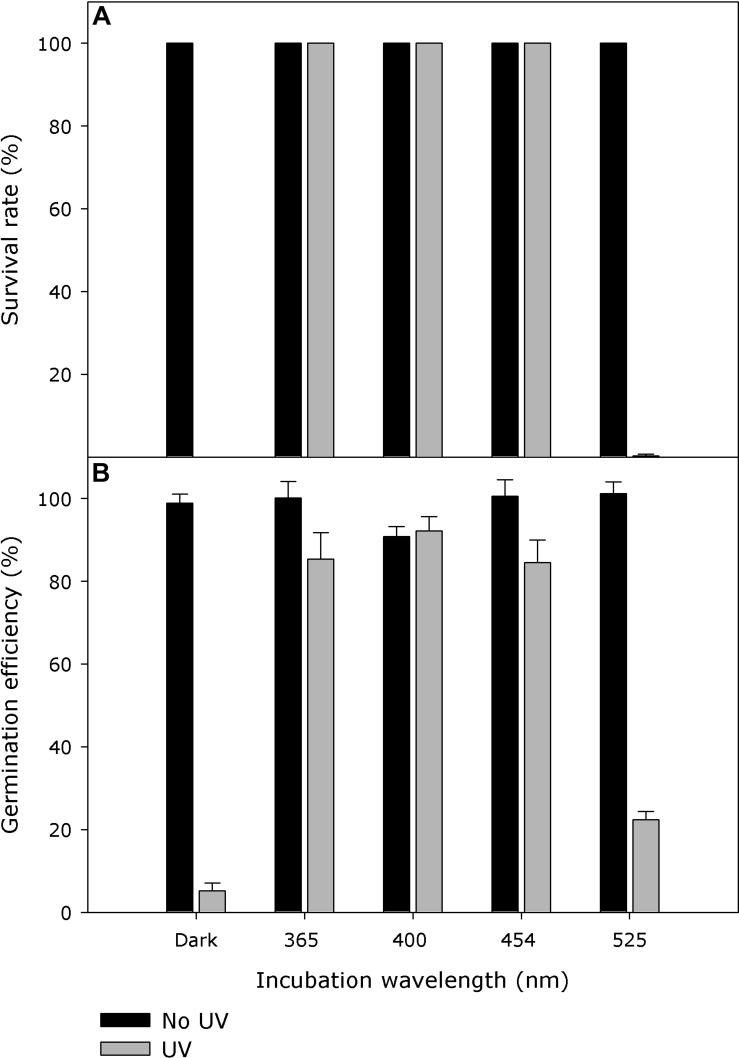
**(A)** Action spectra of *Pseudoidium neolycopersici* photolyase gene, PN0912. *Escherichia coli* strain KY1225 (photolyase-deficient) transformed with the expression vector pQE30Xa containing *P. neolycopersici* photolyase, PN0912, were used to inoculate LB agar (Amp^R^, Kan^R^) petri dishes (50 colonies per plate). Plates were exposed for 10 s to either darkness or UV-C (254 nm, 2 ± 0.2 μmol m^– 2^ s^– 1^), followed by incubation in either darkness, 365, 400, 454, or 525 nm (all 25 ± 5 μmol m^– 2^ s^– 1^) for 2 h at 25°C, and then in darkness overnight at 37°C. Each value is the mean colony survival rate ± standard error of six replicate Petri dishes (*n* = 6). **(B)** Recovery action spectra of *P. neolycopersici*. Conidia of *P. neolycopersici* were dusted on water agar and exposed to either (i) complete darkness (non-UV) or (ii) UV-C (UV, with a peak of 254 nm, 8 ± 0.2 μmol m^– 2^ s^– 1^) for 30 s. All treatments were followed by incubation in darkness (Dark) for 24 h, or irradiation of 365, 400, 454, or 525 nm (all 50 ± 5 μmol m^– 2^ s^– 1^) for 16 h followed by 8 h in darkness. Each value is the mean germination efficiency ± standard error of six replicate Petri dishes (*n* = 6).

Within the tested incubation wavelength ranges, *E. coli* strain KY1225 harboring pQE30Xa_PN0912 showed a survival rate of more than 99% when it was incubated with wavelengths of 365, 400, or 454 nm immediately after UV treatment. The survival rate was <1% when it was incubated with 525 nm wavelength or complete darkness immediately after UV treatment. UV-C treatment, incubation wavelength and UV-C treatment × incubation wavelength, all had significant effect (*P* < 0.0001) on cell survival (Figure 6A and Supplementary Figure S8). Action spectra for germination recovery in *P. neolycopersici* showed similar results (Figure 6B).

### Expression of the Photolyase Gene in Conidia of *P. neolycopersici*

**FIGURE 7:**
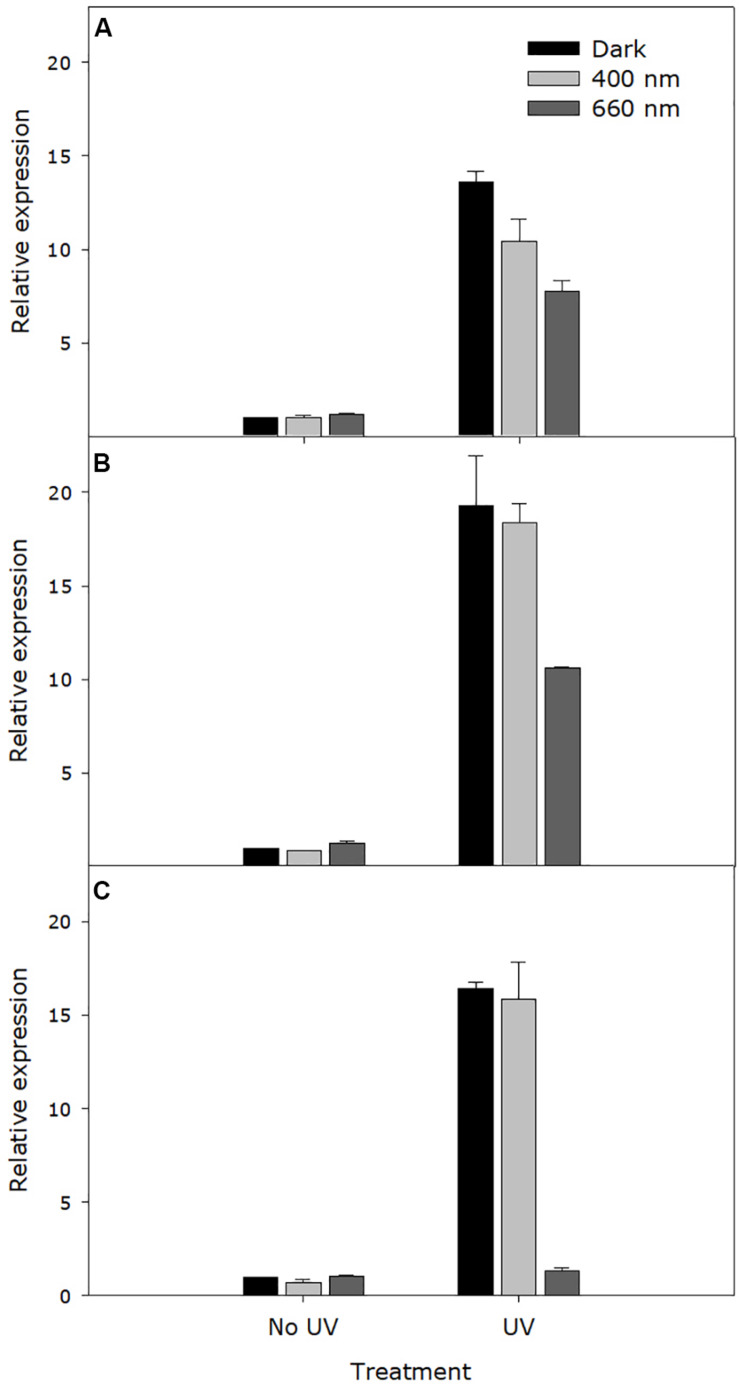
Expression level of the *Pseudoidium neolycopersici* photolyase gene, OINE01000912_T103440. Conidia of *P. neolycopersici* were exposed for 30 s to either complete darkness (control treatment) or UV-C treatment (UV, 254 nm of 8 ± 0.2 μmol m^– 2^ s^– 1^), and then immediately incubated either in the dark or under UV-A/blue (peak of 400 nm of 50 ± 5 μmoles m^– 2^ s^– 1^) or red light (peak 660 nm of 50 ± 5 μmoles m^– 2^ s^– 1^). During incubation, samples were collected at three different time points: **(A)** 30 s, **(B)** 4 h, and **(C)** 8 h. Expression levels were normalized to the corresponding alpha-tubulin gene expression and are shown as fold-change relative to the expression in non-UV-treated (control treatment) samples. Each value is the mean ± standard error of three independent biological replicates (*n* = 3), each with three technical replicates.

Independent of incubation wavelength, expression of the photolyase gene, OINE01000912_T103440, was significantly higher in UV-C treated samples within 30 s. The maximum expression level was reached at 4 h after UV-C treatment. The photolyase gene expression was significantly higher in darkness and in UV-A/blue light than in red light at all sampling times (30 s, 4 h, and 8 h) after UV-C treatment (*P* < 0.0001), particularly at the latter time points. When incubated in red light, expression in UV-C treated samples dropped to the same level as the non-UV control (Figure 7).

## Discussion

Among the three tested CPF-like genes identified in the *P. neolycopersici* genome, this study confirmed the presence of one photolyase gene with functional similarity to previously well characterized protein in other life forms. Survival of a photolyase deficient *E*. *coli* strain transformed with OINE01000912_T103440 under UV-C treatment followed by blue light incubation showed that this candidate gene is a functional photolyase. The non-surviving photolyase deficient *E*. *coli* strain transformed with the other two candidate genes exposed to the same conditions suggested that those two genes could be the other members of cryptochrome/photolyase family, either cryptochrome or CRY-DASH genes, which do not possess photoreactivation activity (Thompson and Sancar, 2002). Our phylogenetic analysis suggests that OINE01015670_T110144 and OINE01005061_T102555 are putative cryptochrome and CRY-DASH, respectively (Figure 1). However, further studies are required to confirm their functional characteristics in *P. neolycopersici*. The purified recombinant *P. neolycopersici* photolyase protein showed a molecular mass of about 72 kDa, including a 6× His-tag. This matches the molecular mass of 71.87 kDa, calculated from the deduced amino acid sequence of 634 residues. This is slightly larger than photolyases from other species in both number of amino acid residues and molecular mass (Sancar et al., 1987b; Yajima et al., 1991; Waterworth et al., 2002). The 6× His-tag facilitates single step protein purification without interfering in the folding of protein, thereby its activity (Malhotra, 2009).

The cryptochrome/photolyase superfamily comprises six major subgroups distinguished based on their evolutionary relationship and functions; class I CPD photolyases, class II CPD photolyases, class III CPD photolyases, plant cryptochromes, CRY-DASH proteins and animal cryptochromes which also include (6-4) photolyases (Bayram et al., 2008; Mei and Dvornyk, 2015). Phylogenetic analysis showed that *P. neolycopersici* photolyase classified as a class I CPD photolyase, and this class includes extensively studied *E. coli* CPD photolyase and photolyases from other ascomycetes. Class I CPD photolyases are most prevalent in micro-organisms (Essen and Klar, 2006). All class I photolyases contain an identical catalytic cofactor (FAD) and an additional second cofactor which is either MTHF or 8-HDF, responsible for harvesting light. Depending on the presence of a second chromophore; either 8-HDF or MTHF, class I CPD photolyases are further divided into flavin (8-HDF) and folate (MTHF) types (Sancar, 2003).

Structural prediction model analysis suggested that *P. neolycopersici* photolyase is capable of binding FAD and MTHF as cofactors. This is in accordance with identified cofactors in the majority of photolyases, including in *E*. *coli* (Park et al., 1995). The photolyase of *E. coli* accommodates MTHF in the binding site via four crucial residues (His44, Asn108, Glu109, and Lys293) (Park et al., 1995). Our results suggest that the photolyase of *P. neolycopersici* binds MTHF via His182, Asp246, Glu247, and Met452. Interestingly, this change in the interacting residues would not affect the bonding pattern, as the Asn108 and Lys293 in *E. coli* stabilizes the MTHF via one oxygen mediated H-bond and hydrophobic interactions, respectively, which would also form with the Asp246 and Met452 in the *P. neolycopersici* photolyase (Figure 4C). Glu109, which is involved in MTHF interactions and was conserved here, is proposed to be conserved in all folate classes of photolyases except in the photolyase from *Bacillus firmus* (Park et al., 1995). A limited number of species, including *A. nidulans* and *Thermus thermophilus*, have 8-HDF as second cofactor (Tamada et al., 1997; Klar et al., 2006). Our structural prediction model did not predict the presence of 8-HDF in *P. neolycopersici* photolyase. Crystal structures of photolyases from *E*. *coli, A. nidulans*, and *T. thermophilus* showed that the structures of all these photolyases are remarkably similar with only about 25% sequence identity, as in the case of *P. neolycopersici* photolyase (Sancar, 2004).

The presence of the FAD cofactor was further corroborated with optical absorption properties of *P. neolycopersici* photolyase. However, we were not able to detect any clear maximum in the absorption spectrum. Absorption properties of the supernatant showed that the cofactor was in a reduced form. Observations in our study were similar to the emission of fully reduced FADH^–^, which is a catalytically active form that can be reduced by photo- or chemical reduction (Sancar et al., 1987a; Malhotra et al., 1994; Kao et al., 2008). Previous studies reported that an emission peak at 530 nm with an excitation wavelength of 370 nm is an indication of the presence of FAD_ox_ and is characteristic to flavin and MTHF chromophores (Worthington et al., 2003; Teranishi et al., 2008; Tagua et al., 2015). Fluorescence spectra did not show any evidence for the presence of an additional chromophore. Previous studies also showed that FAD could be identified as the only chromophore present when proteins are heterologously expressed (Kleiner et al., 1999). It has been reported that when *E. coli* photolyase was expressed heterologously, approximately 50–70% MTHF was lost during purification (Payne and Sancar, 1990; Sancar, 2003).

This study indicates that the photolyase of *P. neolycopersici* has a broad action spectrum ranging from around 365 to 454 nm. This action spectrum perfectly overlaps with the previously published germination recovery action spectra of *P. neolycopersici* (Figure 6B) (Suthaparan et al., 2018). The UV-A/blue range (peak wavelengths of 365 and 400 nm) resulted in larger colonies as compared to pure blue light (peak 454 nm) (Supplementary Figure S9). This suggests that UV-A is the most effective wavelength for the functional activity of photolyase in *P. neolycopersici*. All folate (MTHF) classes of photolyases have absorption maxima in the range of 370–440 nm, as reported for *E. coli*, *N. crassa* and *Saccharomyces cerevisiae*, indicating that the folate class has a considerably broader action spectrum (366–450 nm) (Sancar et al., 1987a; Sancar, 1990; Eker et al., 1994). On the contrary, the absorption maximum of the flavin-type (8-HDF) chromophore in *A. nidulans*, *Streptomyces griseus*, and *Methanobacterium thermoautotrophicum* falls between 434 and 443 nm, showing a very narrow range of its action spectrum (Hada et al., 2000; Sancar, 2003). The photolyase action spectrum of *P. neolycopersici* (365–454 nm) and the protein structure model are in agreement with the folate class of photolyases. However, confirmation of the presence of a second chromophore needs further investigation with the native protein isolated from *P. neolycopersici*.

Quantitative PCR results showed that the gene expression of photolyase in *P. neolycopersici* was induced by brief exposure to UV-C. In most fungi, light induces both the expression of genes involved in protection against UV-induced DNA damage and synthesis of protective pigments that can filter out harmful radiation (Braga et al., 2015). Blue light responses are well studied in many fungi, such as *N. crassa*, *Aspergillus nidulans* and *Phycomyces blakesleeanus* (Corrochano, 2011; Fuller et al., 2015). Photoreceptor genes that are involved in blue light perception showed upregulation by blue light, and it was reported that the blue light receptor Wco1 regulates the photolyase gene expression in *Ustilago maydis* (Brych et al., 2016). In *Trichoderma atroviride*, UV-A and blue light induce the *phr1* expression through the white collar complex BLR1/BLR2 (Berrocal-Tito et al., 2007). Our study showed that transcriptional upregulation of *P. neolycopersici* photolyase starts immediately after the brief UV-C exposure, both under subsequent incubation in darkness and in UV-A/blue light, and the upregulated expression is maintained for at least 8 h. Expression was also upregulated under subsequent incubation in red light, but after 8 h it was back to the level of the non-UV treated control. Previously, we have shown that incubation of UV-damaged *P. neolycopersici* conidia in UV-A/blue light can mediate gemination recovery of conidia, while incubation in darkness or red light cannot (Suthaparan et al., 2018). Taken together, our results suggest that (1) photolyase gene expression is induced by the brief UV-C exposure itself and that photolyase gene expression alone is not sufficient for gemination recovery of conidia, (2) UV-A/blue light provides the necessary energy for functional activity of photolyase that mediates germination recovery, (3) the inability of red light to provide necessary energy for functional activity of photolyase may prevent germination recovery, (4) red light dependent downregulation of the photolyase expression, which was induced by UV-C may further enhance this effect. This explains the increased efficacy of UV-C against powdery mildew when used in combination with red light (Suthaparan et al., 2014, 2018). A recent study also showed that UV-C induces the transcription of three putative photolyase genes in *Blumeria graminis*, the causal organism of barley powdery mildew (Zhu et al., 2019).

The study did not provide functional evidence of photolyase directly from the natural host *P*. *neolycopersici*. Host induced gene silencing (HIGS) employing RNA silencing mechanisms, has been used widely in functional genomics, especially in silencing the targets of invading pathogens including powdery mildews (Pliego et al., 2013; Qi et al., 2019). The follow-up studies will focus on using host induced silencing of *P*. *neolycopersici* photolyase to provide direct functional evidence in its natural host.

The photoreactivation process plays an important role in UV-based disease control in crops; hence, planning accurate irradiation applications is critical and is highly dependent on lighting conditions. The present findings about the action spectra of photolyase in *P. neolycopersici* will be very helpful in designing efficient UV-mediated disease suppression strategies for greenhouse-grown crops, providing an environmentally friendly alternative for the management of powdery mildew diseases.

## Data Availability Statement

The contig and coding sequence data of the *P. neolycopersici* CPF like genes used in this study are deposited in the NCBI database under the accession numbers MT277359, MT277360, MT277361, MT277362, MT277363, and MT277364. The raw data supporting the conclusions of this article will be made available by the authors, without undue reservation, to any qualified researcher.

## Author Contributions

RP designed and executed the experiments and wrote the manuscript. ASu conceived, designed, executed the experiments, and revised the manuscript. ÅE, ASt, HG, KS, and LC-D contributed in designing of experiments, and critical revision of the manuscript.

## Conflict of Interest

The authors declare that the research was conducted in the absence of any commercial or financial relationships that could be construed as a potential conflict of interest.

## Footnotes

1 https://www.ncbi.nlm.nih.gov/

2 http://www.rcsb.org

## Abbreviations

CPD: cyclobutane pyrimidine dimer
CPF: cryptochrome/photolyase family
8-HDF, 8-hydroxy-7: 8-didemethyl-5-deazariboflavin
FAD: flavin adenine dinucleotide
IPTG: isopropyl-1-thio-D-galactopyranoside
LB: Lysogeny Broth
MTHF: methenyltetrahydrofolate
PHR: photoreactivation
UV: ultraviolet.
