# Supplementary material for: Functional Characterization of Pseudoidium neolycopersici Photolyase Reveals Mechanisms Behind the Efficacy of Nighttime UV on Powdery Mildew Suppression
Source: Front Microbiol. 2020 May 29;11:1091. doi: 10.3389/fmicb.2020.01091 (PMC7272715; doi:10.3389/fmicb.2020.01091)
Supplement: Supplementary file 1 [file Data_Sheet_1.docx]

**Supporting data**

**Table S1. Primers used in pCR2.1- TOPO TA cloning and qRT-PCR**

| **TOPO TA cloning** | |
| --- | --- |
|  | |
| Gene: OINE01015670_T110144 | |
| **Primer Name** | **Sequence (5' ─> 3')** |
| Pn_PHR-CRY 1_cF | AAGCTTGCATGCGGGAAATCGAGAGTCTTGTAT |
| Pn_PHR-CRY 1_cR | CGTCTTCCCGGGTTATTTGTGATCACACGTCTT |
|  |  |
| Gene: OINE01000912_T103440 | |
| **Primer Name** | **Sequence (5' ─> 3')** |
| Pn_PHR-CRY 2_cF | AAGCTTGCATGCGAGTCAAAGTCTTCTACTACA |
| Pn_PHR-CRY 2_cR-R | CGTCTTGTCGACTCAATTACTCGCTCTTCCAAG |
|  |  |
| Gene: OINE01005061_T102555 | |
| **Primer Name** | **Sequence (5' ─> 3')** |
| Pn_PHR-CRY 3_cF | AAGCTTGCATGCACGGAGATAAAAAGGCTGCTA |
| Pn_PHR-CRY 3_cR | CGTCTTCCCGGGATCAACCCATTTATTTGATGC |
|  |  |
|  |  |
| **qRT-PCR** | |
|  |  |
| Gene: OINE01013217_T107300 | |
| **Primer Name** | **Sequence (5' ─> 3')** |
| αTubulin_F | TAATTCCTCGGGACTGCAAC |
| αTubulin_R | CATCATCGGGTGAAGAAGGT |
|  |  |
| Gene: OINE01000912_T103440 | |
| **Primer Name** | **Sequence (5' ─> 3')** |
| qPn_0912_F | CTCATTAAGCTCCCGGACTG |
| qPn_0912_R | ATTAGCGTACTCGGGCTTGA |


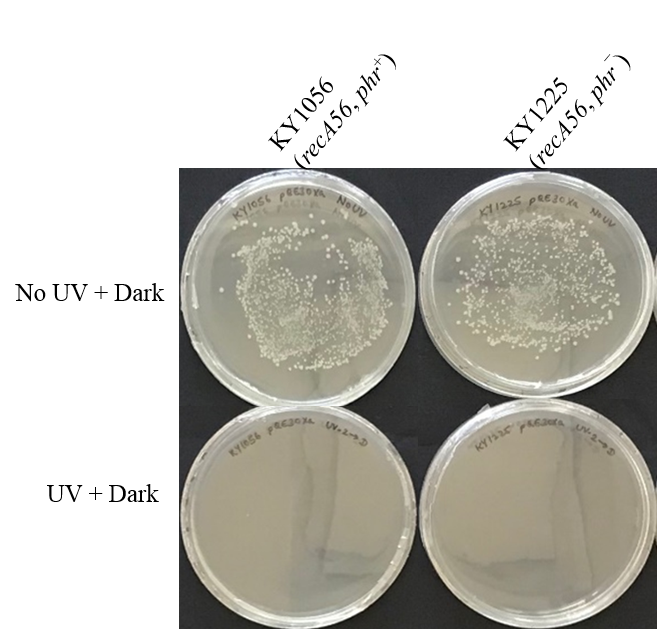


**Fig. S1.** UV dose tested on *Escherichia coli* strains KY1056 (*recA56*, *phr*^+^) and KY1225 (*recA56*, *phr*^¯^) transformed with pREP4 (Kan^R^) and pQE-30Xa (Amp^R^). Transformed *E. coli* strains were treated with brief UV-C, peak 254 nm of 2 ± 0.2 µmoles m^2^ s^-1^ for 10 s and immediately incubated at 37°C in dark for overnight. Next day, plates were assessed for surviving colonies.


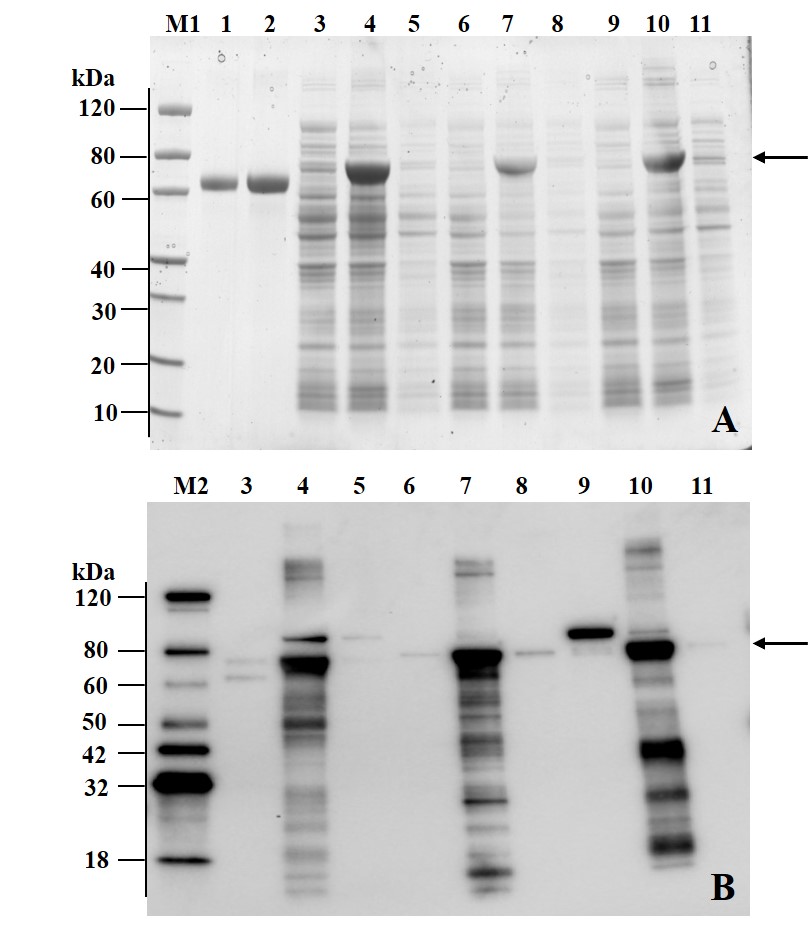


**Fig. S2**. SDS-PAGE and Western blot of three recombinant *Escherichia coli* strains transformed with the constructs (pQE-30Xa_PN5670, pQE-30Xa_PN0912 and pQE-30Xa_PN5061) carrying *Pseudoidium neolycopersici* cryptochrome/photolyase family like genes. (A) Coomassie-stained SDS-PAGE. (B) Western blot detection of His-tagged proteins by Mouse-anti-His mAb (GenScript, cat. no. A00186). Arrows indicate the bands corresponding to the size of 73.28 kDa, 72.69 kDa and 75.14 kDa for pQE-30Xa_PN5670, pQE-30Xa_PN0912 and pQE-30Xa_PN5061 proteins respectively, including 6X-His tag. M1- Protein Marker (GenScript, cat. no. M00516), M2- Protein Marker (GenScript, cat. no. M00521). Lane 1, 2- Bovine Serum Albumin 1.0 µg and 2.0 µg. Lane 3, 6, 9- Cell lysate of non-induced strains carrying pQE-30Xa_PN5670, pQE-30Xa_PN0912 and pQE-30Xa_PN5061, respectively. Lane 4, 7, 10- Cell lysate after induction of expression in strains carrying pQE-30Xa_PN5670, pQE-30Xa_PN0912 and pQE-30Xa_PN5061, respectively. Lane 5, 8, 11- Supernatant of cell lysate after induction of strains carrying pQE-30Xa_PN5670, pQE-30Xa_PN0912 and pQE-30Xa_PN5061, respectively.


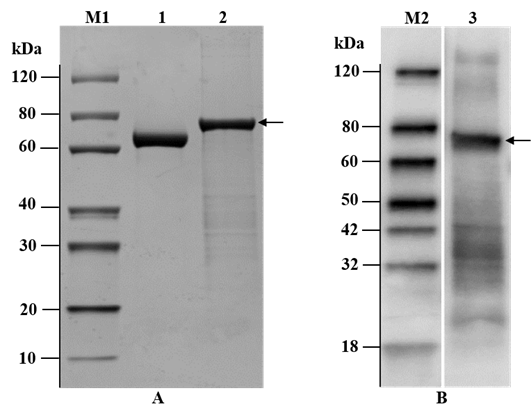


**Fig. S3.** SDS-PAGE and Western Blot analysis of *Pseudoidium neolycopersici* photolyase (pET-30a_PN0912) protein purified by Ni-NTA spin column (Qiagen, Germany). (A) Coomassie- stained SDS- PAGE gel. M1- Protein Marker (GenScript, cat. no. M00516), Lane 1- Bovine Serum Albumin (2.0 µg), Lane 2- *P. neolycopersici* photolyase (2.0 µg). (B) Western blot analysis. M2- Protein Marker (GenScript, cat. no. M00521), Lane 3- Detection of His-tagged photolyase by Mouse-anti-His mAb (GenScript, cat. no. A00186). Arrows indicate *P. neolycopersici* photolyase corresponding to a size of 72.69 kDa including 6X-His tag.

**Fig. S4.** Spectral distribution of optical radiation sources used in this study, measured at 1 nm intervals (peak wavelengths in parentheses). UV-C (254 nm) used for brief UV treatments, and UV-A (365 nm), UV-A/blue (400 nm), blue (454 nm), green (525 nm), red (660 nm) lights used in recovery of *Escherichia coli* and in quantitative RT-PCR with *Pseudoidium neolycopersici.*


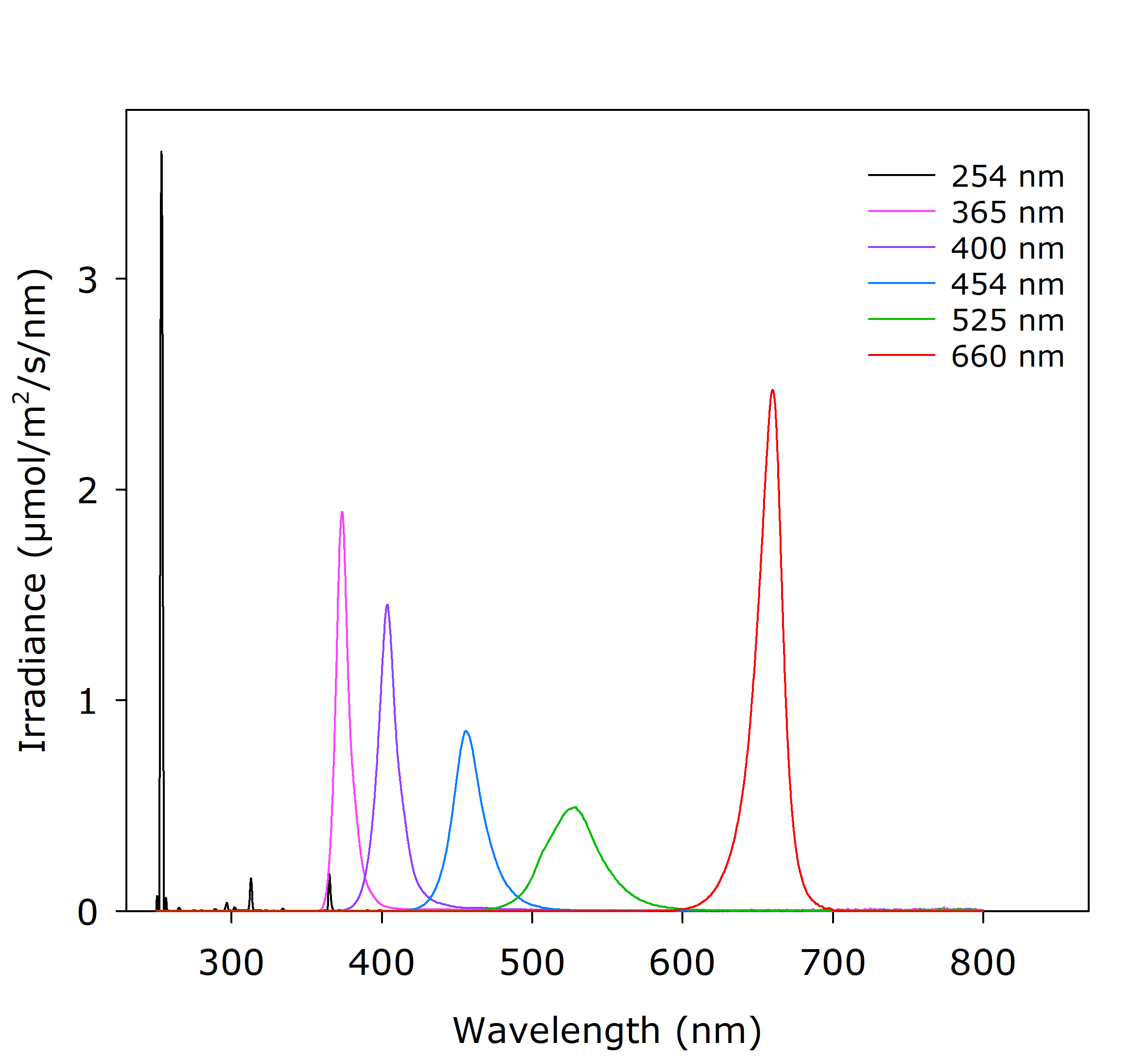


**Fig. S5.** The effect of incubation wavelengths, dark and 454 nm immediately after brief UV treatment on recombinant *Escherichia coli* strains: (A) positive control KY1056 (photolyase-proficient, wild type) and (B) negative control KY1225 (photolyase-deficient, mutant), each transformed with an empty expression vector, pQE-30Xa; and (C) KY1225_pQE-30Xa_PN0912 expressing functional photolyase gene from *Pseudoidium neolycopersici*. 50 colonies were picked up and inoculated on a fresh LB agar (Amp^R^, Kan^R^) plate and exposed to either darkness (non-UV) or UV-C (peak 254 nm of 2 ± 0.2 µmol m^-2^ s^-1^ for 10 s). After UV treatment, samples were immediately incubated with blue light (peak 454 nm of 25 ± 5 µmol m^-2^ s^-1^ ) for 2 h at 25°C followed by incubation at 37°C for overnight.


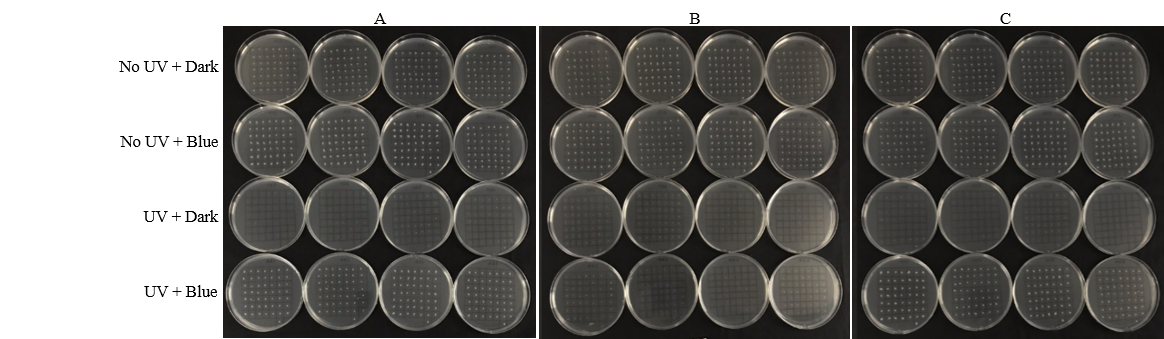


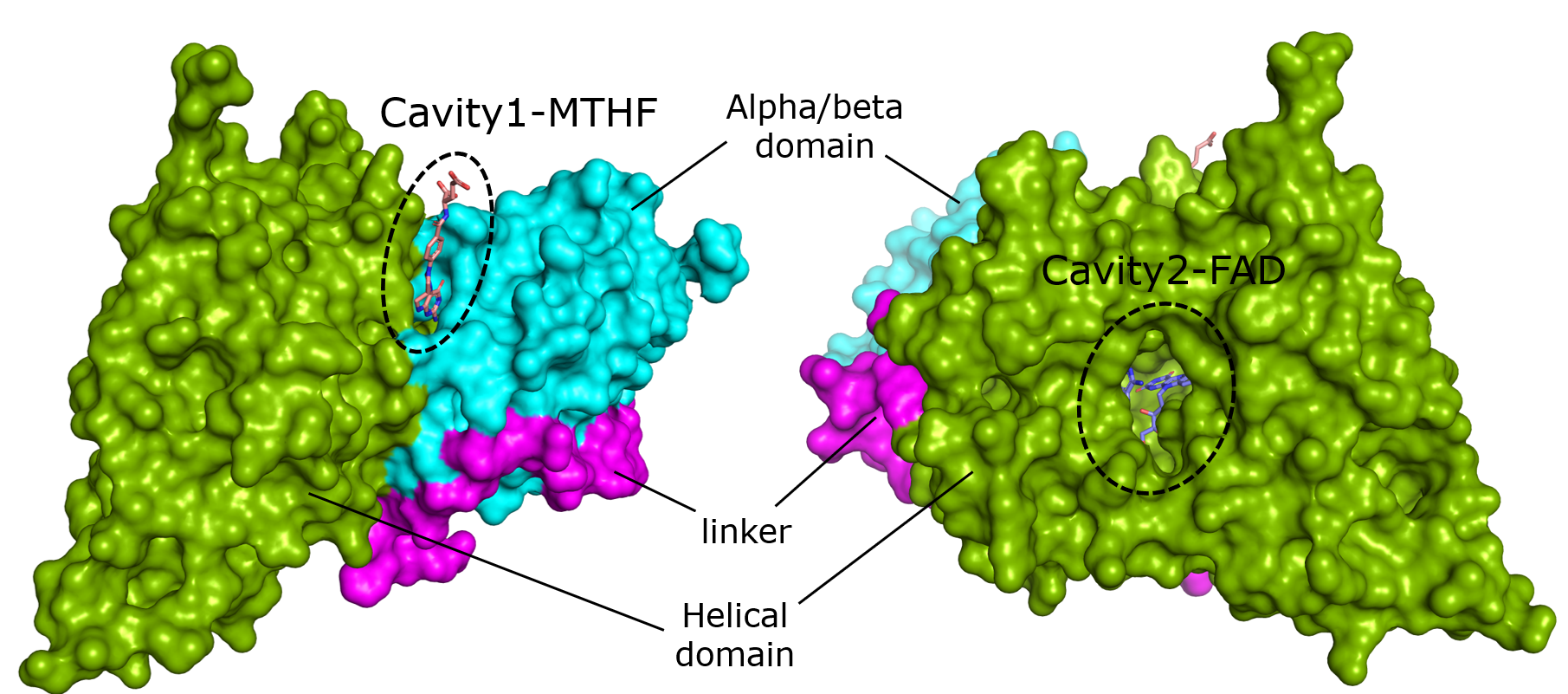


**Fig. S6.** The surface representation of the *Pseudoidium neolycopersici* photolyase showing (dashed circles) (1) Methenyltetrahydrofolate (MTHF) binding cavity and (2) Flavin adenine dinucleotide (FAD) binding cavity. Two domains (alpha/beta and helical) and the linker are colored in cyan, green and magenta, respectively, and a 3D superimposition with *Escherichia coli* photolyase bound with FAD and MTHF corroborates the nature of each binding cavities. After 3D superimposition only two co-factors from *E.coli* structure is shown, which superimpose perfectly into the binding pockets on the *P. neolycopersici* photolyase structure. The MTHF head group goes deep into the cleft between the two domains, while a deep cavity in the center of the helical domain (green) accommodate FAD without any structural clashes.


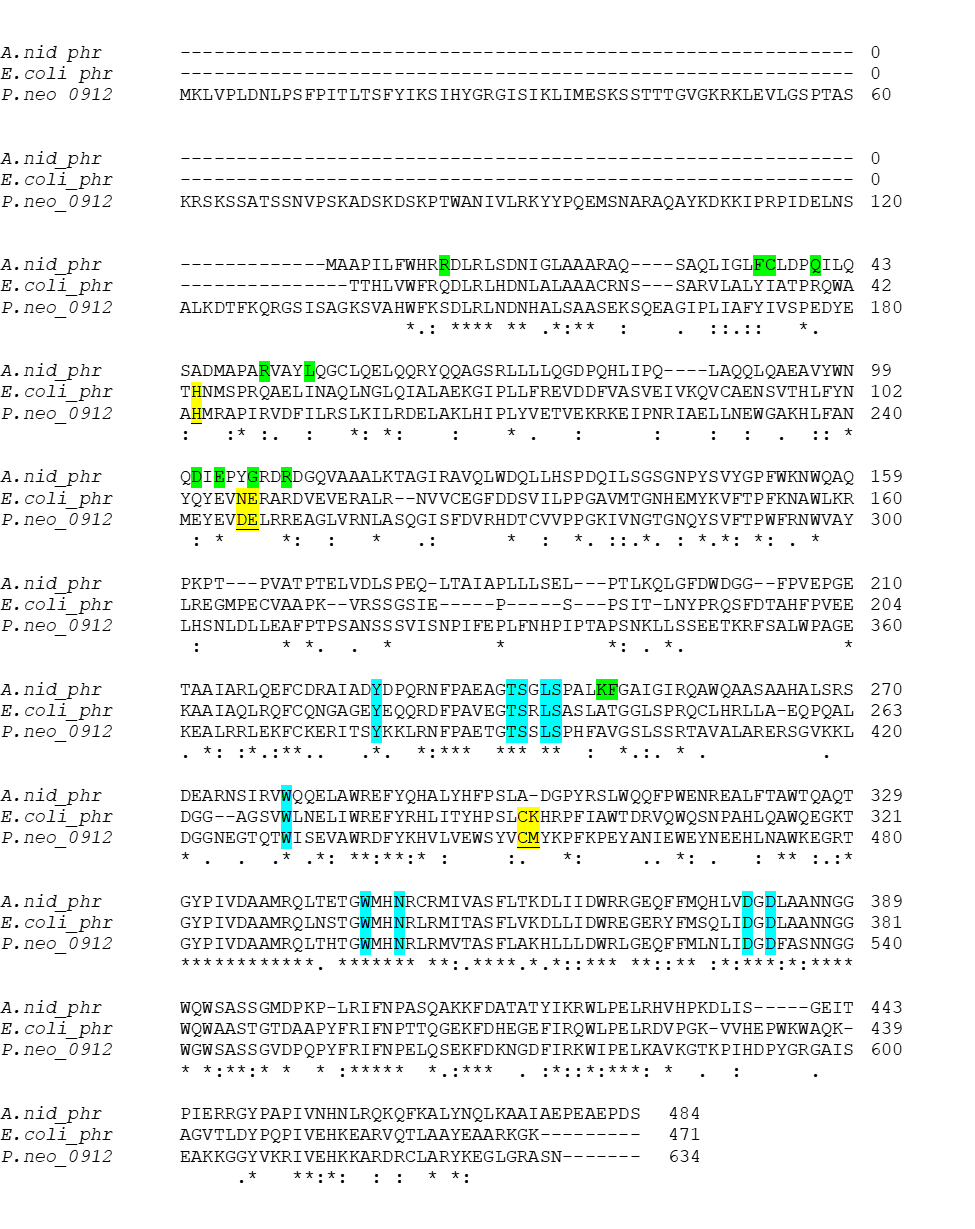


**Fig. S7.** A sequence alignment of *Pseudoidium neolycopersici* (*P. neo_0912*) photolyase with the *Escherichia coli* (*E. coli_phr*) photolyase (PDB id: 1DNP) and *Anacystis nidulans* (*A. nid_phr*) photolyase (PDB id: 1QNF) showing the conservation of residues involved in interaction with the FAD (highlighted in cyan) and MTHF (highlighted in yellow) cofactors. All the FAD interacting residues are completely conserved in *E. coli*, *P. neolycopersici* and *A. nidulans* and MTHF interacting residues are conserved with some variations only in *P. neolycopersici*, however the mode of MTHF binding would still be same, as the present residues would form an identical bonding pattern in the predicted structure (see Fig. 4C). In *A. nidulans*, MTHF residues are not conserved as it has 8-HDF (highlighted in green) as a second cofactor.


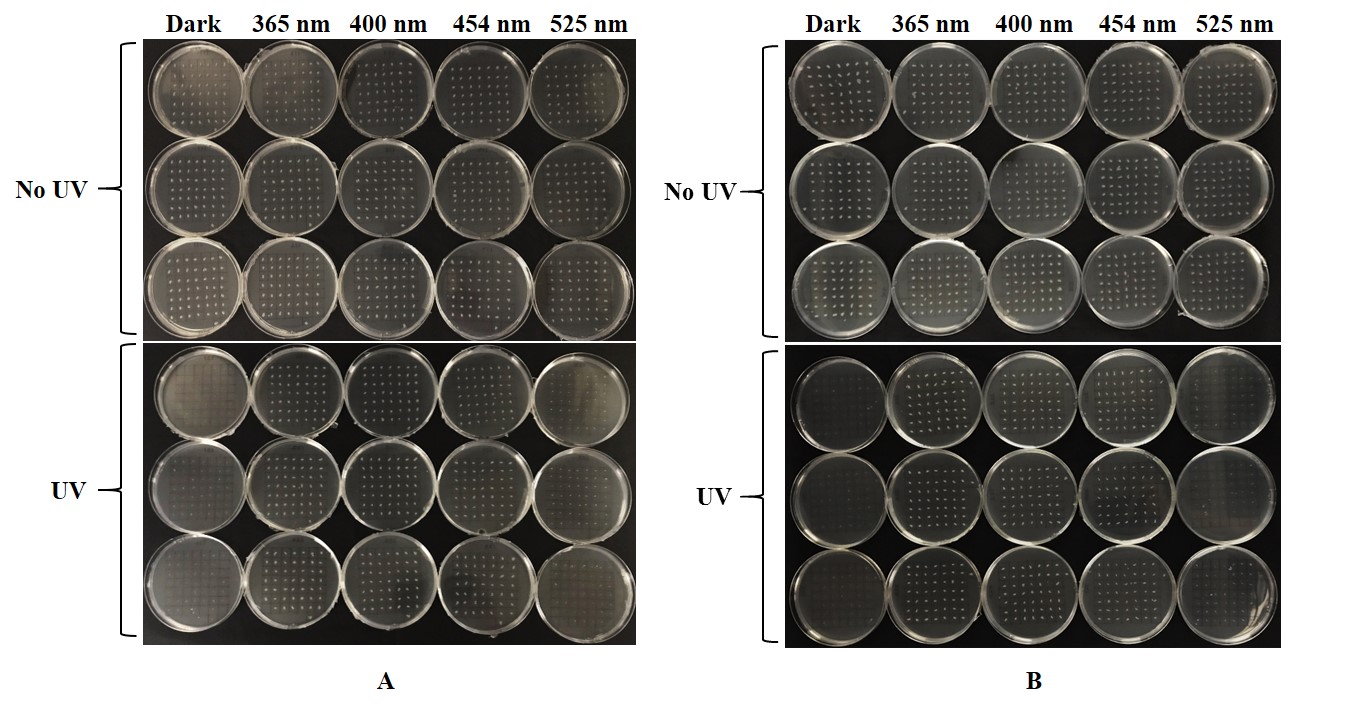


**Fig. S8.** The effect of incubation wavelength immediately after brief UV treatment on recombinant *Escherichia coli* strains. Samples were plated on LB agar (Amp^R^, Kan^R^), exposed to either dark (non-UV) or UV-C (peak 254 nm of 2 ± 0.2 µmol m^-2^ s^-1^ for 10 s). After treatment, samples were immediately incubated in dark, 365 nm, 400 nm, 454 nm and 525 nm of 25 ± 5 µmol m^-2^ s^-1^ for 2 h at 25°C followed by incubation at 37°C for overnight. (A) *E. coli* strain KY1225 containing pQE-30Xa_PN0912 construct (KY1225_ pQE-30Xa_PN0912) with functional photolyase gene from *Pseudoidium neolycopersici* and (B) *E. coli* positive control KY1056 (photolyase-proficient, wild type) strain transformed with empty pQE-30Xa.


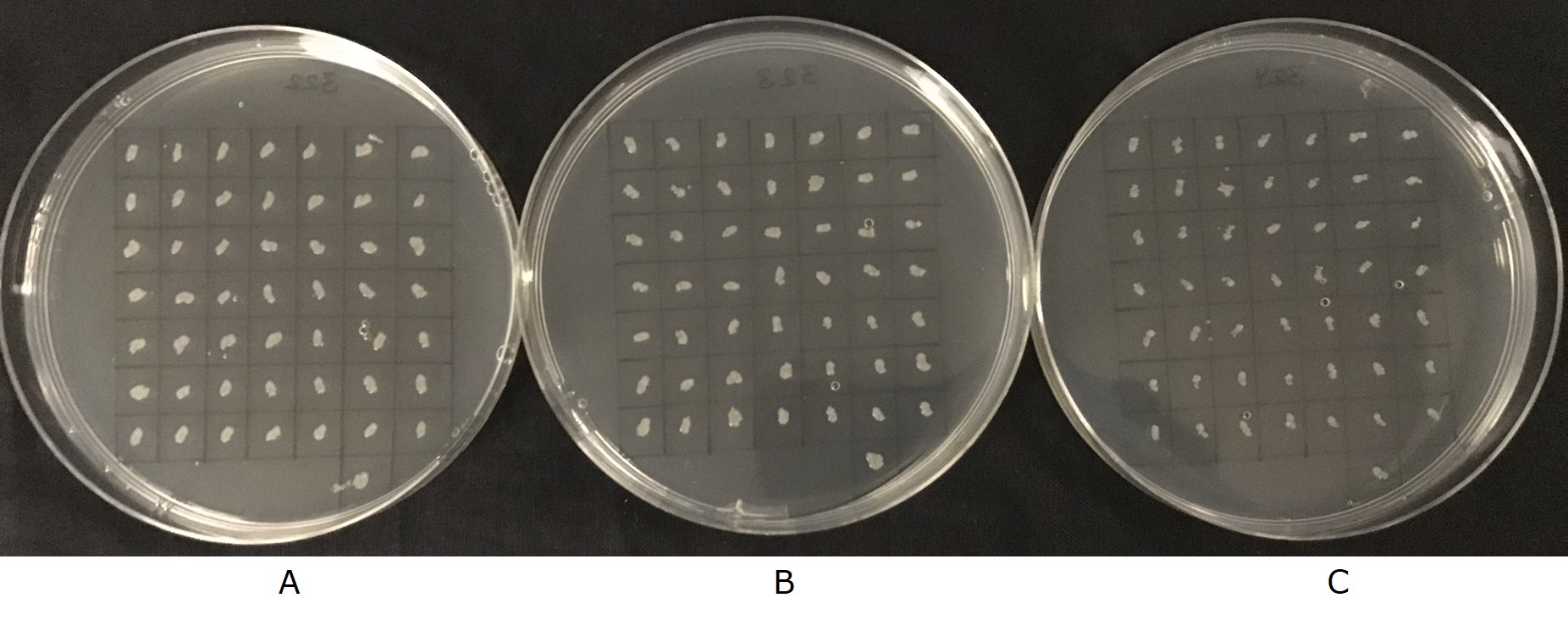


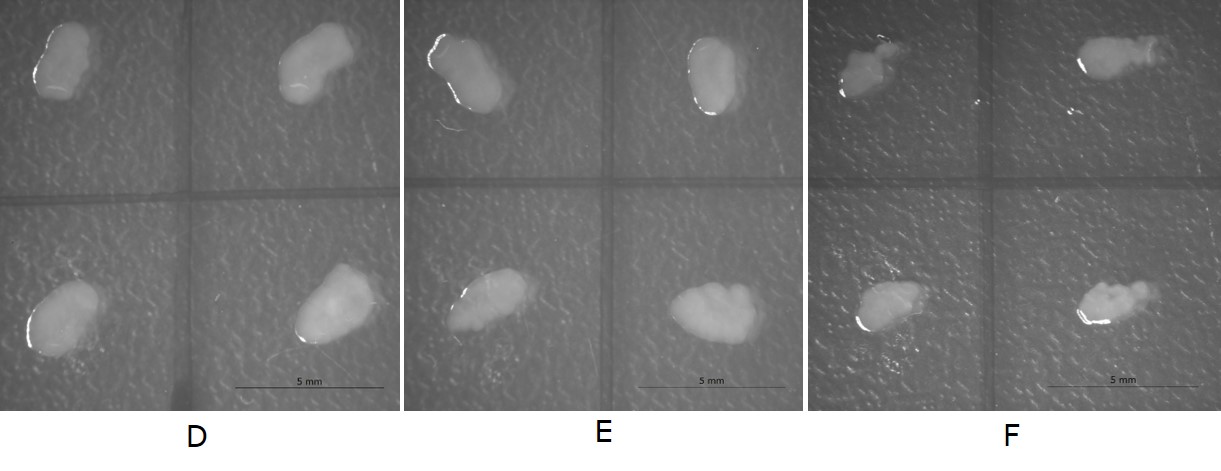


**Fig. S9.** The effect of incubation wavelengths on KY1225_pQE-30Xa_PN0912 construct containing functional photolyase gene from *Pseudoidium neolycopersici*. (A) 365 nm, (B) 400 nm and (C) 454 nm immediately after brief UV treatment on *Escherichia coli*. Samples were plated on LB agar (Amp^R^, Kan^R^), exposed to UV-C, peak 254 nm of 2 ± 0.2 µmol m^-2^ s^-1^ for 10 s. After UV treatment, samples were immediately incubated in 365 nm, 400 nm and 454 nm of 25 ± 5 µmol m^-2^ s^-1^ for 2 h at 25°C followed by incubation at 37°C for overnight. Fig. D, E & F were taken from fig. A, B & C respectively with similar magnification (5 mm, scale bar) in stereomicroscope.

**Supplemental Data 1. Coding sequence information of *Pseudoidium neolycopersici* cryptochrome/ photolyase family (CPF) like genes and respective codon optimized sequences for *Escherichia coli* expression system.**

**>OINE01015670_T110144_CDS**

ATGGGGAAATCGAGAGTCTTGTATTGGTTTAGGACCGACTTACGGCTTCATGACTCTCCAGCTCTAAAAGCTGCATTAGATTTGAATCCTGAAGCGTTCTGGCCTATATGGACGTGGGACCCATATTATGTTTATCAATCAAGAGTTGGAATAAATCGGTGGCAGTTTTTAATTGATTGTCAAAACGAGCTTTCTCGAAGAATATCTCAAATAAATGAAAAATCTAAATTATTTGTCATTAGAGAAGCGCCACAAACTGTTCTGCCTAAACTTTTTAAAGCATGGAAGATAACTCATTTGGTATTTGAGAAAGATGTAGATGCCTATGGTAGGGAAAGAGACGCCGCTATTATACAAGCGGCTGAGGCAGCTGATGTCAAAGTTCTTATGTGCCATGGTCGAACACTCTGGGATAGTGATGAATTGGTCAAAAAGAACAAAAATCAACCCACCATGACTATAAGTCAAGTCCAAGCTGCAGGTCCCAAAGTGGGTAAGATTCCACGGCCTCTACCAACACCTACGCAAATACCTAATCCAGGTGATTTAACAATTCCATTTGAACATAAGAAACCCGAACCAATTCCTGATCTTTGTGATTCACTAAGAGAAAGCAGTGATAGGTCTTTTGGTAGTATTTCTGGGCCACAGGGCGACTTTGCAGTGCCCACAATGAGTGAACTTGGCTTTCCAGTAGCAACAACTCCCCATCGTGGTGGAGAGTATATTGCACTGAAATTGCTTGATAAAATCATTGCCAATGAGCATTATACAGCAACATTTGAGAAACCAAATACAGCACCTACCGCATTTGAGCCCCAGTCAACAACAATGCTTTCCCCACATCTTCATTTTGGTAGTCTCGGTATAAGAGAATTTTACTGGCGTGTTCAGGATGTTGTTGAAAAATTTGGTGGAAAAGCGTCTAAACCACCCGTCTCACTGACCGGCCAGCTTCTCTTCCGGGACATGTATTTTGGCGCTCAAGCTGCACTTTCTCATTCCTTCAACCAAACCGTTTTCAATTCCCACTGCCGCTTTATTCCTTGGCACCTTCAAAGTAAATTTGATCCAAAGACAAAGCGTGTCACAGGCGAATATCATATTGATTCACCACAGGCCGAAGAATGGTTTCAGCGCTGGAAGTGTGGTCGCACTGGCTTTCCTTGGATTGATGCTCTAATGCGACAGTTGCGACAAGAAGGATGGATCCATCACTTAGGTCGCCATGCAGTTGCATGTTTTCTGACGCGAGGCGGATGTTACATTTCTTGGGAGCGCGGGGCCGAAGTATTCAAGGAATTGCTCTTAGATCACGAACCTGCTTGTAATGCAGGTAACTGGCAATGGCTATCTTGTACAGCTTTTTTCACTCAGTTCTTCAGATGCTATAGCCCTATTGCGTTTCCGCAAAAATATGACAAGAATGGCGACTTCGTTCGTCGATATGTCCCGGAACTTAAGATGCTGGATAAAAAGTACATTTATGAGCCATGGAAAGCGCCACTTCTAGATCTGAAGAAAGCGGGAGTAAAATTGCAAAATAATTGGAATGATCTCACTGAAGGGGTATATGTAAAGCCAATGTTTGACTTCAATGAGCAAAGAAATGTCTGTATGGAGGGTATGAAGAAAGCCTACAAAGTCGGTTTATATGGCGACAATCCATGCGTCATAGATGGAACATGGGAAAATCTTTTTGGATCTGGTGGGGAGAAAAAAAAAGAGAAAGATGCTTTGCAGGATGATGAATGTGACGGATTAAGGCCTAAAGACGGGTCGGAGGATAAATCAATACATAAGAAAACAAAAAATAGTGCAGGTAAAAAAAGAAGTCAAGCAACACTTGAGGAACATTTAAAGCAAAAGAAGTCAAAGACGTGTGATCACAAATAA

**>OINE01015670_T110144_CDS_Codon optimized**

ATGGGCAAGAGCCGTGTGCTGTACTGGTTTCGTACCGACCTGCGTCTGCATGATAGCCCGGCGCTGAAAGCGGCGCTGGACCTGAACCCGGAAGCGTTCTGGCCGATCTGGACCTGGGATCCGTACTATGTGTATCAGAGCCGTGTTGGCATCAACCGTTGGCAGTTTCTGATTGACTGCCAAAACGAGCTGAGCCGTCGTATCAGCCAGATTAACGAGAAGAGCAAGCTGTTCGTGATCCGTGAGGCGCCGCAAACCGTTCTGCCGAAACTGTTCAAGGCGTGGAAAATTACCCACCTGGTGTTTGAAAAGGACGTTGATGCGTACGGTCGTGAGCGTGACGCGGCGATCATTCAAGCGGCGGAAGCGGCGGATGTGAAGGTTCTGATGTGCCACGGCCGTACCCTGTGGGACAGCGATGAGCTGGTTAAGAAAAACAAAAACCAGCCGACCATGACCATCAGCCAGGTGCAAGCGGCGGGTCCGAAGGTTGGCAAAATTCCGCGTCCGCTGCCGACCCCGACCCAAATCCCGAATCCGGGTGACCTGACCATTCCGTTTGAACACAAGAAACCGGAGCCGATCCCGGACCTGTGCGATAGCCTGCGTGAAAGCAGCGACCGTAGCTTCGGTAGCATTAGCGGTCCGCAGGGCGATTTTGCGGTGCCGACCATGAGCGAACTGGGTTTTCCGGTTGCGACCACCCCGCACCGTGGTGGCGAGTACATTGCGCTGAAGCTGCTGGATAAAATCATTGCGAACGAACACTATACCGCGACCTTTGAGAAACCGAACACCGCGCCGACCGCGTTTGAGCCGCAAAGCACCACCATGCTGAGCCCGCACCTGCACTTCGGTAGCCTGGGCATTCGTGAATTTTACTGGCGTGTGCAGGACGTGGTTGAGAAATTCGGTGGCAAGGCGAGCAAACCGCCGGTTAGCCTGACCGGTCAACTGCTGTTCCGTGATATGTATTTTGGTGCGCAGGCGGCGCTGAGCCACAGCTTCAACCAAACCGTGTTTAACAGCCACTGCCGTTTCATCCCGTGGCACCTGCAGAGCAAGTTTGACCCGAAAACCAAGCGTGTTACCGGTGAATACCACATTGATAGCCCGCAGGCGGAGGAATGGTTTCAACGTTGGAAATGCGGTCGTACCGGCTTCCCGTGGATCGACGCGCTGATGCGTCAGCTGCGTCAAGAGGGTTGGATTCATCATCTGGGCCGTCACGCGGTGGCGTGCTTTCTGACCCGTGGTGGCTGCTATATCAGCTGGGAGCGTGGTGCGGAAGTTTTCAAAGAGCTGCTGCTGGATCACGAACCGGCGTGCAACGCGGGTAACTGGCAGTGGCTGAGCTGCACCGCGTTCTTTACCCAATTCTTTCGTTGCTACAGCCCGATCGCGTTTCCGCAGAAGTATGACAAAAACGGTGATTTCGTGCGTCGTTACGTTCCGGAACTGAAAATGCTGGACAAGAAATACATTTATGAGCCGTGGAAGGCGCCGCTGCTGGATCTGAAGAAAGCGGGTGTGAAACTGCAGAACAACTGGAACGACCTGACCGAAGGCGTGTATGTTAAGCCGATGTTCGATTTTAACGAACAACGTAACGTTTGCATGGAGGGCATGAAGAAAGCGTACAAAGTGGGTCTGTATGGCGACAACCCGTGCGTTATCGATGGTACCTGGGAGAACCTGTTCGGCAGCGGTGGCGAAAAGAAAAAGGAGAAGGACGCGCTGCAGGACGATGAATGCGATGGTCTGCGTCCGAAAGACGGCAGCGAGGATAAGGCATTCACAAAAAGACCAAAAACAGCGCGGGTAAAAAGCGTAGCCAGGCGACCCTGGAGGAACACCTGAAGCAGAAGAAGAGCAAAACCTGCGACCACAAGTAA

**Amino Acid Sequence**

**>OINE01015670_T110144_AA**

MGKSRVLYWFRTDLRLHDSPALKAALDLNPEAFWPIWTWDPYYVYQSRVGINRWQFLIDCQNELSRRISQINEKSKLFVIREAPQTVLPKLFKAWKITHLVFEKDVDAYGRERDAAIIQAAEAADVKVLMCHGRTLWDSDELVKKNKNQPTMTISQVQAAGPKVGKIPRPLPTPTQIPNPGDLTIPFEHKKPEPIPDLCDSLRESSDRSFGSISGPQGDFAVPTMSELGFPVATTPHRGGEYIALKLLDKIIANEHYTATFEKPNTAPTAFEPQSTTMLSPHLHFGSLGIREFYWRVQDVVEKFGGKASKPPVSLTGQLLFRDMYFGAQAALSHSFNQTVFNSHCRFIPWHLQSKFDPKTKRVTGEYHIDSPQAEEWFQRWKCGRTGFPWIDALMRQLRQEGWIHHLGRHAVACFLTRGGCYISWERGAEVFKELLLDHEPACNAGNWQWLSCTAFFTQFFRCYSPIAFPQKYDKNGDFVRRYVPELKMLDKKYIYEPWKAPLLDLKKAGVKLQNNWNDLTEGVYVKPMFDFNEQRNVCMEGMKKAYKVGLYGDNPCVIDGTWENLFGSGGEKKKEKDALQDDECDGLRPKDGSEDKSIHKKTKNSAGKKRSQATLEEHLKQKKSKTCDHK

**>OINE01000912_T103440_CDS**

ATGAAATTAGTGCCCCTTGATAATTTACCATCTTTCCCCATTACTCTTACTTCTTTCTACATTAAATCCATACATTATGGTCGTGGTATCTCTATAAAGCTAATTATGGAGTCAAAGTCTTCTACTACAACAGGAGTAGGAAAGAGGAAATTGGAGGTACTTGGCTCTCCAACAGCCAGTAAAAGGTCAAAATCTTCAGCAACCTCCTCAAATGTACCCAGCAAGGCTGATTCTAAAGACTCTAAACCTACATGGGCTAATATTGTTCTAAGAAAATACTACCCTCAAGAGATGAGTAACGCGCGTGCTCAAGCCTACAAAGATAAAAAAATACCTCGCCCTATTGATGAGCTAAATTCTGCGCTTAAAGACACCTTTAAGCAGCGTGGATCAATAAGTGCTGGCAAGTCAGTGGCACATTGGTTCAAAAGCGATTTGAGATTAAATGATAATCATGCATTGTCTGCTGCAAGTGAGAAGTCTCAGGAAGCTGGTATACCTTTGATAGCATTTTATATTGTTAGTCCAGAGGATTATGAAGCTCATATGAGAGCGCCAATAAGAGTAGACTTTATACTCCGTTCCCTAAAAATTCTACGAGATGAATTAGCGAAATTGCATATACCTTTATATGTTGAGACTGTGGAGAAGAGAAAAGAAATTCCAAATCGGATTGCTGAGTTGTTGAATGAATGGGGTGCGAAGCATTTATTTGCCAATATGGAATATGAAGTTGATGAGCTTCGTCGAGAAGCTGGATTGGTTCGAAATCTTGCTTCTCAAGGCATTTCTTTTGATGTACGGCATGATACTTGTGTTGTGCCGCCCGGGAAGATTGTTAATGGTACGGGTAATCAGTATTCAGTGTTCACGCCTTGGTTTCGCAATTGGGTCGCGTACTTGCACTCTAATCTTGATCTCCTAGAGGCTTTTCCTACCCCATCTGCAAATTCATCATCTGTTATTTCAAATCCTATATTTGAGCCTTTGTTTAATCACCCAATTCCTACAGCTCCATCAAATAAGCTACTCTCCTCCGAAGAAACAAAGCGTTTCTCTGCCCTCTGGCCGGCGGGCGAAAAAGAGGCTCTTAGAAGACTTGAGAAATTCTGCAAAGAGCGAATTACTTCTTATAAAAAATTGAGAAATTTCCCTGCTGAAACAGGGACATCAAGCTTAAGTCCGCATTTCGCAGTTGGCTCATTAAGCTCCCGGACTGCTGTCGCTCTGGCGAGAGAGCGGAGTGGAGTTAAAAAATTAGATGGAGGCAATGAAGGAACCCAAACTTGGATATCGGAAGTTGCTTGGAGAGATTTTTATAAACATGTGCTTGTTGAATGGTCTTATGTTTGTATGTACAAACCATTCAAGCCCGAGTACGCTAATATTGAATGGGAATATAACGAGGAACACTTAAATGCATGGAAAGAAGGCCGTACTGGCTACCCAATAGTAGATGCAGCAATGCGACAGCTTACTCATACTGGCTGGATGCATAATCGTCTACGCATGGTTACAGCTTCTTTCCTTGCCAAACATCTTCTTCTCGACTGGCGCCTTGGTGAGCAATTTTTCATGCTAAACCTTATTGATGGTGATTTTGCCTCAAACAATGGTGGATGGGGTTGGAGTGCTAGCTCAGGTGTAGATCCACAACCTTACTTTCGCATCTTTAATCCAGAACTTCAATCTGAAAAATTTGACAAGAATGGTGATTTTATTCGAAAATGGATTCCCGAATTGAAAGCCGTGAAGGGCACTAAACCTATTCATGATCCCTACGGTAGAGGTGCCATTTCTGAAGCCAAGAAAGGCGGATACGTAAAAAGAATTGTAGAACACAAAAAAGCACGAGATCGGTGTTTGGCACGATACAAAGAAGGCCTTGGAAGAGCGAGTAATTGA

**>OINE01000912_T103440_CDS_Codon optimized**

ATGAAACTGGTGCCGCTGGATAACCTGCCGAGCTTCCCGATCACCCTGACCAGCTTTTACATCAAGAGCATTCAC

TATGGTCGTGGCATCAGCATTAAACTGATTATGGAGAGCAAGAGCAGCACCACCACCGGTGTGGGCAAGCGTAAA

CTGGAAGTTCTGGGTAGCCCGACCGCGAGCAAGCGTAGCAAAAGCAGCGCGACCAGCAGCAACGTGCCGAGCAAA

GCGGACAGCAAGGATAGCAAACCGACCTGGGCGAACATCGTTCTGCGTAAATACTATCCGCAGGAGATGAGCAAC

GCGCGTGCGCAAGCGTACAAGGACAAGAAAATCCCGCGTCCGATTGATGAACTGAACAGCGCGCTGAAAGACACC

TTCAAGCAGCGTGGTAGCATCAGCGCGGGCAAAAGCGTTGCGCACTGGTTTAAGAGCGACCTGCGTCTGAACGAT

AACCATGCGCTGAGCGCGGCGAGCGAGAAGAGCCAAGAAGCGGGCATCCCGCTGATTGCGTTCTACATTGTGAGC

CCGGAGGACTATGAAGCGCACATGCGTGCGCCGATCCGTGTTGATTTTATTCTGCGTAGCCTGAAAATCCTGCGT

GACGAGCTGGCGAAGCTGCACATTCCGCTGTACGTGGAGACCGTTGAAAAGCGTAAAGAAATCCCGAACCGTATT

GCGGAGCTGCTGAACGAATGGGGTGCGAAACACCTGTTCGCGAACATGGAGTATGAAGTGGATGAGCTGCGTCGT

GAAGCGGGTCTGGTTCGTAACCTGGCGAGCCAGGGCATCAGCTTTGACGTGCGTCACGATACCTGCGTGGTTCCG

CCGGGCAAGATTGTGAACGGTACCGGCAACCAATACAGCGTTTTCACCCCGTGGTTTCGTAACTGGGTGGCGTAT

CTGCACAGCAACCTGGACCTGCTGGAAGCGTTTCCGACCCCGAGCGCGAACAGCAGCAGCGTTATCAGCAACCCG

ATTTTCGAACCGCTGTTTAACCACCCGATCCCGACCGCGCCGAGCAACAAACTGCTGAGCAGCGAGGAAACCAAA

CGTTTCAGCGCGCTGTGGCCGGCGGGCGAGAAAGAAGCGCTGCGTCGTCTGGAGAAGTTTTGCAAAGAACGTATT

ACCAGCTACAAGAAACTGCGTAACTTTCCGGCGGAGACCGGTACCAGCAGCCTGAGCCCGCACTTTGCGGTTGGT

AGCCTGAGCAGCCGTACCGCGGTGGCGCTGGCGCGTGAACGTAGCGGTGTTAAGAAACTGGATGGTGGCAACGAG

GGCACCCAGACCTGGATCAGCGAAGTGGCGTGGCGTGACTTCTACAAACACGTGCTGGTTGAGTGGAGCTACGTT

TGCATGTATAAGCCGTTTAAACCGGAATACGCGAACATCGAGTGGGAATATAACGAGGAACACCTGAACGCGTGG

AAAGAGGGTCGTACCGGCTATCCGATTGTGGATGCGGCGATGCGTCAGCTGACCCACACCGGTTGGATGCACAAC

CGTCTGCGTATGGTTACCGCGAGCTTCCTGGCGAAACACCTGCTGCTGGACTGGCGTCTGGGTGAACAATTCTTT

ATGCTGAACCTGATCGACGGCGATTTTGCGAGCAACAACGGTGGCTGGGGTTGGAGCGCGAGCAGCGGCGTGGAC

CCGCAGCCGTACTTCCGTATTTTTAACCCGGAGCTGCAAAGCGAAAAGTTCGACAAAAACGGTGATTTTATCCGT

AAGTGGATTCCGGAGCTGAAGGCGGTTAAAGGCACCAAGCCGATCCACGATCCGTACGGTCGTGGCGCGATTAGC

GAGGCGAAGAAAGGTGGCTATGTGAAACGTATCGTTGAACACAAGAAAGCGCGTGACCGTTGCCTGGCGCGTTAT

AAGGAAGGTCTGGGCCGTGCGAGCAACTAA

**Amino Acid Sequence**

**>OINE01000912_T103440_AA**

MKLVPLDNLPSFPITLTSFYIKSIHYGRGISIKLIMESKSSTTTGVGKRKLEVLGSPTASKRSKSSATSSNVPSKADSKDSKPTWANIVLRKYYPQEMSNARAQAYKDKKIPRPIDELNSALKDTFKQRGSISAGKSVAHWFKSDLRLNDNHALSAASEKSQEAGIPLIAFYIVSPEDYEAHMRAPIRVDFILRSLKILRDELAKLHIPLYVETVEKRKEIPNRIAELLNEWGAKHLFANMEYEVDELRREAGLVRNLASQGISFDVRHDTCVVPPGKIVNGTGNQYSVFTPWFRNWVAYLHSNLDLLEAFPTPSANSSSVISNPIFEPLFNHPIPTAPSNKLLSSEETKRFSALWPAGEKEALRRLEKFCKERITSYKKLRNFPAETGTSSLSPHFAVGSLSSRTAVALARERSGVKKLDGGNEGTQTWISEVAWRDFYKHVLVEWSYVCMYKPFKPEYANIEWEYNEEHLNAWKEGRTGYPIVDAAMRQLTHTGWMHNRLRMVTASFLAKHLLLDWRLGEQFFMLNLIDGDFASNNGGWGWSASSGVDPQPYFRIFNPELQSEKFDKNGDFIRKWIPELKAVKGTKPIHDPYGRGAISEAKKGGYVKRIVEHKKARDRCLARYKEGLGRASN

**>OINE01005061_T102555_CDS**

ATGATTCTTGAGCTAGTACCCCTTATGTTCCGCAATATAAGTTGTAGTTCACGTCTTCTTCCAAAGCTACTTAAATTTGAATCAAACCTCTTAAGAAAAGACATACATACCACTGCCTCTAAGATGACGGAGATAAAAAGGCTGCTAATCTACGTAATGCGCCGAGATTTGCGAGTAGCAGATAATCCTATACTATATGAATTGGCAACGAACAGTAAAAAGCATGGATTTACCCACATGCTTCCACTTTATGTTTTTTCAGCCCAGCAAATTGAAGTAAGTGGCTTCGTTGATGGGCAAGAGAAGTGTCCATTTCCTGAGGCAAGGAGTCGTATAGCTGGATTTTGGCGCTGTGGCTATCACCGCGCTAAGTTTATTTCCGAGAGTCTGGATGATGTGAAAGAAAGGTTGGAAGAGATTGGAAGTAGCCTATGTATTCGGGTAGGCATGATTGGCAATGTAATTGAGGATATGATAGCCAAATATGCTAGAGAGGACTTTAAGGTTGCGGCAGTATGGATGGTGGGGGAAAGTGCTTCAGAGGAGATAAGTGAAGAAGCTGCTGTGAAAAATGCGTGTAAGGCTGCCAAGGTGGGGTTTAAAGTCTGGGCAGATGAGAAATATCTCATAGATGACCGTGAACTGCCATTTGATAAAATTAAAGATCTTCCTGATGTCTTCACAAGTTTTAGAAAAAGCGTCGAGCCACTTCGCACCATTCCACGAGCTTCACTTCCCACACCCTCAAAAGGATCACTTCCCGCATACCCCAATATTATTCCTTCGCAGCAGCCACCCTTTAGCGTCCCTCTTTCCTTTGTAGAAATCCAAAAAGCTCTTCTTAAGCCACTTGAAGCTTTAATCCTTATTGATGATCCACCAAAATTTCCACTTGGTGCGTCCTCGAGTTTCCCACTAAAAGGTGGTTGCTCCCATGCAACGAAGCGCCTCCGACATCTTTTATTATCCTCAAGTATAAGCAACTATAAAGAAACTCGGAATGGACTTTTAGGAGTTGACTATTCAACAAAGCTCTCTGCCTATCTTGCTCTTGGCTGTATCACATCACGACAAATTCACCACGCTCTCCTCTCGCTTGAGAATGGAACAGATTCGTCTCTTTCATCCGTCCATGGATATGGAGCCGGTGAAAATGATGGGACTAAAGCGACTCGCTTTGAACTACTGTGGCGTGACTATATGCGACTCTGCACTCGAAAATTTGGAATTAAGTTATTCCGTCTTTCTGGTTTCCGCGACCAAAAAGAATATAGGCCAATGTGGAACTTGCCTTTAAAGCCTTTGCCTGGGAGCTCGATAGCTCAAGTTCAAGAAATGATTAAACGTTTCCTAAATGGAACTACGGGCATGGGGCTTATTGATGCTGCTCAGCGAGAACTTTACCACACAGGCTTTACGTCCAACCGTACGCGTCAGAATGTTGCCTCGTTTCTAGCGAAACATCTCAAGATTGACTGGCGCATCGGTGCTGAGTGGTATGAATGTATGCTTGTAGATCATGATGTGTCTTCCAACTGGGGTAATTGGCAATATGTATCCGGTGTTGGTAATGATCCTCGAGGTGAAGACAGAGTATTCAATCCGGTAAAGCAGGCTCTCGACTATGATCCTTACGCTGAATACGTAAAGACTTGGTGTCCAGAACTACGGGCTGAAGGATTAGAGATCAGTGAGATATTTCAACCCTGGACTATTCCAGAGACAAAGAGGGAGGCTTTGGGATTAAAAGGATTAATTGGTGTTGAAAAACCTTTGCGGAAAATTCAATTTGGTAGCTACGGCAGTAGAGGTGGTCATTCTCAACACCATAAAACACAACTGAATACGAATCAACAAGGGCGAAACTCAGGCCGAGGTAAAGCACCAAAAAATGACTATGGTGGTCGTGGCTATGGGAGTTCTAAGGGATACGCAACGACTTCGAAAGGAAAAGGCGCTGCATCAAATAAATGGGTTGATACGTAA

**>OINE01005061_T102555_CDS_Codon optimized**

ATGATTCTGGAACTGGTTCCGCTGATGTTTCGTAACATCAGCTGCAGCAGCCGTCTGCTGCCGAAGCTGCTGAAA

TTCGAAAGCAACCTGCTGCGTAAGGACATCCACACCACCGCGAGCAAGATGACCGAGATTAAACGTCTGCTGATC

TATGTTATGCGTCGTGACCTGCGTGTGGCGGATAACCCGATTCTGTACGAACTGGCGACCAACAGCAAGAAACAC

GGTTTTACCCACATGCTGCCGCTGTATGTTTTCAGCGCGCAGCAAATCGAAGTTAGCGGTTTTGTGGATGGCCAG

GAAAAATGCCCGTTCCCGGAGGCGCGTAGCCGTATTGCGGGTTTTTGGCGTTGCGGCTACCACCGTGCGAAGTTC

ATCAGCGAAAGCCTGGACGATGTGAAAGAGCGTCTGGAGGAAATTGGTAGCAGCCTGTGCATCCGTGTTGGCATG

ATCGGTAACGTGATTGAAGACATGATCGCGAAGTATGCGCGTGAGGATTTTAAAGTGGCGGCGGTTTGGATGGTG

GGTGAAAGCGCGAGCGAGGAAATTAGCGAGGAAGCGGCGGTGAAGAACGCGTGCAAGGCGGCGAAAGTTGGCTTT

AAAGTGTGGGCGGACGAAAAATACCTGATTGACGATCGTGAGCTGCCGTTCGATAAGATCAAAGACCTGCCGGAT

GTTTTCACCAGCTTTCGTAAGAGCGTGGAGCCGCTGCGTACCATCCCGCGTGCGAGCCTGCCGACCCCGAGCAAA

GGTAGCCTGCCGGCGTACCCGAACATCATTCCGAGCCAGCAACCGCCGTTTAGCGTTCCGCTGAGCTTCGTGGAA

ATCCAGAAGGCGCTGCTGAAACCGCTGGAGGCGCTGATCCTGATTGACGATCCGCCGAAATTTCCGCTGGGTGCG

AGCAGCAGCTTTCCGCTGAAGGGTGGCTGCAGCCACGCGACCAAACGTCTGCGTCACCTGCTGCTGAGCAGCAGC

ATCAGCAACTACAAGGAAACCCGTAACGGCCTGCTGGGTGTTGACTATAGCACCAAACTGAGCGCGTACCTGGCG

CTGGGTTGCATTACCAGCCGTCAGATCCACCACGCGCTGCTGAGCCTGGAGAACGGCACCGATAGCAGCCTGAGC

AGCGTGCACGGTTATGGCGCGGGTGAAAACGACGGTACCAAGGCGACCCGTTTTGAGCTGCTGTGGCGTGATTAC

ATGCGTCTGTGCACCCGTAAGTTCGGTATTAAACTGTTTCGTCTGAGCGGCTTCCGTGACCAAAAGGAGTATCGT

CCGATGTGGAACCTGCCGCTGAAACCGCTGCCGGGTAGCAGCATTGCGCAGGTTCAAGAAATGATCAAACGTTTC

CTGAACGGCACCACCGGTATGGGTCTGATTGATGCGGCGCAGCGTGAGCTGTACCACACCGGTTTTACCAGCAAC

CGTACCCGTCAAAACGTGGCGAGCTTCCTGGCGAAGCACCTGAAAATTGATTGGCGTATCGGCGCGGAGTGGTAT

GAATGTATGCTGGTTGACCACGATGTGAGCAGCAACTGGGGTAACTGGCAGTACGTTAGCGGCGTGGGTAACGAC

CCGCGTGGCGAAGATCGTGTTTTCAACCCGGTGAAGCAAGCGCTGGACTACGATCCGTATGCGGAATACGTTAAA

ACCTGGTGCCCGGAGCTGCGTGCGGAGGGTCTGGAAATCAGCGAGATTTTTCAGCCGTGGACCATCCCGGAAACC

AAGCGTGAGGCGCTGGGCCTGAAAGGTCTGATTGGCGTGGAGAAGCCGCTGCGTAAAATCCAATTCGGTAGCTAT

GGTAGCCGTGGTGGCCACAGCCAGCACCACAAGACCCAACTGAACACCAACCAGCAAGGTCGTAACAGCGGCCGT

GGCAAGGCGCCGAAAAACGACTATGGTGGCCGTGGCTACGGTAGCAGCAAGGGCTACGCGACCACCAGCAAGGGC

AAGGGTGCGGCGAGCAACAAATGGGTTGATACCTAA

**Amino Acid Sequence**

**>OINE01005061_T102555_AA**

MILELVPLMFRNISCSSRLLPKLLKFESNLLRKDIHTTASKMTEIKRLLIYVMRRDLRVADNPILYELATNSKKHGFTHMLPLYVFSAQQIEVSGFVDGQEKCPFPEARSRIAGFWRCGYHRAKFISESLDDVKERLEEIGSSLCIRVGMIGNVIEDMIAKYAREDFKVAAVWMVGESASEEISEEAAVKNACKAAKVGFKVWADEKYLIDDRELPFDKIKDLPDVFTSFRKSVEPLRTIPRASLPTPSKGSLPAYPNIIPSQQPPFSVPLSFVEIQKALLKPLEALILIDDPPKFPLGASSSFPLKGGCSHATKRLRHLLLSSSISNYKETRNGLLGVDYSTKLSAYLALGCITSRQIHHALLSLENGTDSSLSSVHGYGAGENDGTKATRFELLWRDYMRLCTRKFGIKLFRLSGFRDQKEYRPMWNLPLKPLPGSSIAQVQEMIKRFLNGTTGMGLIDAAQRELYHTGFTSNRTRQNVASFLAKHLKIDWRIGAEWYECMLVDHDVSSNWGNWQYVSGVGNDPRGEDRVFNPVKQALDYDPYAEYVKTWCPELRAEGLEISEIFQPWTIPETKREALGLKGLIGVEKPLRKIQFGSYGSRGGHSQHHKTQLNTNQQGRNSGRGKAPKNDYGGRGYGSSKGYATTSKGKGAASNKWVDT

**Full- length amino acid sequence of the *Pseudoidium neolycopersici* photolyase protein produced by GenScript**

**>OINE01000912_T103440**

MKLVPLDNLPSFPITLTSFYIKSIHYGRGISIKLIMESKSSTTTGVGKRKLEVLGSPTASKRSKSSATSSNVPSKADSKDSKPTWANIVLRKYYPQEMSNARAQAYKDKKIPRPIDELNSALKDTFKQRGSISAGKSVAHWFKSDLRLNDNHALSAASEKSQEAGIPLIAFYIVSPEDYEAHMRAPIRVDFILRSLKILRDELAKLHIPLYVETVEKRKEIPNRIAELLNEWGAKHLFANMEYEVDELRREAGLVRNLASQGISFDVRHDTCVVPPGKIVNGTGNQYSVFTPWFRNWVAYLHSNLDLLEAFPTPSANSSSVISNPIFEPLFNHPIPTAPSNKLLSSEETKRFSALWPAGEKEALRRLEKFCKERITSYKKLRNFPAETGTSSLSPHFAVGSLSSRTAVALARERSGVKKLDGGNEGTQTWISEVAWRDFYKHVLVEWSYVCMYKPFKPEYANIEWEYNEEHLNAWKEGRTGYPIVDAAMRQLTHTGWMHNRLRMVTASFLAKHLLLDWRLGEQFFMLNLIDGDFASNNGGWGWSASSGVDPQPYFRIFNPELQSEKFDKNGDFIRKWIPELKAVKGTKPIHDPYGRGAISEAKKGGYVKRIVEHKKARDRCLARYKEGLGRASNHHHHHH

**Sequence information of *Pseudoidium neolycopersici* Alpha tubulin**

**>OINE01013217_T107300**

ATGACTAGAGGCGAGATTCTCCACCTTCATATTGGCCAGGGAGGCACTCAGCTTGGAAATAGCGCCTGGGAGCTATACCTTCTCGAACATGGCCTATCCAAAGATGGTTACCCAAATCCTGATGCAAAAGACCTCCATGAATCTGGCGAGCTTGACACCGTCTTTACAGAAACAGGTAGTGGAAAATATGTACCACGATCAATTTTTGTGGATCTTGATCCTTCTCCCATTGATGAGATCCGGTTTTTGATTTTTCATTCTTTTGGTGGTGGGACCGGATCTGGCTTTGGATCTCTACTTCTTGAACGCCTATCTACTGATTATGGTAAGAAATCGAAGCTTGAGTTTGCTGTCTATCCTGCACCAAGAGTATCAACTGCCGTCGTTGAGCCATATAATGCGGTTTTATCAACACACTCAACAATTGAAAATAGTGACTGCACTTTCTTAGTTGACAATGAAGCTGTCTATGATATATGTCATCGTAACCTTGGTATCCCTCGTCCGTCTTTCGAACACTTAAATCGATTAATAGCACAAGTGGTGAGCAGTATTACTTCAAGTTTGCGATTTGAAGGCGCTTTGAACGTTGATCTTAACGAATTTCAAACGAATCTCGTCCCATACCCCCGAATTCATTACCCTTTGATTTCATATGCGCCAGTAATTAGTGCCTCTCGTAGTAGTCACGAAAGCTTTAAAACACATGACTTAACCCTACAATGTTTTGAACCTTACAATCAAATGGTAGTATGCGATCCTCGTGCTGGTAAATATATGGCTGTCGCACTTCTCTATCGCGGGGATGTAATTCCTCGGGACTGCAACGCTGCTGTTGTCTCACTCAAAGCTAAACCCTCCTTCAATCTTGTGGAATGGTGCCCTACAGGCTTTAAACTTGGGATCAACTATCAAAAACCTGTGTCAGTACCTTCTTCACCCGATGATGGTGCCCTTGCCTCAGTTGACCGTTCTGTTTCTATGCTTAGCAATACCACTGCCATTGCTGAGGCATGGTCTCGTCTTGACCATAAATTCGATCTTATGTATAATAAGCGTGCATTTGTCCATTGGTATGTTGGTGAGGGTATGGAGGAAGGCGAATTTAGTGAAGCACGAGAAGACCTTGCAGCTCTTGAAAAGGATTATGAAGAAGTTGCTGCTGATTCATATGATCCAGAAGATGGTGAAGCTGAGTATTAA

**Supplemental Data 2. Amino acid sequences of cryptochrome/photolyase family (CPF)- like genes (137 members) used in Phylogenetic analysis, retrieved from NCBI.**

1.

>Anacystis_nidulans_phr

MAAPILFWHRRDLRLSDNIGLAAARAQSAQLIGLFCLDPQILQSADMAPARVAYLQGCLQELQQRYQQAGSRLLLLQGDPQHLIPQLAQQLQAEAVYWNQDIEPYGRDRDGQVAAALKTAGIRAVQLWDQLLHSPDQILSGSGNPYSVYGPFWKNWQAQPKPTPVATPTELVDLSPEQLTAIAPLLLSELPTLKQLGFDWDGGFPVEPGETAAIARLQEFCDRAIADYDPQRNFPAEAGTSGLSPALKFGAIGIRQAWQAASAAHALSRSDEARNSIRVWQQELAWREFYQHALYHFPSLADGPYRSLWQQFPWENREALFTAWTQAQTGYPIVDAAMRQLTETGWMHNRCRMIVASFLTKDLIIDWRRGEQFFMQHLVDGDLAANNGGWQWSASSGMDPKPLRIFNPASQAKKFDATATYIKRWLPELRHVHPKDLISGEITPIERRGYPAPIVNHNLRQKQFKALYNQLKAAIAEPEAEPDS

2.

>gi|161138112|gb|ABX58027.1|_cryptochrome_1a_Triticum_aestivum

MSASPSMSGGAGERTRTRTVVWFRRDLRVEDNPALAAAARTAGEVVPAYVWAPKEDGPYYPGRVSRWWLSQSLKHLDASLRRLGATRLVTRRSTDTVAALLELVRSTGATHLFFNHLYDPLSLVRDHRVKQVLGAEGITVQSFNSDLLYEPWEVLDDHGCPFTMFTPFWNTCLCMVDPPAPMLPPKRINSGELSRCCSSDDLIFEDESERGSNALLARAWSPGWQNADKAFTAFINGPLIDYSVNRKKADSANTSLLSPYLHFGELSVRKVFHQIRMKQLTWSNESNGDGEEGCSLFLRSIGLREYSRRLAFNHPCSHEKPLLAHLRFFPWVVNEVYFKVWRQGRTGYPLVDAGMRELWATGWLHDRIRVVVSSFFVKVLQLPWRWGMKYFWDTLLDADLESDALGWQYISGSLPDGRELDRIDNPQFEGYKFDPYGEYVRRWLPELARLPTEWIHHPWDAPESVLRAAGIELGSNYPLPIVELDEAKSRLQDALSEMWELEAASRAEIENGMEEGLGDSSDEPPIAFPQELQHMEVDRATIHTPATAGRRRADQMVPSITSSLVRAETETELSAAFESEVTRPEVPSQVHFQPQTRMEVRDEGVSDGTAARYNGVQQQQQYTLHRHRVQGGIAPSTSEASSSWTGREGGVVPVWSPPAASGHSDPYAADETDISSRSYLDRHPQQSHRLMNWNQLSQSS

3.

>gi|161138116|gb|ABX58029.1|_cryptochrome_2_Triticum_aestivum

MGGSERTVVWFRRDLRIDDNPALAAAARDGAVLPVFIWCPAEEGRFYTGRCSRWWLKESLAHLARSLQALGCPLVLIRAQTTLAALLQCVDSIGATRVVYIHLYDPISLVRDDKIKNELLGLGISMQSFNGDLLYEPWEVYDENGLPFTTFKKYWEKCMKLHIDISPSLAPWRLVPVSGIENICSSSIDNLGLESSKDEESSNALLSRAWSPGWRNAEKTLEDFVSHGLLDYSKDRMKVAGTTTSLSSPYLHYGEVSVRKIYQLVRVQQIKWENEGKSGAGESDNLFLLSIGLREYSRYLCFNFPFTRERSLLGNLKHYPWRADEDRFKSWRQGMTGYPLVDAGMRELWATGWTHNRIRVIVSSFAVKFLQIPWTWGMKYFWDVLLDADIESDILGWQYISGSLPDGHELGRLDNPEVQGQKYDPDGEYVRTWIPELARMPGEWIHHPWDAPSSILEVAGVELGFNYPMPIVELHTARECLDDAISTMWQLDTAEKLAELDGEVVEDNLSHIKSFDVPKVVLKELSPHCDRKVPTDDGRNLELQPKELKGTNKQTICVDVIKASKMEDTGSIANSPISRKRSSSGSVFNVPSYSSSVEVHSQNQHPGGYLVGSSKYIQQKAERNCVGKAEDDDSADSGTNTSRASKRPAA

4.

>gi|528295567|emb|CCU77936.1|_deoxyribodipyrimidine_photolyase_Blumeria_graminis_f._sp._hordei_DH14

MSKFGRQLVRKILIFDQIVSKRSFLRTSGSSLQIPWTIKMASSNFPSTTTRKRKIEQPESSSSSKKPKVAVSSVNESLTTPDPKHALSAAHGIVLRKYYPHEMNNARAQAYKDNTLTRPISILNDALSETHKLRESTKAGKAVVHWFKSDLRVNDNHALSAASEKAREAGVPLLALFILSPQDLEAHLTAPIRVDFLLRSLKLLRDDLAALHIPLHIKTIEKRKEIPTLIAALLAEWEVKHMFANIEYEVDELRRDAEIVREISKQQIAFDLRHDSCIVQPGKLLSGTGNAYSVYTPWYRAWMAHVHSNPDLLETFPAPTPNPSSITSDSTYKSLFDCPIPKPPENKRLSIEETKRFSALWPAGEREASDRLEKFCKERITGYKAKRNFPAETGTSSLSPHFALGTLSSRTAVRAARESSGSKKMDGGNEGVQTWISEVAWRDFYRHVLVAFPYVCMNKPFKPEYSKIEWEYNDDHFNAWKEGRTGYPIVDAAMRQLAYTGWMHNRLRMVVASFLTKHLLLDWRLGEQYFMLNLIDGDFASNNGGWGWSAGSGVDPQPYFRIFNPELQSEKFDKEGHFIRKWVPELKGVRGSKPIHDPYARGAASEAKSGGYIPRIVNHKEARERCLKRYKEGLGKVTS

5.

>gi|16128683|ref|NP_415236.1|_deoxyribodipyrimidine_photolyase_(photoreactivation)_Escherichia_coli_str._K-12_substr._MG1655

MTTHLVWFRQDLRLHDNLALAAACRNSSARVLALYIATPRQWATHNMSPRQAELINAQLNGLQIALAEKGIPLLFREVDDFVASVEIVKQVCAENSVTHLFYNYQYEVNERARDVEVERALRNVVCEGFDDSVILPPGAVMTGNHEMYKVFTPFKNAWLKRLREGMPECVAAPKVRSSGSIEPSPSITLNYPRQSFDTAHFPVEEKAAIAQLRQFCQNGAGEYEQQRDFPAVEGTSRLSASLATGGLSPRQCLHRLLAEQPQALDGGAGSVWLNELIWREFYRHLITYHPSLCKHRPFIAWTDRVQWQSNPAHLQAWQEGKTGYPIVDAAMRQLNSTGWMHNRLRMITASFLVKDLLIDWREGERYFMSQLIDGDLAANNGGWQWAASTGTDAAPYFRIFNPTTQGEKFDHEGEFIRQWLPELRDVPGKVVHEPWKWAQKAGVTLDYPQPIVEHKEARVQTLAAYEAARKGK

6.

>gi|521771249|gb|EPQ63205.1|_DNA_photolyase_Blumeria_graminis_f._sp._tritici_96224

MSNFRRQLVRNIIIFDQISYKLSFLQTSGSSLRIPWTIKMASSNLPSVTTRKRKVEQPESLSSSKKLKVAVSSVNESLTTPDPKYALSAAHGIVLRKYYPHEMNNARAQAYKDNALTRPISILNAALAETHKLRENTKVGKAVVHWFKSDLRVKDNHALSAASEKAREADVPLLALFILSPQDLEAHLTAPIRVDFLLRSLKLLRDDLAVLNIPLHIMTIEKRKEIPTLIAALLAEWEVKHIFANIEYEVDELRRDAEIVRGISKQEIAFDLRHDSCIVQPGKLLSGTGNAYSVYTPWYRAWMAHVHSNPDLLETFPAPTPNPSSITSDSTYTSLFNCPIPKPPENKRLSFDETKRFSALWPAGEREASDRLEKFCKERITGYKAKRNFPAETGTSSLSPHFALGTLSSRTAVRAARESSGSKKIDGGNEGVQTWISEVAWRDFYRHVLVAFPYVCMNKPFKPEYSKIEWEYNDDHFNAWKEGRTGYPIVDAAMRQLAYTGWMHNRLRMVVASFLTKHLLLDWRLGEQYFMLNLIDGDFASNNGGWGWSAGSGVDPQPYFRIFNPELQSEKFDKEGHFIRKWVPELKGVRGSKPIHDPYGRGAASEAKSGGYIPRIVNHKEARERCLQRYKEGLGKVTS

7.

>gi|730183381|gb|KHJ34441.1|_putative_deoxyribodipyrimidine_photo-lyase_Erysiphe_necator

MSNARAQDYKDKRIPRPIDELNSALKDTFKQRGVISPGRSVVHWFKSDLRLKDNHALSAASEKAQKSGVPLITFYIVSPEDYEAHLRAPIRVDFILRSLKTLQDDLAKLHIPLYVETVEKRREVPLRIAQLLNNWGTKHLFTNMEYEVDELRREAAMVRDLASQDIAFDVRHDTCVVPPGKVVNGTGNQYSVFTPWFRNWVTYLHSNLDLLETFPAPIANSPSINSDPNLKSLFNQLIPVAPLNKQLSKEEEKRFTALWPAGEKEAFARLEKFCKERITSYKKLRNFPAETGTSSLSPHFAVGSLSSRTAVAVARERNGLKKLDGGDEGIQTWISEVAWRDFYKHVLVEWSYVCMYKPFKPEYTNIQWECNEEHLNAWKEGRTGYPIVDAAMRQLTHTGWMHNRLRMVTASFLSKHLLLDWRLGEQFFMLNLIDGDFASNNGGWGWSASSGVDPQPYFRIFNPELQSEKFDKNGDFIRKWIPELKAVKGAKAIHDPFGRGAISEAKKGGYVKRIVAHKEARDRCLARYKEGLMKPSS

8.

>gi|6324962|ref|NP_015031.1|_deoxyribodipyrimidine_photolyase_PHR1_Saccharomyces_cerevisiae_S288C

MKRTVISSSNAYASKRSRLDIEHDFEQYHSLNKKYYPRPITRTGANQFNNKSRAKPMEIVEKLQKKQKTSFENVSTVMHWFRNDLRLYDNVGLYKSVALFQQLRQKNAKAKLYAVYVINEDDWRAHMDSGWKLMFIMGALKNLQQSLAELHIPLLLWEFHTPKSTLSNSKEFVEFFKEKCMNVSSGTGTIITANIEYQTDELYRDIRLLENEDHRLQLKYYHDSCIVAPGLITTDRGTNYSVFTPWYKKWVLYVNNYKKSTSEICHLHIIEPLKYNETFELKPFQYSLPDEFLQYIPKSKWCLPDVSEEAALSRLKDFLGTKSSKYNNEKDMLYLGGTSGLSVYITTGRISTRLIVNQAFQSCNGQIMSKALKDNSSTQNFIKEVAWRDFYRHCMCNWPYTSMGMPYRLDTLDIKWENNPVAFEKWCTGNTGIPIVDAIMRKLLYTGYINNRSRMITASFLSKNLLIDWRWGERWFMKHLIDGDSSSNVGGWGFCSSTGIDAQPYFRVFNMDIQAKKYDPQMIFVKQWVPELISSENKRPENYPKPLVDLKHSRERALKVYKDAM

9.

>gi|1435243662|ref|NP_066940.3|_cryptochrome-2_isoform_1_Homo_sapiens

MAATVATAAAVAPAPAPGTDSASSVHWFRKGLRLHDNPALLAAVRGARCVRCVYILDPWFAASSSVGINRWRFLLQSLEDLDTSLRKLNSRLFVVRGQPADVFPRLFKEWGVTRLTFEYDSEPFGKERDAAIMKMAKEAGVEVVTENSHTLYDLDRIIELNGQKPPLTYKRFQAIISRMELPKKPVGLVTSQQMESCRAEIQENHDETYGVPSLEELGFPTEGLGPAVWQGGETEALARLDKHLERKAWVANYERPRMNANSLLASPTGLSPYLRFGCLSCRLFYYRLWDLYKKVKRNSTPPLSLFGQLLWREFFYTAATNNPRFDRMEGNPICIQIPWDRNPEALAKWAEGKTGFPWIDAIMTQLRQEGWIHHLARHAVACFLTRGDLWVSWESGVRVFDELLLDADFSVNAGSWMWLSCSAFFQQFFHCYCPVGFGRRTDPSGDYIRRYLPKLKAFPSRYIYEPWNAPESIQKAAKCIIGVDYPRPIVNHAETSRLNIERMKQIYQQLSRYRGLCLLASVPSCVEDLSHPVAEPSSSQAGSMSSAGPRPLPSGPASPKRKLEAAEEPPGEELSKRARVAELPTPELPSKDA

10.

>gi|15219720|ref|NP_171935.1|_cryptochrome_2_Arabidopsis_thaliana

MKMDKKTIVWFRRDLRIEDNPALAAAAHEGSVFPVFIWCPEEEGQFYPGRASRWWMKQSLAHLSQSLKALGSDLTLIKTHNTISAILDCIRVTGATKVVFNHLYDPVSLVRDHTVKEKLVERGISVQSYNGDLLYEPWEIYCEKGKPFTSFNSYWKKCLDMSIESVMLPPPWRLMPITAAAEAIWACSIEELGLENEAEKPSNALLTRAWSPGWSNADKLLNEFIEKQLIDYAKNSKKVVGNSTSLLSPYLHFGEISVRHVFQCARMKQIIWARDKNSEGEESADLFLRGIGLREYSRYICFNFPFTHEQSLLSHLRFFPWDADVDKFKAWRQGRTGYPLVDAGMRELWATGWMHNRIRVIVSSFAVKFLLLPWKWGMKYFWDTLLDADLECDILGWQYISGSIPDGHELDRLDNPALQGAKYDPEGEYIRQWLPELARLPTEWIHHPWDAPLTVLKASGVELGTNYAKPIVDIDTARELLAKAISRTREAQIMIGAAPDEIVADSFEALGANTIKEPGLCPSVSSNDQQVPSAVRYNGSKRVKPEEEEERDMKKSRGFDERELFSTAESSSSSSVFFVSQSCSLASEGKNLEGIQDSSDQITTSLGKNGCK

11.

>gi|15641816|ref|NP_231448.1|_deoxyribodipyrimidine_photolyase_Vibrio_cholerae_O1_biovar_El_Tor_str._N16961

MSKKIGLYWFTNDLRVNDNPLLEQASQQVDRLICLYCYPSITPFLARYAQQTQWGEAKKRFLNQTLADLDHSLSTLGQKLWVTPLLPYQALRHLLTQVEITDIYVDAVAGSDERQAIARIHQDFSSVHIHQQALHSLLSEPQLPFALEALPSTFTQFRKQVETISLSAPMGYPHVLPPIEQGWQLPLMDIVTEPNHSAFVGGEQAGLTHCQNYFSSLLPSRYKETRNGLDGMDYSTKFSPWLALGAVSPKTIYAMLQRYEAVHGANDSTYWIFFELLWREYFYWYARRYGAKLFRFSGIGEKKPLTSFYAQRFLQWKHGETPFPIVNACMRQLNQTGYMSNRGRQLVASCLVHELGLDWRYGAAYFETQLVDYDVGSNWGNWQYLAGVGADPRGSRQFNLEKQAHTYDPKGEFVAKWCGTACDKLNALENLALDSVDMVDWPIAASAYLLIHHPQNKESSS

12.

>gi|15600828|ref|NP_232458.1|_deoxyribodipyrimidine_photolyase_Vibrio_cholerae_O1_biovar_El_Tor_str._N16961

MRLVWFRRDLRSFDNTALTAALNSGDPVAAMYIATPEQWHQHHLAPIQADLIWRRLAELQQELAALNVPLFYQQVADFQAAAVAVSQLAKTLNATQVLANRDYELDEQQRDQLAQQLLSEQGIIWSAFDDKCVLPPGSVRTKQGEFFKVFTPFKRAWLTLFQPPVIGKNRPVALWNVPSALAELVWHPEQAFDYPRIDSTPWAADFETVRAQLRDFCRERVQDYHQARDFPAREGTSSLSPYLAIGVLSARQCVARLYHESSMGELSEGAQVWLSELIWREFYQHLVAIEPNLSKSRDFVEWGARLEWWNDNEKFQLWCEGKTGYPIVDAAMRQLNQTGWMHNRLRMIVASFLTKDLHIDWRWGERYFMSRLIDGDYAANNGGWQWCASTGCDGQPYFRIFNPVSQGEKFDPNGDFIRRWVPELRSVSSAYIHQPWTYPAVNSVLYPARLVDHKQEREVTLRLYKTAKG

13.

>gi|15888554|ref|NP_354235.1|_DNA_photolyase_Agrobacterium_fabrum_str._C58

MSLKTAPVIVWFRKDLRLSDNLALLAAVEHGGPVIPVYIREKSAGPLGGAQEWWLHHSLAALSSSLEKAGGRLVLASGDAERILRDLISETGADTVVWNRRYDPTGMATDKALKQKLRDDGLTVRSFSGQLLHEPSRLQTKSGGPYRVYTPFWRALEGSDEPHAPADPPKSLTAPKVWPKSEKLSNWKLLPTKPDWAKDFSDIWTPGETGALDKLDDFIDGALKGYEEGRDFPAKPATSLLSPHLAAGEISPAAVWHATKGLSRHIASNDISRFRKEIVWREFCYHLLFHFPELGEKNWNDSFDAFSWRDDEKSFKAWTRGMTGYPIVDAGMRQLWQHGTMHNRVRMIVASFLIKHLLIDWRKGEKWFRDTLVDADPASNAANWQWVAGSGADASPFFRIFNPILQGEKFDGDGDYVRRFVPELEKLERKYIHKPFEAPKDALKKAGVELGKTYPLPIVDHGKARERALAAYAAVKKTT

14.

>gi|16125677|ref|NP_420241.1|_deoxyribodipyrimidine_photolyase_Caulobacter_vibrioides_CB15

MQVRNDSGDSKANLDAVIVWFRKDLRIADNPALRHAAQSGRPVIPLYILDETPGIRPMGGASLWWLDKSLKSLAASLETLGTKLVLRKGVAAEVLDQLIAQSGARSVVWNRLYDKPSTDRDAAIKAALRDRGVDCQSFNAGLLNEPWTVKNGSDQPYKVFTPYWRAAREHLTDVAVTAAPGHLVAPARFPASESLASWNLHPTKPDWSKGFDLWTPGEAGAHARLDAFLKGPIKGYGDQRDIPGVEATSKLSPHLHFGEIGPRQVWLATRSAADQGDIPLAEADKFLSEIGWREFNHSILYNWPHMPSANFKPEFDGFPWVKDEGALEAWKRGQTGYPIVDAGMRELWTTGFMHNRVRMIVASFLIKHLMIDWREGEAWFWDTLLDADLANNVGNWQWTAGSGADAAPYFRIFNPIAQGEKFDPKGDYVRRWVPELRNVSDDVIHKPWTKPLHLPAGAKRLYSRPIVDHAMARARALEAYHGL

15.

>gi|16764079|ref|NP_459694.1|_deoxyribodipyrimidine_photolyase_Salmonella_enterica_subsp._enterica_serovar_Typhimurium_str._LT2

MPTHLVWFRRDLRLQDNLALAAACRDASARVLALYISTPAQWQAHDMAPRQAAFISAQLNALQTALAEKGIPLLFHEVADFNASIETVKNVCRQHDVSHLFYNYQYEFNERQRDAAVEKTLPSVICEGFDDSVILAPGAVMTGNHEMYKVFTPFKNAWLKRLKEDIPPCVPAPKIRVSGALSTPLTPVSLNYPQQAFDAALFPVEENAVIAQLRQFCAQGADEYALRRDFPAVDGTSRLSASLATGGLSPRQCLHRLLAEQPQALDGGPGSVWLNELIWREFYRHLMTWYPALCKHQPFIRWTKRVAWQENPHYFQAWQKGETGYPIVDAAMRQLNATGWMHNRLRMITASFLVKDLLIDWRLGERYFMSQLIDGDLAANNGGWQWAASTGTDAAPYFRIFNPTTQGERFDRDGEFIRQWLPALRDIPGKAIHEPWRWAEKAGVVLDYPRPIVEHKQARIATLSAYEAARKGA

16.

>gi|24586396|ref|NP_523653.2|_photorepair_isoform_A_Drosophila_melanogaster

MFTLASYWRESFKIVLPLQAMKRTKAQKAGPSKKAAKNEKASSEPKSDQESSDEEASTSKALLVSKPDYQNFEQFLTHLEHQRVCTAANIQEFSFRKKRVRVLSKTEDVKESSLGGVVYWMSRDGRVQDNWALLFAQRLALKLELPLTVVFCLVPKFLNATIRHYKFMMGGLQEVEQQCRALDIPFHLLMGSAVEKLPQFVKSKDIGAVVCDFAPLRLPRQWVEDVGKALPKSVPLVQVDAHNVVPLWVASDKQEYAARTIRNKINSKLGEYLSVFPPVVRHPHGTGCKNVNTVDWSAAYASLQCDMEVDEVQWAKPGYKAACQQLYEFCSRRLRHFNDKRNDPTADALSGLSPWLHFGHISAQRCALEVQRFRGQHKASADAFCEEAIVRRELADNFCFYNEHYDSLKGLSSWAYQTLDAHRKDKRDPCYSLEELEKSLTYDDLWNSAQLQLVREGKMHGFLRMYWAKKILEWTATPEHALEYAILLNDKYSLDGRDPNGYVGCMWSIGGVHDMGWKERAIFGKVRYMNYQGCRRKFDVNAFVMRYGGKVHKKK

17.

>gi|18400841|ref|NP_566520.1|_DNA_photolyase_family_protein_Arabidopsis_thaliana

MQRFCVCSPSSYRLNPITSMATGSGSLIWFRKGLRVHDNPALEYASKGSEFMYPVFVIDPHYMESDPSAFSPGSSRAGVNRIRFLLESLKDLDSSLKKLGSRLLVFKGEPGEVLVRCLQEWKVKRLCFEYDTDPYYQALDVKVKDYASSTGVEVFSPVSHTLFNPAHIIEKNGGKPPLSYQSFLKVAGEPSCAKSELVMSYSSLPPIGDIGNLGISEVPSLEELGYKDDEQADWTPFRGGESEALKRLTKSISDKAWVANFEKPKGDPSAFLKPATTVMSPYLKFGCLSSRYFYQCLQNIYKDVKKHTSPPVSLLGQLLWREFFYTTAFGTPNFDKMKGNRICKQIPWNEDHAMLAAWRDGKTGYPWIDAIMVQLLKWGWMHHLARHCVACFLTRGDLFIHWEQGRDVFERLLIDSDWAINNGNWMWLSCSSFFYQFNRIYSPISFGKKYDPDGKYIRHFLPVLKDMPKQYIYEPWTAPLSVQTKANCIVGKDYPKPMVLHDSASKECKRKMGEAYALNKKMDGKVDEENLRDLRRKLQKDEHEESKIRNQRPKLK

18.

>gi|18413170|ref|NP_567341.1|_cryptochrome_1_Arabidopsis_thaliana

MSGSVSGCGSGGCSIVWFRRDLRVEDNPALAAAVRAGPVIALFVWAPEEEGHYHPGRVSRWWLKNSLAQLDSSLRSLGTCLITKRSTDSVASLLDVVKSTGASQIFFNHLYDPLSLVRDHRAKDVLTAQGIAVRSFNADLLYEPWEVTDELGRPFSMFAAFWERCLSMPYDPESPLLPPKKIISGDVSKCVADPLVFEDDSEKGSNALLARAWSPGWSNGDKALTTFINGPLLEYSKNRRKADSATTSFLSPHLHFGEVSVRKVFHLVRIKQVAWANEGNEAGEESVNLFLKSIGLREYSRYISFNHPYSHERPLLGHLKFFPWAVDENYFKAWRQGRTGYPLVDAGMRELWATGWLHDRIRVVVSSFFVKVLQLPWRWGMKYFWDTLLDADLESDALGWQYITGTLPDSREFDRIDNPQFEGYKFDPNGEYVRRWLPELSRLPTDWIHHPWNAPESVLQAAGIELGSNYPLPIVGLDEAKARLHEALSQMWQLEAASRAAIENGSEEGLGDSAEVEEAPIEFPRDITMEETEPTRLNPNRRYEDQMVPSITSSLIRPEEDEESSLNLRNSVGDSRAEVPRNMVNTNQAQQRRAEPASNQVTAMIPEFNIRIVAESTEDSTAESSSSGRRERSGGIVPEWSPGYSEQFPSEENGIGGGSTTSSYLQNHHEILNWRRLSQTG

19.

>gi|1063729030|ref|NP_568461.3|_cryptochrome_3_Arabidopsis_thaliana

MAASSLSLSSPLSNPLRRFTLHHLHLSKKPLSSSSLFLCSAAKMNDHIHRVPALTEEEIDSVAIKTFERYALPSSSSVKRKGKGVTILWFRNDLRVLDNDALYKAWSSSDTILPVYCLDPRLFHTTHFFNFPKTGALRGGFLMECLVDLRKNLMKRGLNLLIRSGKPEEILPSLAKDFGARTVFAHKETCSEEVDVERLVNQGLKRVGNSTKLELIWGSTMYHKDDLPFDVFDLPDVYTQFRKSVEAKCSIRSSTRIPLSLGPTPSVDDWGDVPTLEKLGVEPQEVTRGMRFVGGESAGVGRVFEYFWKKDLLKVYKETRNGMLGPDYSTKFSPWLAFGCISPRFIYEEVQRYEKERVANNSTYWVLFELIWRDYFRFLSIKCGNSLFHLGGPRNVQGKWSQDQKLFESWRDAKTGYPLIDANMKELSTTGFMSNRGRQIVCSFLVRDMGLDWRMGAEWFETCLLDYDPCSNYGNWTYGAGVGNDPREDRYFSIPKQAQNYDPEGEYVAFWLQQLRRLPKEKRHWPGRLMYMDTVVPLKHGNGPMAGGSKSGGGFRGSHSGRRSRHNGP

20.

>gi|40254688|ref|NP_571861.2|_cryptochrome-2_Danio_rerio

MVVNSVHWFRKGLRLHDNPALQEALNGADTVRCVYILDPWFAGSANVGVNRWRFLLESLEDLDTSLRKLNSRLFVVRGQPTDVFPRLFKEWNVTRLTFEYDSEPYGKERDAAIIKMAQEYGVETVVRNTHTLYNPDRIIEMNNHSPPLTFKRFQAIVNRLELPRKPLPTITQEQMARCRTQISDNHDEHYGVPSLEELGFRTQGDSLHVWKGGETEALERLNKHLDRKAWVANFERPRISGQSLFPSPTGLSPYLRFGCLSCRVFYYNLRDLFMKLRRRSSPPLSLFGQLLWREFFYTAGTNNPNFDHMEGNPICVQIPWDHNPEALAKWAEGRTGFPWIDAIMTQLRQEGWIHHLARHAVACFLTRGDLWISWESGMKVFEELLLDADWSVNAGSWMWLSCSAFFQQFFHCYCPVGFGRRTDPSGDYIRRYIPKLKDYPNRYIYEPWNAPESVQKAANCIVGVDYPKPMINHAESSRLNIERMKQVYQQLSHYRGLSLLASVPTIQEEAEPPMSDDSQASSSSTGQASSPPHLSITAPSTPPLSESSSPNSSPTASTSVPHTQRKRVRPSETPSKQKTKVKHTSQARGLELKMDDKQ

21.

>gi|18858473|ref|NP_571863.1|_cryptochrome_circadian_regulator_5_Danio_rerio

MSHNTIHWFRKGLRLHDNPALIAALKDCRHIYPLFLLDPWFPKNTRIGINRWRFLIEALKDLDSSLKKLNSRLFVVRGSPTEVLPKLFKQWKITRLTFEVDTEPYSQSRDKEVMKLAKEYGVEVTPKISHTLYNIDRIIDENNGKTPMTYIRLQSVVKAMGHPKKPIPAPTNEDMRGVSTPLSDDHEEKFGIPTLEDLGLDTSSLGPHLFPGGEQEALRRLDEHMERTNWVCKFEKPKTSPNSLIPSTTVLSPYVRFGCLSARTFWWRLADVYRGKTHSDPPVSLHGQLLWREFFYTTAVGIPNFNKMEGNSACVQVDWDNNPEHLAAWREARTGFPFIDTIMTQLRQEGWIHHLARHAVACFLTRGDLWISWEEGQKVFEELLLDSDWSLNAGNWQWLSASTFFHQYFRVYSPIAFGKKTDKHGDYIKKYLPVLKKFPTEYIYEPWKAPRSVQERAGCIVGKDYPRPIVDHEVVHKKNILRMKAAYAKRSPEDKTINKGEKRKASPSIKEMFQKKAKR

22.

>gi|24585455|ref|NP_724274.1|_(6-4)-photolyase_isoform_A_Drosophila_melanogaster

MDSQRSTLVHWFRKGLRLHDNPALSHIFTAANAAPGKYFVRPIFILDPGILDWMQVGANRWRFLQQTLEDLDNQLRKLNSRLFVVRGKPAEVFPRIFKSWRVEMLTFETDIEPYSVTRDAAVQKLAKAEGVRVETHCSHTIYNPELVIAKNLGKAPITYQKFLGIVEQLKVPKVLGVPEKLKNMPTPPKDEVEQKDSAAYDCPTMKQLVKRPEELGPNKFPGGETEALRRMEESLKDEIWVARFEKPNTAPNSLEPSTTVLSPYLKFGCLSARLFNQKLKEIIKRQPKHSQPPVSLIGQLMWREFYYTVAAAEPNFDRMLGNVYCMQIPWQEHPDHLEAWTHGRTGYPFIDAIMRQLRQEGWIHHLARHAVACFLTRGDLWISWEEGQRVFEQLLLDQDWALNAGNWMWLSASAFFHQYFRVYSPVAFGKKTDPQGHYIRKYVPELSKYPAGCIYEPWKASLVDQRAYGCVLGTDYPHRIVKHEVVHKENIKRMGAAYKVNREVRTGKEEESSFEEKSETSTSGKRKVRRATGSAPKRKR

23.

>gi|24648152|ref|NP_732407.1|_cryptochrome_Drosophila_melanogaster

MATRGANVIWFRHGLRLHDNPALLAALADKDQGIALIPVFIFDGESAGTKNVGYNRMRFLLDSLQDIDDQLQAATDGRGRLLVFEGEPAYIFRRLHEQVRLHRICIEQDCEPIWNERDESIRSLCRELNIDFVEKVSHTLWDPQLVIETNGGIPPLTYQMFLHTVQIIGLPPRPTADARLEDATFVELDPEFCRSLKLFEQLPTPEHFNVYGDNMGFLAKINWRGGETQALLLLDERLKVEQHAFERGFYLPNQALPNIHDSPKSMSAHLRFGCLSVRRFYWSVHDLFKNVQLRACVRGVQMTGGAHITGQLIWREYFYTMSVNNPNYDRMEGNDICLSIPWAKPNENLLQSWRLGQTGFPLIDGAMRQLLAEGWLHHTLRNTVATFLTRGGLWQSWEHGLQHFLKYLLDADWSVCAGNWMWVSSSAFERLLDSSLVTCPVALAKRLDPDGTYIKQYVPELMNVPKEFVHEPWRMSAEQQEQYECLIGVHYPERIIDLSMAVKRNMLAMKSLRNSLITPPPHCROINEEEVRQFFWLADVVV

24.

>gi|30021247|ref|NP_832878.1|_deoxyribodipyrimidine_photolyase_Bacillus_cereus_ATCC_14579

MQNKIIVMFQKDFRLYDNPALFEAVQSGEVLPVYIQDETFSIGSASKWWLHHAVIDVKKQLEALGSTLIIRKGRTEEEILSLIEQLDITAVYWNICYDPDRLQSNQKMKMMLEDKGIICKEFNSHLLLEPWIIKKKDNTEYKVFTPFYNAFQKQVIPKPFSRVQSIKWGNSLPASLSVSELQLLPIIPWTSHMEVIWDPTEEGAYKTFKKFFSSKLVSYSEGRDFPGQNVHSMLAPYLSFGQISVKLMFHYLINKSTERQCSLFEKQVNSFIRQLIWREFSYYLLYHYPFTVYKPLNKSFENFPWDKEEELLRVWQKGKTGYPFIDAGMRELWQTGFMHNRARMAVASFLVKHLLIPWQEGAKWFMDTLLDADIANNTMGWQWVAGSGADASPYFRIFNPITQGEKFDKNGEYIRRWVPELRDIPNKYIHKPWEAPEHILQKSNIKLGDTYPFPIVDHKAARERALCAYKSMKEFV

25.

>gi|30682738|ref|NP_849651.1|_photolyase_1_Arabidopsis_thaliana

MASTVSVQPGRIRILKKGSWQPLDQTVGPVVYWMFRDQRLKDNWALIHAVDLANRTNAPVAVVFNLFDQFLDAKARQLGFMLKGLRQLHHQIDSLQIPFFLLQGDAKETIPNFLTECGASHLVTDFSPLREIRRCKDEVVKRTSDSLAIHEVDAHNVVPMWAASSKLEYSARTIRGKINKLLPDYLIEFPKLEPPKKKWTGMMDKKLVDWDSLIDKVVREGAEVPEIEWCVPGEDAGIEVLMGNKDGFLTKRLKNYSTDRNNPIKPKALSGLSPYLHFGQVSAQRCALEARKVRSTSPQAVDTFLEELIVRRELSDNFCYYQPHYDSLKGAWEWARKSLMDHASDKREHIYSLEQLEKGLTADPLWNASQLEMVYQGKMHGFMRMYWAKKILEWTKGPEEALSISIYLNNKYEIDGRDPSGYVGCMWSICGVHDQGWKERPVFGKIRYMNYAGCKRKFNVDSYISYVKSLVSVTKKKRKAEEQLTRDSVDPKITIV

26.

>gi|45383642|ref|NP_989575.1|_cryptochrome-2_Gallus_gallus

MAAAASPPRGFCRSVHWFRRGLRLHDNPALQAALRGAASLRCIYILDPWFAASSAVGINRWRFLLQSLEDLDNSLRKLNSRLFVVRGQPTDVFPRLFKEWGVTRLTFEYDSEPFGKERDAAIIKLAKEAGVEVVIENSHTLYDLDRIIELNGNKPPLTYKRFQAIISRMELPKKPVSSIVSQQMETCKVDIQENHDDVYGVPSLEELGFPTDGLAPAVWQGGETEALARLDKHLERKAWVANYERPRMNANSLLASPTGLSPYLRFGCLSCRLFYYRLWELYKKVKRNSTPPLSLYGQLLWREFFYTAATNNPKFDRMEGNPICIQIPWDKNPEALAKWAEGKTGFPWIDAIMTQLRQEGWIHHLARHAVACFLTRGDLWISWESGVRVFDELLLDADFSVNAGSWMWLSCSAFFQQFFHCYCPVGFGRRTDPSGDYVKRYLPKLKGFPSRYIYEPWNAPESVQKAAKCIIGVDYPKPMVNHAETSRLNIERMKQIYQQLSRYRGLCLLASVPSCVEDLSGPVTDSAPGQGSSTSTAVRLPQSDQASPKRKHEGAEELCTEELYKRAKVTGLPAPEIPGKSS

27.

>gi|45383636|ref|NP_989576.1|_cryptochrome-1_Gallus_gallus

MGVNAVHWFRKGLRLHDNPALRECIRGADTVRCVYILDPWFAGSSNVGINRWRFLLQCLEDLDANLRKLNSRLFVIRGQPADVFPRLFKEWSIAKLSIEYDSEPFGKERDAAIKKLASEAGVEVIVRISHTLYDLDKIIELNGGQPPLTYKRFQTLISRMEPLEMPVETITPEVMQKCTTPVSDDHDEKYGVPSLEELGFDTDGLPSAVWPGGETEALTRLERHLERKAWVANFERPRMNANSLLASPTGLSPYLRFGCLSCRLFYFKLTDLYKKVKKNSSPPLSLYGQLLWREFFYTAATNNPRFDKMEGNPICVQIPWDKNPEALAKWAEGRTGFPWIDAIMTQLRQEGWIHHLARHAVACFLTRGDLWISWEEGMKVFEELLLDADWSVNAGSWMWLSCSSFFQQFFHCYCPVGFGRRTDPNGDYIRRYLPVLRGFPAKYIYDPWNAPESVQKAAKCVIGVNYPKPMVNHAEASRLNIERMKQIYQQLSRYRGLGLLATVPSNPNGNGNGGLMSFSPGESISGCSSAGGAQLGTGDGQTVGVQTCALGDSHTGGSGVQQQGYCQASSILRYAHGDNQQSHLMQPGRASLGTGISAGKRPNPEEETQSVGPKVQRQSTN

28.

>gi|45387783|ref|NP_991249.1|_cryptochrome_DASH_Danio_rerio

MSASRTVICLLRNDLRLHDNEVFHWAQRNAEHIIPLYCFDPRHYQGTYHYNFPKTGPFRLRFLLDSVKDLRALLKKHGSTLLVRQGKPEDVVCELIKQLGSVSTVAFHEEVASEEKSVEEKLKEICCQNKVRVQTFWGSTLYHRDDLPFSHIGGLPDVYTQFRKAVEAQGRVRPVLSTPEQVKSPPSGLEEGPIPTFDSLGQTEPLDDCRSAFPCRGGETEALARLKHYFWDTNAVATYKETRNGMIGVDFSTKFSPWLALGCISPRYIYEQIKKYEVERTANQSTYWVIFELLWRDYFKFVALKYGNRIFYMNGLQDKHVPWKTDMKMFDAWKEGRTGVPFVDANMRELALTGFMSNRGRQNVASFLTKDLGLDWRLGAEWFEYLLVDHDVCSNYGNWLYSAGIGNDPRENRKFNMIKQGLDYDNNGDYVRQWVPELRGIKGGDVHTPWTLSNSALSHAQVSLNQTYPCPIITAPEWSRHVNNKSSGPSSSKGRKGSSYTARQHKDRGIDFYFSKNKHF

29.

>gi|79313247|ref|NP_001030703.1|_DNA_photolyase_family_protein_Arabidopsis_thaliana

MQRFCVCSPSSYRLNPITSMATGSGSLIWFRKGLRVHDNPALEYASKGSEFMYPVFVIDPHYMESDPSAFSPGSSRAGVNRIRFLLESLKDLDSSLKKLGSRLLVFKGEPGEVLVRCLQEWKVKRLCFEYDTDPYYQALDVKVKDYASSTGVEVFSPVSHTLFNPAHIIEKNGGKPPLSYQSFLKVAGEPSCAKSELVMSYSSLPPIGDIGNLGISEVPSLEELGYKDDEQADWTPFRGGESEALKRLTKSISDKAWVANFEKPKGDPSAFLKPATTVMSPYLKFGCLSSRYFYQCLQNIYKDVKKHTSPPVSLLGQLLWREFFYTTAFGTPNFDKMKGNRICKQIPWNEDHAMLAAWRDGKTGYPWIDAIMVQLLKWGWMHHLARHCVACFLTRGDLFIHWEQGRDVFERLLIDSDWAINNGNWMWLSCSSFFYQALSPFCFSF

30.

>gi|390979651|ref|NP_001070765.2|_cryptochrome_1a_Danio_rerio

MVVNTVHWFRKGLRLHDNPSLRDSILGAHSVRCVYILDPWFAGSSNVGISRWRFLLQCLEDLDASLRKLNSRLFVIRGQPTDVFPRLFKEWNINRLSYEYDSEPFGKERDAAIKKLANEAGVEVIVRISHTLYDLDKIIELNGGQSPLTYKRFQTLISRMEAVETPAETITAEVMGPCTTPLSDDHDEKFGVPSLEELGFDTEGLSSAVWPGGETEALTRLERHLERKAWVANFERPRMNANSLLASPTGLSPYLRFGCLSCRLFYFKLTDLYRKVKKNSSPPLSLYGQLLWREFFYTAATNNPRFDKMEGNPICVQIPWDKNPEALAKWAEGRTGFPWIDAIMTQLRQEGWIHHLARHAVACFLTRGDLWISWEEGMKVFEELLLDADWSVNAGSWMWLSCSSFFQQFFHCYCPVSFGRRTDPNGDYIRRYLPVLRGFPAKYIYDPWNAPESVQKAAKCIIGVHYPMPMVHHAEASRLNIERMKQIYQQLSCYRGLGLLAMVPSNPNGNGENSTSLMGFKTGDMTKEVTTPSGYQMPPTSQGEWHGRTMVYSQGDQQTSSIMTSQGFGNNGSTMCYRQDAQQITGRGLHSSIIQTSGKRHSEESGPTTVSKVQRQCSS

31.

>gi|147901097|ref|NP_001081129.1|_cryptochrome-1_Xenopus_laevis

MGVNAVHWFRKGLRLHDNPALRECIQGADTVRCVYILDPWFAGSSNVGINRWRFLLQCLEDLDANLRKLNSRLFVIRGQPADVFPRLFKEWKITKLSIEYDSEPFGKERDAAIKKLASEAGVEVIVRISHTLYDLDKIIELNGGQPPLTYKRFQTLISKMDPLEIPVETITAEVMEKCTTPVSDDHDEKYGVPSLEELGFDTEGLPSAVWPGGETEALTRLERHLERKAWVANFERPRMNANSLLASTTGLSPYLRFGCLSCRLFYFKLTDLYKKVKKNSSPPLSLYGQLLWREFFYTAATNNPRFDKMDGNPICVQIPWDRNPEALAKWAEGRTGFPWIDAIMTQLRQEGWIHHLARHAVACFLTRGDLWISWEEGMKVFEELLLDADWSVNAGSWMWLSCSSFFQQFFHCYCPVGFGKRTDPNGDYIRRYLPILKGFPPKYIYDPWNAPETVQKAAKCIIGVNYPKPMVNHAEASRLNIERMKQIYQQLSRYRGLGLLASVPSNPNGNGGLMSYSPGESMPGCSNNGGGQMGAIEGSSASNPNPNQGEVLPGTSGLQGYWQGSSILHYSHSDNQQSYLMQARNPLHSVVSSGKRPNPEEETQSVGPKVQRQSTH

32.

>gi|147906624|ref|NP_001081421.1|_6-4_photolyase_Xenopus_laevis

MRHNSIHWFRKGLRLHDNPALLAAMKDCAELHPIFILDPWFPKNMQVSVNRWRFLIDALKDLDENLKKINSRLFVVRGKPAEVFPLLFKKWKVTRLTFEVDIEPYSRQRDAEVEKLAAEHDVQVIQKVSNTLYDIDRIIAENNGKPPLTYVRFQTVLAPLGPPKRPIKAPTLENMKDCHTPWKSSYDEKYGVPTLEELGQDPMKLGPHLYPGGESEALSRLDLHMKRTSWVCNFKKPETEPNSLTPSTTVLSPYVKFGCLSARTFWWKIADIYQGKKHSDPPVSLHGQLLWREFYYTTGAGIPNFNKMEGNPVCVQVDWDNNKEHLEAWSEGRTGYPFIDAIMTQLRTEGWIHHLARHAVACFLTRGDLWISWEEGQKVFEELLLDADWSLNAGNWLWLSASAFFHQFFRVYSPVAFGKKTDKNGDYIKKYLPILKKFPAEYIYEPWKSPRSLQERAGCIIGKDYPKPIVEHNVVSKQNIQRMKAAYARRSGSTEGVDKDSGQNNKKGGKRKVAAGTSVAELFKKK

33.

>gi|147901075|ref|NP_001083936.1|_cryptochrome_circadian_regulator_2_S_homeolog_Xenopus_laevis

MEGKPSVSSVHWFRKGLRLHDNPALLAALRGANSVRCVYILDPWFAASSSGGVNRWRFLLQSLEDLDSSLRKLNSRLFVVRGQPADVFPKLFKEWGVSRLTFEYDSEPFGKERDAVIMKLAKEAGVEVIVENSHTLYDSDRVIELNGHSPPLTYKRFQAIISRMELPRRLAPSVTRQQMEACRAEIKRNHDETYGVPSLEELGFHSENKGPAIWPGGETEALARLDRHLERKAWVANYERPRMSANSLLASPTGLSPYLRFGCLSCRLFYYRLQELYQKVKKNSPPPLSLYGQLLWREFFYTAATNNPKFDQMEGNPICVQIPWDKNPKALAKWTEGKTGFPWIDAIMTQLRQEGWIHHLARHAVACFLTRGDLWNSWECGVKVFDELLLDADFSVNAGSWMWLSCSAFFQQFFHCYCPVGFGRRTDPSGDYVKRYLPVLKAFPSRYIYEPWSAPESVQKEAKCIIGIDYPKPIVNHAEASRMNIERMKQTYQQLSHYRGLCILASVPSSVEDLGGPITDSSHNPAEAAPKQSLCNADSPKRKLEGSEEASHVKVRVRSVPVMRRPENDF

34.

>gi|147902555|ref|NP_001084438.1|_cryptochrome_DASH_Xenopus_laevis

MCVPSRVIICLLRNDLRLHDNEVLHWAHRNADQIVPLYCFDPRHYVGTHYFNFPKTGPHRLKFLLESVRDLRITLKKKGSNLLLRRGKPEEVIEDLVKQLGNVSAVTLHEEATKEETDVESAVKQACTRLGIKYQTFWGSTLYHREDLPFRHISSLPDVYTQFRKAVETQGKVRPTFQMPDKLKPLPSGLEEGSVPSHEDFDQQDPLTDPRTAFPCSGGESQALQRLEHYFWETNLVASYKDTRNGLIGLDYSTKFAPWLALGCVSPRYIYEQIGKYEKERTANQSTYWVIFELLWRDYFRFVALKYGRRIFFLRGLQDKDIPWKRDPKLFDAWKEGRTGVPFVDANMRELAMTGFMSNRGRQNVASFLTKDLGIDWRMGAEWFEYLLVDYDVCSNYGNWLYSAGIGNDPRENRKFNMIKQGLDYDSGGDYIRLWVPELQQIKGGDAHTPWALSNASLAHANLSLGETYPYPIVMAPEWSRHINQKPAGSWEKSARRGKGPSHTPKQHKNRGIDFYFSRNKDV

35.

>gi|212275870|ref|NP_001130580.1|_type_II_CPD_DNA_photolyase_Zea_mays

MPPAIPSLVHPSRVRILHPGGSHIHGPVVYWMLRDQRLADNWALLHAAELAAASTPAAPLAIAFTLFPRPFLLGAHLRQLGFLLRGLRRLAADAHARGLPFFLLEGGPAELPSLVRRLGASALVADFSPLRPVREALDAVVQELLRDAANMAVHQVDAHNVVPVWTASGKLEYSAKTFRSKVNKVINEYLVEYPEVPQWAPWCMEQPKSVDWDALINSIFSEAENVPEINWCEPGESSAMEVLLGSKDGFLTKRIKNYDTGRNDPTKPHALSCLSPYLHFGHISAQRCALEAKKRRHLSPKSVDTFLEELIIRRELADNFCYYQPQYDSLAGAWEWARKTLTDHTGDKREHIYTREQLENAKTSDPLWNASQLEMVHHGKMHGFMRMYWAKKILEWTSQPEEALSIAIYLNDKYHIDGRDPNGYVGCMWSICGLHDQGWKERPVFGKIRYMNYAGCKRKFDVDAYISYVKRLVPRAKKRKTEEGESTVKESNV

36.

>gi|350536405|ref|NP_001234245.1|_cryptochrome_2_Solanum_lycopersicum

MESNYKTIVWFRRDLRIEDNPALAAAARNGSVLPVFIWCPKEEGQFYPGRVSRWWLKQSLIHLKQSLKSLGAELVLMKAQSTLSALTECVDAVGATKVVYNHLYDPVSLVRDHNIKQKLGDLGISVQSYNGDLLNEPWEVYDDDGKVFTTFDAYWEKSLSIQNEPVSQLPPWRLTQAAGSVKMCSVEELGLENESEKSSNALLGKGWAPGWSNADKALTEFVESNLLAYSKDRLRVGGNSTSLLSPYLHFGEVSVRKVFNSVRLKQILWTKEGNSVGKDSATIYLRAIGLREYSRYICFNFPFTHERSLLNNLRFFPWNADQAHFKAWRQGRTGYPLVDAGMRELWATGWVHNKIRVIVSSFFVKFLLLPWQWGMKYFWDTLLDADLESDIIGWQYISGSLPDGHELERLDNPEVQGFNYDPEGEYVRHWLPELARMPAEWIHHPWDAPLNVLKAAGVELGMNYPNPIIDVDVARDRLMQAIIIMREKEAAVNTSHANGTVEVVFDNSENVGDSASIPKDDVVKGKEPCPSSSSYDQRVPSMQNVGTYRKRPKPEEETKKLNDNKLSYKNERIKMSNVDGDLCSTAESSSMKKQMTVSRNSFSVPRTITMSHDRKSFDDEASSHVKLQKEEEIDT

37.

>gi|350537989|ref|NP_001234577.1|_cryptochrome_1b_Solanum_lycopersicum

MSSGGCSIVWFRRDLRLEDNPALAAAVRAGSVIAVFIWAPEEEGYYCPGRVSRWWIKKSLAHLDSSLKKLGTSLITKRSNDSVSSLLQVVKSTGATRVFFNHLYDPISLVRDNCAKETLSAEGVSVCSFNADLLYEPWEVVDDESRPFSTFSDFWEKCLTMPYDPEAPLLPPKRIISGDASRCPSDNLVFESELEKGSNALLARAWSPGWSNADKALTTFINGPLIEYSKNRSKADSATTSFLSPCLHFGEVSVRKVFHRIRTKQTLWANEGNKAGEESVNLFLKSIGLREFSRYMSFYHPYSHERPLLGQLKYFPWLVDEGYFKAWRQGRTGYPLVDAGMRELWATGWLHDRIRVVVSSFSVKVLQLPWTWGMKYFWDTLLDADLESDALGWQFITGTLPDGCEFLGIDNPQFEGYKFDPNGEYVRRWLPELARLPTEWIHHPWDAPESVLQAAGIELGSNYPFPIVEIVAAKERLEEALSQMWQLEAAARSAIENGMEEGHGDSTDEFVPIAFPQAMQIEMEANNVPVRNNNPTITALRRYGDQIVPSMSSSFFRNEDEETSVDIRNSVVDSRAEVPIISM

38.

>gi|350539503|ref|NP_001234667.1|_cryptochrome_1_Solanum_lycopersicum

MSGGGCSIVWFRRDLRVEDNPALAAGVRAGAVIAVFIYAPEEEGHYYPGRVSRWWLKQSLAHLDSSLKSLGTSLITKRSTDSISSLLEVVKSTGATQLFFNHLYDPISLVRDHRTKEILTAQGISVRSFNADLLYEPWEVNDDEGRPFTTFSAFWEKCLSMPYDPEAPLLPPKRIISGDASRCPSDNLVFEDESEKGSNALLARAWSPGWSNADKALTTFVNGPLLEYSQNRRKADSATTSFLSPHLHFGEVSVRKVFHFVRIKQVLWANEGNKAGEESVNLFLKSIGLREYSRYMSFNHPYSHERPLLGHLRYFPWVVDEGYFKAWRQGRTGYPLVDAGMRELWATGWLHDRIRVVVSSFFVKVLQLPWRWGMKYFWDTLLDADLESDALGWQYISGTLPDGRELDRIDNPQFVGYKCDPHGEYVRRWLPELARLPTEWIHHPWNAPESVLEAAGIELGSNYPLPIVEIDSAKVRLEQALSQMWQNDAAARAAIENGMEEGHGDSADSPIAFPQAMHMEMDHEPVRNNPVIVTVRRYEDQMVPSMTSSLFRAEDEENSVDIRNSVVESRAEVPTDINVAEVHRRDTRDQAVMQTARTNATPHFNFAVGRRNSEDSTAESSSSTRERDGGVVPTWSPSSSNYSDQYVGDDNGIGTSSSYLQRHPQSHQLMNWQRLSQTG

39.

>gi|351734424|ref|NP_001235220.1|_cryptochrome_2_Glycine_max

MGSNRTIVWFRRDLRIEDNPALTAAAKEGSVLPVYVWCPKEEGQFYPGRVSRWWLKQSLAHLDQSLKSLGSRLVLIKTHSTAVALVECVKAIQATKVVFNHLYDPVSLVRDHNIKEKLVEQGISVQSYNGDLLYEPWEVNSESGRAFTTFNAFWKKCLHMQMDIVSVVPPWQLIPAEGKIEECSLEELGLENESEKPSNALLGRAWSPGWRNADKALREFVELHLLHYSKKRLKVGGESTSLLSPYLHFGELSARKVFQVTCMKQILWTNEGNSAGEESANLFLRAIGLREYSRYLCFNFPFTHERALLGHLKFFPWNPDPDIFKTWRQGRTGFPLVDAGMRELWATGWIHNRIRVIVSSFAVKMLLLPWKWGMKYFWDTLLDADLESDILGWQYISGGLPDGHELERLDNPEIQGAKFDPEGEYVRQWLPELARMPTEWIHHPWDAPLTVLRAAGVELGQNYPKPIIDIDLARERLTEAIFKMWESEAAAKAAGSEPRDEVVVDNSHTVENLDTQKVVVLGKAPCATISANDQKVPALQDSKNEPPTRKRPKHMIEEGQNQDHSQNHNKDTGLSSIDQDICSTADSSSCKKQCASTSSYSFSVPQQCSSSSNLKWPWQEKIDMEQSSSKDGAM

40.

>gi|1154067538|ref|NP_001238710.2|_CPD_photolyase_Glycine_max

MASTASPMTVQAGRVRTLKEGSRGESGLGPVVYWMFRDQRVTDNWALIHAVAEANKANVPVAVVFNLFHTFLGAKSRHLGFMLRGLRQLCHRMQHSLQIPFFLFQGEAEETVPKFLRECGASLLVTDFSPLREVRRCKEEICKRVSDSVAVHEVDAHNVVPLWVASDKLEYSARTIRAKITKRLSDYLVDFPDIEVEPPAGKWVATENHSIDWDDLIADVLRRGAEVPEVDWCEPGEIAASEVLMGSKNGFLTKRLKGYSLDRNNPCHPNALSGLSPYLHFGQISAQRCALEARKRRNSHPQAIDAFLEELIVRRELADNYCFYQPHYDSLKGAWAWAQNTLTEHATDKREHIYTKEQLEKAQTADPLWNASQLEMVHYGKMHGFMRMYWAKKILEWTRGPEEALEISLYLNDKYELDGRDPNGYVGCMWSICGVHDQGWKERPIFGKIRYMNYAGCKRKFDVDKYIAYVNKLVRELKKRKAENLLSQKEKVVRSCDPED

41.

>gi|525344850|ref|NP_001266969.1|_cryptochrome_2_(photolyase-like)_Fragaria_vesca

MGSSNKTIVWFRRDLRIEDNPALAAAARDGAVFPVYIWCPKDEGHFYPGRVSRWWLKQSLAHLDQSLKSLGAQLALIKTDSTVSALLDCIQAIGATKVVFNHLYDPVSLVRDHNIKGKLVELGISVQSYNADLLHEPWEVYDAKGQTFTTFKEYWDKCLNMERELVTFLPPWKLLQATGMVAKYSLEELGLENETEKSSNALLGRAWTPGWSQADKALTEFFDVHLLDYAKNRTKLGGNSTSLLSPYLHFGEVSVRKVFQLARMKQILFAKEGNSLGEESVTLFLRAIGLREFSRYICFNFPFTHEKLLLSNLRFFPWKADQGRFKAWRQGRTGYPLVDAGMRELWATGWIHNRIRVIVSSFAVKVLLLPWKWGMKYFWDTLMDADLESDILGWQYISGSLPDGHELERLDSPEVQGSKFDPDGEYVRHWLPELARLPTEWIHHPWDTPDNVLKVSGVELGVNYPRPIIEIDLARERLTEAIFKMWEIEAAAKAANLNGTNEVVVDNSDGIENFPIPKVILRNNTPCATYSSNDQKVPSCHNSEGNQLKRSRCTQERPLPDNGHNTNPNEATSRTIEDRSSTAESSMSKKQTTSTTSFSVPQSCSSSKDNPFMESESCEMKQSWQERIDMEQHSSKDGALEDECL

42.

>gi|525507079|ref|NP_001267582.1|_deoxyribodipyrimidine_photo-lyase-like_Cucumis_sativus

MASTLSNSVQPCRFRVLKDGTGSLGPVVYWMFRDQRVKDNWALIHAVDEANRANVPVAVAFNLFDRFLGAKSRQLGFMLRGLQQLQHDIQETLQIPFFLFQGEAEQTIPNFIRECGASLLVTDFSPLREVRKCKEEICKRVEESVKVHEVDAHNVVPTWVASEKLEYSAKTLRGKINKKLPDYLIDYPSMVIPTRKWPSADKFIDWDRLIDDNLRKGADVPELEWCKPGEKAAMEVLMGSKDGFLTKRLKGYAIDRNNPLKPKGLSGLSPYLHFGQISAQRCALEARSIRKLNPQAVDVFLEELIVRRELADNYCYYQPHYDSLLGAWEWARKTLMDHASDKREYIYTREQLEKAQTADPLWNAAQLEMAHHGKMHGFMRMYWAKKILEWTRGPEEALEICIYLNDKYEIDGRDPNGYVGCMWSICGVHDQGWKERPVFGKIRYMNYAGCKRKFDVDGYIAYVKRLVGEIKKRKPEETLEDRKPKGIRC

43.

>gi|823683792|ref|NP_001296304.1|_cryptochrome_DASH_chloroplastic/mitochondrial_Solanum_lycopersicum

MIKQPFLLTKFTPFSSKSKHTLFTFHCNFSIKMASLTARTTPTVQNVPGLTPEEMERVCEQTFQRYESGGLGKRKGKGVAIVWFRNDLRVLDNEALLRAWVSSEAILPVYCVDPRLFGTTHYFGMPKTGALRAQFIIECLNDLKRNLVKRGLDLLIQHGKPEDIVPSLAKAYKAHTVYAHKETCSEEVKVEKMVTRNLQKLVSPSSGGIGNDPGSGNTTKLELVWGSTMYHIDDLPFDCESLPDVYTQFRKSVEYKSKVRNCTKLPTSFGPPPEVGDWGHVPQVSELGLQQEKVSKGMNFVGGESAALGRVHDYFWKKDLLKVYKETRNGMLGADYSTKFSPWLASGSLSPRFIYEEVKRYEKERLSNDSTYWVLFELIWRDYFRFLSIKLANLLFQAGGPQKVNINWSQDQTMFDAWRRGQTGYPLIDANMKELAATGYMSNRGRQIVCSFLVRDMGIDWRMGAEWFETCLLDYDPCSNYGNWTYGAGVGNDPREDRYFSIPKQAQNYDPEGEFVAYWLPELRALPREKRHSPGMMYLNPIVALKHGYTKKTGDSKTAFSSRRGRPEDNRRKRHGY

44.

>gi|1485594596|gb|RKF54075.1|_Deoxyribodipyrimidine_photo-lyase_Golovinomyces_cichoracearum

MTISKFINVNCKSFCKSILSVIVHSPRSSYIQTTCQVSRTTLPQEMLPKQASSTNSIAVKRRCDTPEQIVTNKRLRQTLIPTDDSNRNSDPNEGDCASSSIIIRKYYPPEMSNERVLAYKEKKIARPIEELNSAQKETSKQRAEIKVGDSVVHWFKSDLRLKDNHSLYAASLKAKEAGIPLIAFYIISPQDFEAHKSAPIRVDFILRSLKSLRDDLAKLHIPLYTETVKKRKEIPDRIAELLSKWGSKHLFANIEYEVDELRREAGMVRKFASQGVDFDLRHDTCVVQPGKLVSGSGNQYSVYTPWFRAWLAYLHANLDLLQAFPGPNANSSSINSNENLKALFDHPIPSIPANKKLTLDETKRFAALWPVGETEALIRLEKFCKDRISAYKTHRNFPAETGTSSLSPYFAVGSLSARTAVIFAKEKSGAKKLDGGNEGTQTWISEVAWRDFYKHVLVEWPYVCMFKPFKPEYSNIKWESNNAHFIAWKEGRTGYPIVDAAMRQLAHTGWMHNRLRMITASFLAKHLLLDWRLGEQFFMLNLIDGDFASNNGGWGWSASSGVDPQPYFRIFNPELQSEKFDKDGEFIRKWVPELKAIKGSKLIHDPYGRGAASEAKKGGYVKKIVIHKEARERCLNRYKQGLGKAND

45.

>gi|501246294|ref|WP_012289312.1|_deoxyribodipyrimidine_photo-lyase_Halobacterium_salinarum

MQLFWHRRDLRTTDNRGLAAAAPGVTAVDGGHDQGPVAAVFCFDDEVLAHAAPPRVAFMLDALAALRERYRDLGSDLIVRHGDPAAVLPAVANDLDATRVVWNHDYSGLATDRDAGVRDALDAAGVAHAQFHDAVHHRPGEIRTNAGDPYSVYTYFWRKWQDREKNPPAPEPEPADLAADTALADTSPLPSVQELGFAEPEAAVPDAGTAAARSLLDAFRESGDIYRYEDRRDYPHEEPTSRLSPHLKFGTIGIRTVYEAARAAKSDADTDDERENVAAFIGQLAWREFYAQVLYFNQNVVSENFKAYEHPIEWRDDPAALQAWKDGETGYPIVDAGMRQLRAEAYMHNRVRMIVAAFLTKDLLVDWRAGYDWFREKLADHDTANDNGGWQWAASTGTDAQPYFRVFNPMTQGERYDPDADYITEFVPELRDVPADAIHSWHELSLSERRRHAPEYPDPIVDHSQRREDAIAMFERARGDE

46.

>gi|501349949|ref|WP_012381584.1|_deoxyribodipyrimidine_photolyase_Streptomyces_griseus

MSVAVVLFTSDLRLHDNPVLRAALRDADEVVPLFVRDDAVHRAGFDAPNRLAFLADCLAALDAGLRHRGGRLIVRRGEAATEVRRVAEETGAARVHIAAGVSRYAARREQRIREALADTGRELHVHDAVVTALAPGRVVPTGGKDHFAVFTPYFRRWEAEGVRGTLTAPRTVRVPDGVASDPLPDRDSVENLSPGLARGGEEAGRKLVTSWLNGPMADYEDGHDDLAGDATSRLSPHLHFGTVSAAELVHRAREKGGLGGEAFVRQLAWRDFHHQVLADRPDASWSDYRPRHDRWRSDADEIDAWKSGLTGYPLVDAAMRQLAHEGWMHNRARMLAASFLTKTLYVDWRVGARHFLDLLVDGDVANNQLNWQWVAGTGTDTRPNRVLNPVIQGKRFDARGDYVRRWVPELAEVEGSAIHEPWKLQGLDRAALDYPDPVVDLAEARARFERARGLD

47.

>gi|501552936|ref|WP_012557448.1|_deoxyribodipyrimidine_photolyase_Rhizobium_leguminosarum

MAKDAVKPVILWFRRDLRLDDNQALNAAHLSGRPIIPVYINEPAAAGTGPLGAAQAWWLHHSLEALDRSLHERQGELVLASGDALEVLRAVIKKSGAEAVFWNRRYDPSGISVDTHIKQELEKQAIEARSFGGQLLHEPSRLMTGNGTPYRVYTPFWRALEGAGEAEPPLEAPAKLRLASQRPASETLKSWKLLPTKPDWAKGFADLWTPGEQGARERLSAFVEDELKGYKENRDYPAKPATSMLSPHLALGEISPARIWDATRGLSNRVPAADIVHFRKEIAWREFSYHLLFHFPRLASENWNDRFDGFKWRNDDGDFEAWRRGMTGYPIVDAGMRQLWRHGWMHNRVRMIVASFLIKDLMIDWRDGEAWFRDTLVDADPANNAASWQWVAGSGADASPFFRIFNPMLQGETFDPDGDYVRAHVPELQRLGAKYIHRPFEAPKSALDEAGIILGQTYPKPIVDHASARDRALAAYKATKDAA

48.

>gi|518698395|ref|WP_019859900.1|_deoxyribodipyrimidine_photo-lyase_Mesorhizobium_loti

MSRQAQAPTIVLFRRDLRIGDNAALAAAADRGAPVVALYILDETTKGLRAMGAASRWWLHHSLAALGDLLRKAGANLFLAHGRTEDAVAKAIDASGANCVFWNRRYDPSEAGVDARLKAALREKGLTALSFDGALLHEPSLLKTGSGGFYKVYTPFWKAMAEEVDVRDPIDTPGQIDGWRGELGGLRLDELDLLPSKPDWAYGLRETWTPGEKGAQARLGQFIEHDLANYERQRDYPGQPSTSRLSPHLTFGEITPFQIFAALRRSKSSGTSKFRAEIGWREFSYHLLFHNPDLSGRNFRPEFDAMSWRDDMRALRTWQRGLTGYPIVDAGMRELWRTGWMHNRVRMIVASFLIKDLMIDWRHGEKWFWDTLVDADAANNPASWQWVAGSGADAAPYFRIFNPVLQGEKFDPHGDYVRQHVPEISALPDRYIHRPWEAPAAVLKDKGIVLGKTYPNPIVDHGAARERALIVYQSLKD

49.

>gi|1524057604|ref|WP_124105698.1|_deoxyribodipyrimidine_photo-lyase_Thermus_thermophilus

MGPLLVWHRGDLRLHDHPALLEALARGPVVGLVVLDPNNLKTTPRRRAWFLENVRALREAYRARGGALWVLEGFPWEKVPEAAKRLRAKAVYALRSYTPYGRHRDARVQEALPVPLHLLPAPHLLPPDLPKPYRVYTPFSRLYRGAAPPLPPPEALPKGPEEGEIPREDPGLPLPEPGEEAALRRLRAFLEAKLPRYAEERDRLDGEGGSRLSPYFALGVLSPRLAAWEAERRGGEGARKWVAELLWRDFSYHLLYHFPWMAERPLDPRFQALPWQEDEALFQAWYEGKTGVPLVDAAMRELHATGFLSNRARMNAAQFAVKHLLLPWKRAEEAFRHLLLDGDRAVNLQGWQWAGGLGVDAAPYFRVFNPVLQGERHDPEGRWLKRWAPEYPSYAPKDPVVDLEEARRRYLRLARDLARG

50.

>gi|50752305|ref|XP_422729.1|_deoxyribodipyrimidine_photo-lyase_Gallus_gallus

MPRGNGKGRKERDAGREEEEAVGTLEAAVREARRRTAPSVRDFRYNKQRARLVSRGSELKEGAECILYWMCRDQRVQDNWAFLYAQRLALKQELPLRVCFCLVPAFLDATIRHYGFMLRGLREVAKECAELDIPFHVLLGCPKDVLPSFVVEHGVGGLVTDFCPLRVPRQWVEEVKERLPEDVPFAQVDAHNIVPCWVASPKQEYSARTIRAKIHSQLPEFLTEFPPVIRHPHPPPNPPEPIAWDACYSSLQVDRTVTEVAWATPGTAAGLAMLQSFITERLKSFGSQRNDPNKAALSNLSPWFHFGQVSTQRAILEVQKHRRVYKESVDAFVEEAVVRRELAENFCYYNENYDSVRGAYDWAQSTLKLHAKDKRPFLYKLPQLEQATTHDPLWNAAQLQMVREGKMHGFLRMYWAKKILEWTRSPEEALQFAIYLNDRYELDGMDPNGYVGCLWSICGIHDQGWKERDVFGKIRYMNYAGCKRKFDVDQFERRYAHCK

51.

>gi|73983386|ref|XP_540761.2|_cryptochrome-2_isoform_X1_Canis_lupus_familiaris

MAAAVVAAAAAAPVPTAGVDGASSVHWFRKGLRLHDNPALLAAVRGARCVRCVYILDPWFAASSSVGINRWRFLLQSLEDLDTSLRKLNSRLFVVRGQPADVFPRLFKEWGVTRLTFEYDSEPFGKERDAAIMKMAKEAGVEVVTENSHTLYDLDKIIELNGQKPPLTYKRFQAIISRMELPKKPVGSVTSQQMESCRADIQDNHDDTYGVPSLEELGFPTEGLGPAVWQGGETEALARLDKHLERKAWVANYERPRMNANSLLASPTGLSPYLRFGCLSCRLFYYRLWDLYKKVKRNSTPPLSLFGQLLWREFFYTAATNNPRFDRMEGNPICIQIPWDRNPEALAKWAEGKTGFPWIDAIMTQLRQEGWIHHLARHAVACFLTRGDLWVSWESGVRVFDELLLDADFSVNAGSWMWLSCSAFFQQFFHCYCPVGFGRRTDPSGDYIRRYLPILKGFPSRYIYEPWNAPESIQKAAKCIIGVDYPRPIVNHAETSRLNIERMKQIYQQLSRYRGLCLLASVPSCVEDLSNPVAEPSSSQTGSMSSAGPRPLPSGPASPKRKLEAAEEPPGEELSKRARVAELPTPELPCRDV

52.

>gi|758984047|ref|XP_964834.2|_deoxyribodipyrimidine_photolyase_Neurospora_crassa_OR74A

MAPSKRKASAPPQTSHVNGNPSADKKRKTTTDAPPTNPNTSSDPLRAPHPFYKDSETHGIVLRKFYPHEMSNARAQAYNDNELPRPIETLSAALAETAALRKSLPVRQAVVHWFKMDLRLHDNRSLWLASQKAKEAGVPLICLYVLSPEDLEAHLRAPIRVDFMLRTLEVLKTDLEDLGIPLWVETVEKRKEVPTKIKELMKSWGASHLFCAMEYEVDELRREAKLVKLLAEGEKGEKMAADVVHDTCVVMPGALQSGSGGQYAVYSPWFRAWIKHIEENPECLEIYEKPGPNPPGTKEKHENLFACSIPEAPEGKRLRDDEKARYHSLWPAGEHEALKRLEKFCDEAIGKYAERRNIPAMQGTSNLSVHFASGTLSARTAIRTARDRNNTKKLNGGNEGIQRWISEVAWRDFYKHVLVHWPYVCMNKPFKPTYSNIEWSYNVDHFHAWTQGRTGFPIIDAAMRQVLSTGYMHNRLRMIVASFLAKDLLVDWRMGERYFMEHLIDGDFASNNGGWGFAASVGVDPQPYFRVFNPLLQSEKFDPDGDYIRKWVEELRDLPELKGGKGGEIHDPYGRGSEKVKKKLEEKGYPRPIVEHSGARDRALDAYKRGLARDL

53.

>gi|758983086|ref|XP_965722.3|_cryptochrome_DASH_Neurospora_crassa_OR74A

MAPSKVVIYAMRRELRLSDNPIFHHLSNPESKHGFSHLLPVYVFPAQQIDLSGFVPKGSENPHPAPKSAVGGYARCGPYRAKFLAESVWDLKTSLQSIGSDLLVRAGPYKDVIQSLVEGLKAKECQVGAVWMTSHEGSEEKSEEKTVASFCAKSGIDFKLWDDEKYLIHDRDTGITHLNDLPDVFTTYRKQIEPLREKARKTLPVPEKGALPAYPDIDMIPSQQPPFNIPGTCEELVDAVVRPVKNFLKDLPDFPEKAESSHPFRGGETSAHKRIDHLVLSGGMKSYKDSRNGLLGPDFSTKLSAYLAQGCVTARQIHHALVAYEDGTGTKYKGADGFGEGDNQGTETVRMELLWRDYMRLCHQKYGDKLFRVEGFNGKHTDYEGEDKKYGWRTANTSIALPGQEPTPEKVSEILARFNAGTTGMGLIDASQRELIHTGYTSNRTRQNVASFLAKHLEIDWRYGAEWYEMLLVDYDVSSNWANWQYVAGVGNDPRGAARIFNPVKQAFDYDKDGTYVRTWVPEVAKFENLENVFQAWTASKEDLKTAGLEGNIMVTDPVKPIKFNLDHKPSKVKKRPFFRKRGTKTRDAQGSAESPGSSDSHSGSGGSPDGSGGGNIPSESNCAAAGSGQAQQTHQGSGRSQSSSNHGGRSHSHQHNQQNYHHSHRGNDYTRGGGGGRGGRGGRGGGGGGYSASQGYYGIGGGYRGGGRGRGGGGGFRGRYAPTGGLGGHHHSEQQVASQFQTDA

54.

>gi|317036499|ref|XP_001397458.2|_DNA_photolyase_Aspergillus_niger_CBS_513.88

MPPQSAPTVIFWHRTDLRLHDNPALQAALSLNPSTFIPIFTWDPHYAYQVRVGPNRWRFLLECQNDLSQSYRKLNPKQKLWVVREAPQTVFPKLFKAWGATHLVFESDTDGYARERDETIRKLANEAGVEVIVKSGRTLFDSDEVVKQNKGEPTMSIHQVEKAIEQINNGVPDRPVDAPERIPDPLGEEKMRDISGLEHEVPDHEDDINAAHRTKHNDNQYNNIAGPKGDFSIPTLDELSIDPSQATSPHHGGESIALEMLTTYLQQNEDYIATFEKPKTSPAAFHPQATTLLSPHLHFGSLSVRKFWHDVQDTLQQRESAHKPTSDLPTNLPGQLLFREMFFAAQAALGPVYAQTRGNKIVRFVPWHLQSNHDKETGLVDRTYTVDDEQAEVWFRRWKEGRTGFPWIDALMRQLKNEGWIHHLGRHSVACFLTRGGCYVHWERGAEVFEEWLIDHETASNVGNWMWLSCTAFFTQYNRCYSPVAFGKKWDPEGRFIRHYIPELEHYDKKYIYEPWKAPLEDQKRWKCRVTGDGMVEKDEETGLRAYPEPMFDFDERRQTCIAQMKEAYEVHLMGNDEKVMDGSWKEIFEYEVKDGRVVDETNVKVDGDGGHRKGGEKRGRQAGDQDGEDEDGGHGLKKKK

55.

>gi|145258924|ref|XP_001402217.1|_deoxyribodipyrimidine_photlyase_Aspergillus_niger_CBS_513.88

MPPQKRKASSSSATNGASSHTNKREKPDLTRPHPHAKDTEDFGIVLRDFYPPEMCNERCEAYNNGTLERPIESLHRAYEDTFDERQKIRPNAAVVHWFKTDLRLHDNRGLQMAYQVAREHKLPLVGLYILSPEDLTAHLSSAPRVDLMLRTLELLRRDLSELDIPLYMETQEKRNDIPQRIIDLCQEWGATHLFANLEYEVDELRREAKLVRLCARNGIRFEAAHDTCVVTPGKLVSQQGKQYAVYSPWFRAWCAFLNENPEYLEVADEPGSNPGDARKHFKTLFGCEVPVAPENKRLSEEEKTRFRELYPEGEHEALRRLEAFLEEKANDYDDLRNTLAGRNTSVLSPYFASGSLSARTAVFQARKKNKGHLKRNQTGYTSWISEVAWRDFYKHVLVHWPFICMNKCFKPEFTNLAWSYNKDHFNAWCEGKTGYPIVDAAMRQIKHAAWMHNRTRMVVSSFLSKDLLIDWRRGERYFMENLIDGDFASNHGGWGFGSSTGVDPQPYFRIFNPLRQSERFDPEGEYIRLWVPELRAIEGAAVHDPYGRGAGDIAEKNGYPRPIVDHSESRDRALENYKKVAQGR

56.

>gi|149742994|ref|XP_001499263.1|_cryptochrome-1_isoform_X1_Equus_caballus

MGVNAVHWFRKGLRLHDNPALKECIQGADTIRCVYILDPWFAGSSNVGINRWRFLLQCLEDLDANLRKLNSRLFVIRGQPADVFPRLFKEWNITKLSIEYDSEPFGKERDAAIKKLATEAGVEVIVRISHTLYDLDKIIELNGGQPPLTYKRFQTLISKMEPLEIPVETITSEVIEKCTTPLSDDHDEKYGVPSLEELGFDTDGLPSAVWPGGETEALTRLERHLERKAWVANFERPRMNANSLLASPAGLSPYLRFGCLSCRLFYFRLTDLYRKVKKNSSPPLSLYGQLLWREFFYTAATNNPRFDKMEGNPICVQIPWDKNPEALAKWAEGRTGFPWIDAIMTQLRQEGWIHHLARHAVACFLTRGDLWISWEEGMKVFEELLLDADWSINAGSWMWLSCSSFFQQFFHCYCPVGFGRRTDPNGDYIRRYLPVLRGFPAKYIYDPWNAPEGIQKVAKCLIGVNYPKPMVNHAEASRLNIERMKQIYQQLSRYRGLGLLASVPSNPNGNGGLMGYSPGENIPGCSSSGSCSQGSGILHYAHGDSQQTHLLKQGRSSLGPGLSSGKRPGPEEDTQGIGPKVQRQSTT

57.

>gi|1377708916|ref|XP_001548442.2|_Bccry1_Botrytis_cinerea_B05.10

MFLVFPARNIFPKQLPSRLIISQRNILPLPQFLAKQQILHQSCISTSRILKMSTRKATAAAAASTKRKASSTPEPISHLNGSKKKAKVSDDDHDLSELRQPHHSAKEAEENGIVLRKYYPHEMCNPRAIAYNNDELERPIEALHAALADTQKERKGIDKGGECVVHWFKCDLRTKDNTALSMASQKAKELGIPLVTMYIVSPQDFEAHLTAPVRVDFILRTLDILKKDLAKLDIPLYVETIDKRKRVESRILELLGEWGCSHFYANMEYEVDELRREARMVRACVEKGICMDVIHDTCVVRPGELKSGQGKQYAVYTPWFKSWVAYVHENSDLLELFDAPEKNPSSARKTFAKLFETEIPDAPKNKSLTSEEKERFRSMWPAGEHAAHERLSKFADEKINKYQDHRNFPSLNSTSSLSVHFASGTLSSRTAIRTARDHNNTKKLNAGYQGIQTWISEVAWRDFYKHVLVHWPYVCMNKPFKPEYTNIEWEYNRAHFQAWTEGRTGYPIVDAAMRQLNHCGYMHNRSRMIVGSFLAKHLLLDWRMGERYFMEHLIDGDFASNNGGWGFCASTGVDPQPYFRIFNPLLQSEKFDAEGEYIRKWVEELKGVKGKAIHEPYGDKEAAKIAKKSGYPERIVEHKVSRERCLKRYKEGLGRANS

58.

>gi|219118604|ref|XP_002180071.1|_class_II_CPD_photolyase_Phaeodactylum_tricornutum_CCAP_1055/1

MLANRTRVLTSEGTEPKEGQSVVYWMQRDVRSVDNWALLWARDLAMQHDVPLHVVYALPPPASSDGSDNDRDLPPALIQLPMTKRHGAFLLGGLECVYKELKEMKIPLYVCLPDSHEKVGETVCEAILHKYKAKIVVSDFSPIREYRQWMELQAVPILEEAKVPFYQVDAHNIVPVWTATDKRQVGARTLRPRIHKVYNDYLQDYPDLKGNSHSVDQPKFDRVEYESFLQMDESVESVDWAQPGTEAGMKQFEFFSKNGLKIFHEQRNDPVQKHVCSDMSPWINHGHISFQRLALNVKALNKHANGAAAFIEEGVIRRELSDNMLYYSPNDYDSLETAAGWARESLQLHASDEREFVYSLSELEEGRTHDDLWNAAQLQMVRDGKMHGFMRMYWAKKILEWSESPVGALRTAQYLNDKYELDGRDPNGFVGVGWSIMGIHDQGWKEREVFGKIRYMNYNGCKRKFKVEEYVAQYKGAAENAANAVEETNGSSNKRKSLPSSSNSKQKTARK

59.

>gi|242096612|ref|XP_002438796.1|_cryptochrome_DASH_chloroplastic/mitochondrial_Sorghum_bicolor

MLHFISSSPLRPRFLLLPSPPSNLRFLAMSAAPSSSSSRSVRGVAVPVPSLSAGEACAVADEAFQRYTSPSLRRGGAGVAVVWFRNDLRVLDNEALLRAWSASEAVLPVYCVDPRVFAGSTHYFGFPKTGALRAQFLIECLGDLKQILRKKGLDLLVRHGKPEEILPSIAKAVSAHTIYAHKETCSEELLVERLVSKGLEQVQIAQGGASVPKKPLNPRLQLIWGATMYHIDDLPFPVSNLPDVYTQFRKAVESKSSVRNCTKLPPSLGPLPSSSIDEIGGWGAIPTLESLGLSVTKSEKGMHFIGGENAALGRVHEYFWKKDQLKDYKVTRNGMLGPDYSTKFSPWLASGSLSPRYICEEVKRYEKQRVANDSTYWVLFELIWRDYFRFLSAKYGNTIFHLGGPRKVVSKWSQDQALFESWRDGRTGYPLIDANMRELSATGFMSNRGRQIVCSFLVRDMGIDWRMGAEWFETCLLDYDPASNYGNWTYGAGVGNDPREDRYFSIPKQAKSYDPEGEYVAYWLPELRSLAKERRNFPGASYIRQIVQLKFDGGNQKKDQQFNRQRRPNNMYRRQVK

60.

>gi|242060916|ref|XP_002451747.1|_(6-4)DNA_photolyase_isoform_X1_Sorghum_bicolor

MEAATAATAAMVWFRKGLRVHDNPALDAARRYGAGAASARRLYPVFVLDPRYLRPDPAASSPGSARAGVARIRFLLESLSDLDARLRRLGSRLLLLRARDDVADAVCAALKDWNIGKLCFESDTEPYALARDKKVTDFALASGIEVFTPVSHTLFDPAEIIKKNGGRPPLTYQSFVSIAGEPPDPIMEEYSELPPLGDTGEYELLPVPTVEELGYVDISEEEIPPFRGGETEALRRMKESLQNKEWVAKFEKPKGDPSAFLKPSTTVLSPYLKFGCLSSRYFYHCIQDVYKSVRNHTKPPVSLIGQLLWRDFFYTVSYGTPNFDRMKGNKICKQIPWSENEELFVAWRDGQTGYPWIDAIMIQLRKWGWMHHLARHSVACFLTRGDMFIHWEKGRDVFERLLIDSDWAINNGNWMWLSCSSFFYQYHRIYSPITFGKKYDPNGNYIRHFIPVLKDMPREYIYEPWTAPLSIQKKANCIIGKDYPKPVVDHETASKECKKRMGEAYASSRLDANPTKGKTLNSSRRKMPHGDQDTSNSTISKLLKRNSRAE

61.

>gi|1275567720|ref|XP_003074697.2|_Cryptochrome/DNA_photolyase_class_1_Ostreococcus_tauri

MGRTRVVIWFRNDLRLLDNACVARAATLASESSDVEVVPVYVFDETYFKPSKRGLARFGAGRGKFTLECVGDLKTSLRALGSDLLVRCGKSRDVIAELTLTGANDRTIILTQTEVTSEETEMDVAVERATRERARGGAASATMERHWGSTLYHIDDVPFDVTSGLSDLPDVFTPFRNKVESKCKVRDVIPAPTANELGHVPASVEGFEWMPKPSDLPFASSEIAMDCDKRIKDCLDERSVLDFKGGESNALARVKYYLWESDRLATYFETRNGMLGGDYSTKLAPWLALGCVSPRHVVSEIRRYESERVENKSTYWVIFELIWRDFFKFFALKHGNKIFHLDGTAGRRASWKRDEKILKAWKTGTTGYPLIDANMRELAATGFMSNRGRQNVASWLALDAGIDWRHGADWFEHHLLDYDTASNWGNWCAAAGMTGGRINRFNIAKQTKDYDPAGEYIKTWVKELAEVPAAYIADPNQAPRELRDRIGLNYPNKLALPRRDFTEMGSPPGPRRGGGGGGRGRGRPGGSTPNRGTKARVASVYDTVYG

62.

>gi|1275569395|ref|XP_003078467.2|_DNA_photolyase_class_2_Ostreococcus_tauri

MAPACSKKRVRALTSNTEPMASATAPVMYWMSRDQRVDDNWALLRACDLARERGAPVVIAFNLLTKYLGAGARQFGFMLRGLRELEAKARAAKATFAMTYGDEPAAAIDALAKKIGAKTIVCDFSPLRDGVRWRKDLAVLSEKRGAHVEECDAHNVVPCWEASDKLEVGARTLRGRLAKRYPEFLKEFPAIPDDLVEYDGPAIDAVKWDDLLAEALKRGEAVPEVTWAIPGETAARAVLDDFVANRMKLYEKRNDPSKPRALSGLSPWLHFGQISAQRCALEAKKAVGKASPAAYDSFFEELVVRRELADNFCYYCPGQKYDEMEGQKYDWAKDTLRAHAGDKRPYIYTLEQLERAQTHDDLWNAAQRELRYGGKMHGFCRMYWAKKILEWTESPEQALKWSIYLNDTYSLDGRDPSGYVGCMWSIVGVHDQGWKEREVFGKIRYMAYDSTKKKFNIPDYIARVNALVKAAKSDFKTGEKSSAANPGLFSIDVSGVKRKADAMA

63.

>gi|348558770|ref|XP_003465189.1|_cryptochrome-2_Cavia_porcellus

MAAAVGTGTAAAPTPVTGAEGACSVHWFRKGLRLHDNPALLAAVRGARCVRCVYILDPWFAASSSVGINRWRFLLQSLEDLDTSLRKLNSRLFVVRGQPADVFPRLFKEWGVTRLTFEYDSEPFGKERDAAIMKMAKEAGVEVVTENSHTLYDLDRIIELNGQKPPLTYKRFQAIISRMELPKKPVGAVSSQQMESCRAEIQENHDDTYGVPSLEELGFPTEGLGPAVWQGGETEALARLDKHLERKAWVANYERPRMNANSLLASPTGLSPYLRFGCLSCRLFYYRLWDLYKKVKRNSTPPLSLFGQLLWREFFYTAATNNPRFDRMEGNPICIQIPWDRNPEALAKWAEGKTGFPWIDAIMTQLRQEGWIHHLARHAVACFLTRGDLWVSWESGVRVFDELLLDADFSVNAGSWMWLSCSAFFQQFFHCYCPVGFGRRTDPSGDYIRRYLPKLKGFPSRYIYEPWNAPESIQKAAKCIIGVDYPRPIVNHAETSRLNIERMKQIYQQLSRYRGLCLLASVPSCVEDLSHPVAEPSLSQAGSSTSTGPRPLPGGPASPKRKLEAAEEPPGEELSKRARVAELPTPEPPSKDA

64.

>gi|571472557|ref|XP_003531700.2|_(6-4)DNA_photolyase_isoform_X1_Glycine_max

MLRKGANPMILFKPSSAMRLSPCANTTSMSSGSGSLLWFRKGLRIHDNPALEVASRGASHLYPVFVIDPHFMEPDPNSSAPGSSRAGLNRIKFLLECLVDLDLNLKNLGSRLLILKGDPAEVVIRCLKELHVKKLCFEYDTEPYYQALDVKVKNFALAAGIEVFSPVSHTLFNPTDIIEKNGGKPPLSYQSFVKLAGEPPSSLSTVYSSLPPVGNLGSCDISEVPTIRDLGYGDAEQDEFSPFKGGESEALKRLDECMKDKKWVANFEKPKGNPSAFLKPATTVLSPYLKFGCLSPRYFYQSIQDVYKSMPKHTLPPVSLIGQLLWREFFYTAAFGTPNFDRMKGNRICKQIPWKDDDKLLEAWREARTGFPWIDAIMVQLRKWGWMHHLARHSVACFLTRGDLFVHWEKGRDVFERLLIDSDWAINNGNWMWLSCSSFFYQYNRIYSPTTFGKKYDPNGDYIRHFLPVLKDMPREYIYEPWTAPKSIQTKANCIIGKDYPMPVVSHDSASKECRRKMGEAYALNKELNGLVSEDDLKNLRRKLDESEGQEAGAKRYKQQLIG

65.

>gi|357139910|ref|XP_003571518.1|_(6-4)DNA_photolyase_isoform_X1_Brachypodium_distachyon

MDAATAVTATAGAAAAMVWFRKGLRVHDNPALDAARRGAARVYPVFVLDPRYLRPDPAAHSPGSARAGVARVRFLLESLSDLDAGLRRLGSRLLLLRARDDVPDALCAALRDWNIGKLCFEADTEPYALARDKRVTDFAAALGIEVFTPVSHTLFDPAEIIEKNGGRPPLTYQSFLAIAGEPPKPVMAEYSELPLIGDTGEYELLPVPKLEELGYGDISQENISPFRGGETEALKRMRESLQDKEWVSMFEKPKGDPSAFLKPATTVLSPYLKFGCLSSRYFYHCIQEVYRSTKKHTKPPVSLTGQLLWRDFFYTVSFGTLSFDHMKGNKICKQIPWRQNEELFVAWRDGRTGYPWIDAIMIQLRKWGWMHHLARHSVACFLTRGDLFIHWEKGRDVFERLLIDSDWAINNGNWLWLSCSSFFYQYHRIYSPISFGKKYDPNGDYIRHFIPVLKDMPKEYIYEPWTAPLSVQEKARCIVGRDYPKPVVDHEAASKECRKRMGEAYASNRLGGNTVNGKTSESSRRKSSDGGQDASDLSKSKQPKRRS

66.

>gi|357163871|ref|XP_003579874.1|_cryptochrome-1_isoform_X1_Brachypodium_distachyon

MSVSSSSMCGGDAGMKSVVWFRRDLRVEDNPALAAAARTAGEVVPAYVWSPEEDGPYFPGRVSRWWLSQSLKHLEASLQRLGAGKLVTRRSADAVVALLQLVRDTGATHLFFNHLYDPISLVRDHRLKEMLTAEGIIVQSFNADLLYDPWEVVDDEGHPFTMFMPFWNRCLSMPYDPPAPLLPPKRINSGDLLMCPSDDLIFEDDSERGSNALLARAWSPGWQNADKALTAFLNGPLVDYSVNRKKADSANTSLLSPYLHFGELSVRKVFHLVRMKQLVWSNEGNHAAEESCTLFLRSIGLREYSRYICFNHPCSHEKPLLAHLRFFPWVVNECNFKFWRQGRTGYPLVDAGMRELWATGWLHDRIRVVVSSFFVKVLQLPWRWGMKYFWDTLLDADLESDALGWQYISGSLPDSRELDHIDNPQLEGYKFDPHGEYVRRWLPELARLPTEWIHHPWDAPASVLQAAGVELGSNYPLPIIELDAAKSRLQEALSEMWQLEAASRAAMDTGMEEGLGDSSEVPPIEFPQDLQMEVHWEPARVTANVLTTARRRQDQMVPTMTSSLNRVETEISADLGNSVDSRAEVPSHMHVEPQTEREEMIRSTGNVVRTNDFRHHNNFQQPQHRMRDMFAASVSEASSSWTGREGGVVPVWSPPAASGHSETYVADEADVSSRSYLDRHPQSHRLMNWSQLSQSLTTGRDVENSVQPNFIG

67.

>gi|410965445|ref|XP_003989258.1|_cryptochrome-1_isoform_X1_Felis_catus

MGVNAVHWFRKGLRLHDNPALKECIQGADTIRCVYILDPWFAGSSNVGINRWRFLLQCLEDLDANLRKLNSRLFVIRGQPADVFPRLFKEWNITKLSIEYDSEPFGKERDAAIKKLATEAGVEVIVRISHTLYDLDKIIELNGGQPPLTYKRFQTLISKMEPLEIPVETITSELIEKCTTPLSDDHDEKYGVPSLEELGFDTDGLPSAVWPGGETEALTRLERHLERKAWVANFERPRMNANSLLASPTGLSPYLRFGCLSCRLFYFKLTDLYKKVKKNSSPPLSLYGQLLWREFFYTAATNNPRFDKMEGNPICVQIPWDKNPEALAKWAEGRTGFPWIDAIMTQLRQEGWIHHLARHAVACFLTRGDLWISWEEGMKVFEELLLDADWSINAGSWMWLSCSSFFQQFFHCYCPVGFGRRTDPNGDYIRRYLPVLRGFPAKYIYDPWNAPEGIQKVAKCLIGVNYPKPMVNHAEASRLNIERMKQIYQQLSRYRGLGLLASVPSNPNGNGGLMGYSPGENIPGCSSSGSCSQGSGILHYTHGDSQQTHLLKQGRSSMGTGLSGGKRPSQEEDTQSIGPKVQRQSVN

68.

>gi|460413920|ref|XP_004252323.1|_(6-4)DNA_photolyase_Solanum_lycopersicum

MASGANSLMWFRKGLRLHDNPALEYAAKGSKFLYPVFVIDPHYMDPDPTAFSLGSSKAGLNRIQFLLESLADLDLSLKKVGSRLLVLKGDPGELLIRCLKEWSIGKLCFEYDTEPYYQALDEKVKGYVSGTGVEIFSPVSHTLYNPADIIHKNGGSPPLSYQSFLKLAGQPSWAATPLSTTISSLPRIGNTGSFAVSEVPTVRELGYEDLPEDEKTPFKGGESEALKRLRESIANKEWVANFEKPKGNPSAFLKPATTVLSPYLKFGCLSSRYFYQCIQDILKCSKKHTSPPVSLLGQLLWRDFFYTAAFGTPNFDQMKGNRICKQIPWKNDDKLLAAWRDSKTGFPWIDAIMVQLRKWGWIHHLARHSVACFLTRGDLFVHWERGRHVFERLLIDSDWAINNGNWLWLSCSSFFYQYNRIYSPISFGKKYDPAGNYIRHFLPVLKDMPKEYIYEPWTAPISVQRKAKCIIGVDYPKPVVSHDSASKECKMRLGEAYALNKKLNGLVSEEDLNELRRKADNESTTLDSVSRKKKQKLID

69.

>gi|470457268|ref|XP_004341122.1|_type_II_CPD_DNA_photolyase_Acanthamoeba_castellanii_str._Neff

MDAPSASESRDEDFEVEQEEEQGRAKVAKERVRVLHEPKGKEGRKATGVVYWMSRDQRANDNWALLYAQQLAVKSGVPLAVAFCLLPSFKGASIRHFGFMVRGLTEVEKTLNSRGIPMLLLKGLPQDELPKALKTYGASHLVCDFSPLRIGRKWREEVAEATNAFVYEVDAHNLVPLWEVSPKQEYAARTIRPKIHKQVPKYLHEFPALRDHSKRNGGNKWSPAIPEVNWDSVWSYVREHVDDSVPELDWLKPGEKEGRRMLDLFLTKKLKDYNSKRNTPVEDGQSNLSAYLHYGQLSAQRIILEAMKHKAKAKESYEAYFEELIVRRELADNFCYYNQHYDQFEGFPDWARKSLEEHAADKRSSLYTYEELERGKTHDELWNAAQMEMVHLGKMHGFMRMYWAKKILEWTESPQQAMQFAVKLNDHYEIDGRDPNGYVGCAWAIGGVHDQGWKERPVFGKVRYMNYAGCKRKFDVDAYVRKMAKFMSEPTGTKKTEASAVSRKRKDKEGKKEKEKEEETNEAEEDEDEDEEEEGEEEEKQPAKKARRTAKRTVAQSAGSRAAARILKTKKSSSRGK

70.

>gi|502095106|ref|XP_004490375.1|_cryptochrome-1_isoform_X1_Cicer_arietinum

MTSGGCSIVWFRRDLRVEDNPALAAGVRAGAVVAVFIWAPEEEGQYYPGRVSRWWLKNSLSQLDSSLRSLGTPLITKRSTDSVSSLLEVVKNTGATQIFFNHLYDPLSLVRDHKAKEVLTTQGITVRSFNSDLLYEPWDVNDEHDQPFTTFDSFWERCLSMPYDPQAPLLPPKRIIPGDVSRCPSDTLVFEDESEKASNALLARAWSPGWSNANKALTTFINGPLIEYAKNRRKADSATTSFLSPHLHFGEVSVKKVFHLVRIKQVFWANEGNQAGEESVNLFLKSIGLREYSRYISFNHPYSHERPLLGHLKFFPWVVNEGYFKAWRQGRTGYPLVDAGMRELWATGWLHDRIRVVVSSFFVKVLQLPWRWGMKYFWDTLLDADLESDALGWQYISGTLPDGREFDRIDNPQFEGYKCDPNGEYVRRWLPELARLPTEWIHHPWNAPESVLQAAGIELGSNYPLPIVEIDAATVRLEEALIQMWQLEAASRAAAENGTEEGLGDSSESTPIAFPQDIQMEEIHEPVRNNPPHGTRRYEDQMVPSITSSRVRMEEEETSSVRNSGEDSRAEVPTNANGQQNTRETESQGVLQNVNRNTRQRNNTPTTFWLRNAPEDSTAESSSSTRRERDGGVVPEWSPPTSNFSDQFVDDENGIGSSSPYLQRHPQSHQLMSWTRLPQTG

71.

>gi|502132182|ref|XP_004501253.1|_cryptochrome_DASH_chloroplastic/mitochondrial_Cicer_arietinum

MAIFLFTTTLPFLSLPTTTLNSSKSTLTSILTNPTTLHHFPAIAMNLCTTTSAASCSSMQHVPEQDSNQTERVANLIFQRYTSNNTNRSGKGTAIVWFRNDLRVLDNEALYKAWLSSQTILPVYCVDPRLFATTYHFGFPKTGALRAQFLLESLADLRKNLMKRGLNLLIQHGKPEDVLPSLAKTFGAHTVYAQKETCSEEVNVERSVSRCLQQVGVPSEESVGAPTTSNSHPKLQFVWGTTMYHHGDLPFDVSCLPDVYTQFRKAIEAKCTVRSCIKLPASLGPPPAIEDWGCLPSLEQLGLYSQNVSKGMKFVGGETAALSRVYEYFWKKDLLKVYKETRNGMLGPDYSTKFSPWLASGSLSPRLIYEEVKRYENERQANSSTYWVLFELIWRDYFRFLSVKYGNLLFHIGGPRNVQHNWSQDKKLFEAWRDGCTGYPLIDANMKELSTTGFMSNRGRQIVCSFLVRDMGIDWRMGAEWFETCLLDYDPCSNYGNWTYGSGVGNDPREDRYFSIPKQAQTYDPEGEYVAYWLPQLRIIAKDKRNFPGNLYIRQIVPLKFGTSSRHNKDDKSLGARRANDKGNERRWNRR

72.

>gi|514708349|ref|XP_004951515.1|_(6-4)DNA_photolyase_Setaria_italica

MEAATAATAAAMVWFRKGLRVHDNPALDAARRGAGRLYPVFVLDPRYLRPDPAAASPGSARAGVARVRFLLESLGDLDARLRRLGSRLLLLRARDDVADAVCAALKDWNIGKLCFESDTEPYALVRDKKVTDFAMASGIEVFTPVSHTLFDPAEIINKNGGRPPLTYQSFIAIAGEPPEPLMEEYSELPPVGDTGEYELLPVPTVEELGYGDISQEEIPPFRGGETEALRRMKESLENKEWVAKFEKPKGDPSAFLKPATTVLSPYLKFGCLSSRYFYHCIQDVYRSVRNYTKPPVSLTGQLLWRDFFYTVSFGTPNFDQMKGNKICKQIPWSENEELFVAWRDSRTGYPWIDAIMIQLRKWGWMHHLARHSVACFLTRGDLFIHWEKGRDVFERLLIDSDWAINNGNWLWLSCSSFFYQYHRIYSPITFGKKYDPNGNYIRHFIPALKDMPREYIYEPWTAPLSIQKKAKCIIGKDYPKPVVDHETASKECRKRMGEAYASSRLDSNPSRGKPSNMSRRKKSHGDQGASNSSIAKLMKRSRAE

73.

>gi|528487228|ref|XP_005166949.1|_cryptochrome-1_isoform_X1_Danio_rerio

MAPNSIHWFRKGLRLHDNPALQEAVRGADTVRCVYFLDPWFAGSSNLGVNRWRFLLQCLDDLDSNLRKLNSRLFVVRGQPANVFPRLFKEWKISRLTFEYDSEPFGKERDAAIKKLAMEAGVEVIVKTSHTLYNLDKIIELNGGQPPLTYKRFQTLISRMDPPEMPVETLSNSIMGCCVTPVSEDHGDKYGVPSLEELGFDIEGLPSAVWPGGETEALTRIERHLERKAWVANFERPRMNANSLLASPTGLSPYLRFGCLSCRLFYFKLTDLYRKVKKTSTPPLSLYGQLLWREFFYTAATTNPRFDKMEGNPICVRIPWDKNPEALAKWAEAKTGFPWIDAIMTQLRQEGWIHHLARHAVACFLTRGDLWISWEEGMKVFEELLLDADWSVNAGSWMWLSCSSFFQQFFHCYCPVGFGRRTDPNGDFIRRYLPILRGFPAKYIYDPWNAPDSVQAAAKCIIGVHYPKPMVNHAEASRLNIERMKQIYQQLSRYRGLGLLASVPSTHNGNGNGMAYSPGEQQSGTNTPAPAVSSGSVASGNRSGSILLNFDSEEHQGPSGIQQQHQQQQQQQQQQQLGYHHMPDSGHNSRFYKSNVSHDMAAGHLLHKGGSVTGKRERESERDLDGEDESLSTSHKLQRQIAEVTSVYASSGNQSSMRS

74.

>gi|528514795|ref|XP_005168334.1|_cryptochrome_circadian_clock_4_isoform_X1_Danio_rerio

MLPEAIWSPSEVFSSWRKSVAMSHRTIHLFRKGLRLHDNPSLLGALASSSALYPVYVLDRVFLQGAMHMGALRWRFLLQSLEDLDTRLQAIGSRLFVLCGSTANILRELVAQWGITQISYDTEVEPYYTRMDKDIQTVAQENGLQTYTCVSHTLYDVKRIVKANGGSPPLTYKKFLHVLSVLGEPEKPARDVSIEDFQRCVTPVDVDRVYAVPSLADLGLQVEAEVLWPGGESHALQRLEKHFQSQGWVANFSKPRTIPNSLLPSTTGLSPYLSLGCLSVRTFYHRLNSIYAQSKNHSLPPVSLQGQVLWREFFYTVASATPNFTKMEGNSICLQIDWYHDPERLEKWRTAQTGFPWIDAIMTQLRQEGWIHHLARHAVACFLTRGDLWISWEEGMKVFEEFLLDADYSVNAGNWMWLSASAFFHKYTRIFCPVRFGRRTDPQGEYLRKYLPVLKNFPSQYIYEPWKAPEDVQLSAGCIIGKDYPRPIVSHIEASQRNLALMRQVRTEQQTTAELTRDVADDPMEAGLKRELREEEGLLEEAESQCTSKRFSGSSDHKSRPCSWTPETLQLSELSGEVM

75.

>gi|542152115|ref|XP_005484958.1|_deoxyribodipyrimidine_photo-lyase_Zonotrichia_albicollis

MRRARGKRKAEATEVPCVNRRRTEGDEAIQEARRRAAPSVREFKYNKKRVRLVSQGSDLKDDARCILYWMCRDQRVQDNWAFLYAQRLALKQELPLHVCFCLVPKFLEATIRHYRFMLRGLQEVAEECAELNISFHLLLGYAKDVLPVFVTEHGVGGLVTDFSPLRLPRQWVEEVRERLPEDVPFAQVDAHNIVPCWVASPKQEYSARTIRGKIHAQLPEFLTEFPPVVRHPHPPSCPAEPIAWEACYSSLEVDHTVKEVEWATPGTAAGMAVLKSFIAERLKSFSSHRNDPNKAALSNLSPWLHFGQVSTQRAILEVQKQRRSYKDSVDAFVEEAVVRRELAENFCYYNENYDSVQGAYDWAQTTLKVHAKDKRPYLYSLQELEQGTTHDPLWNAAQLQMVREGKMHGFLRMYWAKKILEWTHSPEEALQFAIYLNDRYELDGRDPNGYVGCLWSICGIHDQGWAERPIFGKIRYMNYAGCKRKFDVEQFERRYSPTHSQ

76.

>gi|542165234|ref|XP_005491227.1|_cryptochrome-1_isoform_X1_Zonotrichia_albicollis

MGVNAVHWFRKGLRLHDNPALRECIQGADTVRCVYILDPWFAGSSNVGINRWRFLLQCLEDLDANLRKLNSRLFVIRGQPADVFPRLFKEWNIAKLSIEYDSEPFGKERDAAIKKLASEAGVEVIVRISHTLYDLDKIIELNGGQPPLTYKRFQTLISRMEPLEMPVETITPEVMKKCTTPVSDDHDEKYGVPSLEELGFDTDGLPSAVWPGGETEALTRLERHLERKAWVANFERPRMNANSLLASPTGLSPYLRFGCLSCRLFYFKLTDLYKKVKKNSSPPLSLYGQLLWREFFYTAATNNPRFDKMEGNPICVQIPWDKNPEALAKWAEGRTGFPWIDAIMTQLRQEGWIHHLARHAVACFLTRGDLWISWEEGMKVFEELLLDADWSVNAGSWMWLSCSSFFQQFFHCYCPVGFGRRTDPNGDYIRRYLPVLRGFPAKYIYDPWNAPESIQKAAKCIIGVNYPKPMVNHAEASRLNIERMKQIYQQLSRYRGLGLLATVPSNPNGNGNGGLMGYSPGESISGCGSTGGAQLGTGDGHSVVQSCALGDSHTGTSGIQQQGYCQASSILHYAHGDNQQSHLLQAGRTALGTGISAGKRPNPEEETQSVGPKVQRQSTN

77.

>gi|542165810|ref|XP_005491511.1|_cryptochrome_DASH_Zonotrichia_albicollis

MSGTAGTAICVLRCDLRAHDNQVLHWAQHNADFVIPLYCFDPRHYLGTHCYSWPKTGPHRLRFLLESVKDLRETLKKKGSTLVVRKGKPEDVVCDLITQLGSVTAVVFHEEATQEELDVEKGLCQVCGQHGVKVQTFWGSTLYHRDDLPFRPIDRLPDVYTHFRKALESGARVRPTLQMPDQLKPLAPGLQEGSIPTMEDFGQKDPVTDPRTAFPCSGGETQALMRLQYYFWDTNLVASYKETRNGLVGMDYSTKFAPWLALGCISPRYIYEQIQKYERERTANQSTYWVLFELLWRDYFRFVALKYGRRIFSLRGLQSKEIPWKKDLQLFNCWKEGRTGVPFVDANMRELSATGFMSNRGRQNVASFLTKDLGLDWRMGAEWFEYLLVDYDVCSNYGNWLYSAGIGNDPRENRKFNMIKQGLDYDGNGDYVRLWVPELQGLKGADIHTPWALSSAALSQAGVTLGETYPQPVVTAPEWSRHIHQRPGGSPHPRASKRGPAQYKDRGIDFYFSRKKDAC

78.

>gi|542167846|ref|XP_005492496.1|_cryptochrome-1_isoform_X1_Zonotrichia_albicollis

MLHRTIHLFRKELRLHDNPVLLAALESSEALYPVYILDTAFLTSSMHIGALRWNFLLQSLEDLHKNLGKLGSCLLVIQGQYELVLRDHIQKWNITQVTLDAEMEPFYKEMEANIQRLGAELGFKVLSLVSHSLYNTQRILDLNGGSPPLTYKRFLHILSLLGDPEVPVRNLTAEDFQRCRAPDPGLAECYRVPLPMDLKISLESLSPWRGGETEGLQRLEQHLTDQGWVTSFTKPRTIPNSLLPSTTGLSPYFSMGCLSVRTFFYRLSNIYAQAKHHSLPPVSLQGQLLWREFFYTVASATPNFTQMAGNPICLQICWYKDAERLHKWKTAQTGFPWIDAIMTQLRQEGWIHHLARHAVACFLTRGDLWISWEEGMKVFEELLLDADYSINAGNWMWLSASAFFHQYTRIFCPVRFGKRTDPQGDYIRKYLPILKNFPSKYIYEPWTASEEEQKQAGCIIGQDYPFPIVNHKEASDHNLQLMKQVREEQHRTVQLTRDDADDPMEIKVKRDHSEENIAKGKVARTAE

79.

>gi|545515038|ref|XP_005625992.1|_cryptochrome-1_Canis_lupus_familiaris

MGVNAVHWFRKGLRLHDNPALKECIQGADTIRCVYILDPWFAGSSNVGINRWRFLLQCLEDLDANLRKLNSRLFVIRGQPADVFPRLFKEWNITKLSIEYDSEPFGKERDAAIKKLATEAGVEVIVRISHTLYDLDKIIELNGGQPPLTYKRFQTLISKMEPLEIPVETITSEVVEKCTTPLSDDHDEKYGVPSLEELGFDTDGLPSAVWPGGETEALTRLERHLERKAWVANFERPRMNANSLLASPTGLSPYLRFGCLSCRLFYFKLTDLYKKVKKNSSPPLSLYGQLLWREFFYTAATNNPRFDKMEGNPICVQIPWDKNPEALAKWAEGRTGFPWIDAIMTQLRQEGWIHHLARHAVACFLTRGDLWISWEEGMKVFEELLLDADWSINAGSWMWLSCSSFFQQFFHCYCPVGFGRRTDPNGDYIRRYLPVLRGFPAKYIYDPWNAPEGIQKVAKCLIGVNYPKPMVNHAEASRLNIERMKQIYQQLSRYRGLGLLASVPSNPNGNGGLMGYSPGENIPGCSSSGSCSQGSGILHYAHGDSQQTHLLKQGRSSMGTGLSSGKRPSEEEDTQTISPKVQRQSTN

80.

>gi|552844479|ref|XP_005851091.1|_CPD_photolyase_Chlorella_variabilis

MSRDQRVRDNWALLHAAAEASKRGVPVAVAFNLVTEYLHAGARQFGFMVRGLRLMQPKLQALNIPLFLLKGDPLETVPQLVKDTGASLLVTDFAPLRLGRHWREGVAAKIKVPFHEVDAHNVVPVWVASDKREYAARTIRPKIHSKLPEFLTEFPQLEPQPEWSSGVTPEAVDWDALLAEVLERGKEVPEVRWCAPGEDAAMEALSGPKGFLGSKARLARYEEKRNDPTVPDALSGLSPYLHFGHLSPQRAAVEAARNKAVHKASVEGFLEELIVRRELADNYCFYVPNYDSLDAAYDWARQTLNDHRGDKREHVYTREQFEKGQTHDKLWNAAQAEMVHFGKMHGFMRMYWAKKILEWSASPEEALEISIWLNDKYELDGRDANGYVGCMWSIAGIHDQGWAERPVFGKIRFMNFAGCKRKFDVEKYVARIGALVRDIKAGQK

81.

>gi|558143037|ref|XP_006092785.1|_cryptochrome-1_isoform_X1_Myotis_lucifugus

MGVNAVHWFRKGLRLHDNPALKECIRGADTIRCVYILDPWFAGSSNVGINRWRFLLQCLEDLDSNLRKLNSRLFVIRGQPADVFPRLFKEWNITKLSIEYDSEPFGKERDAAIKKLATEAGVEVIVQISHTLYDLDKIIELNGGQPPLTYKRFQTLISKMEPLEIPVETITLEVIEKCTTPLSDDHDEKYGVPSLEELGFDTDGLPSAVWPGGETEALTRLERHLERKAWVANFERPRMNANSLLASPTGLSPYLRFGCLSCRLFYFKLTDLYKKVKKNSSPPLSLYGQLLWREFFYTAATNNPRFDKMEGNPICVQIPWDKNPEALAKWAEGRTGFPWIDAIMTQLRQEGWIHHLARHAVACFVTRGDLWISWEEGMKVFEELLLDADWSINAGSWMWLSCSSFFQQFFHCYCPVGFGRRTDPNGDYIRRYLPVLRAFPAKYIYDPWNAPESIQKVAKCLIGVNYPKPMVNHAEASRLNIERMKQIYQQLSRYRGLGLLASVPSNPNGNGGLMGYSPGENIPGCSSSGSYAQGSGILHYALGDSQQTHLLKQGRSSVGTGLSSGKRPSQEEDTQSIGRKVQRQSTN

82.

>gi|566147859|ref|XP_006368713.1|_deoxyribodipyrimidine_photo-lyase_Populus_trichocarpa

MASLSSPPTQNTIVQPGRIRVIKEGSRGQVGGGPVVYWMFRDQRLQDNWALIHAVDQANRSNVPVAVAFNLFDQFLGAKARQLGFMLRGLCQLQSHIEETLQIPFFLFLGEAEETIPAFLKDCGASLLVTDFSPLRQFRTCQDEICKRVSDSVTIHEVDAHNVVPIWVASEKLEYSARTLRGKINKLLPEYLIDFPMLQLPKNKWVAATKQSIDWNDLIDNVLRKGAEVPEIKWCEPGEDAAMEVLMGSKDGFLTQRLKNYSTDRNNPLKPKGLSGLSPYLHFGQISAQRCALEARKVRNLSPQSADAFLEELIVRRELADNFCFYQPNYDSIHGAWEWARKTLADHASDKREHIYSKEQLEKAQTADPLWNASQLEMVCHGKMHGFMRMYWAKKILEWTRGPEEALAISIYLNDKYEIDGRDPGGYVGCMWSICGIHDQGWKERPIFGKIRYMNYAGCKRKFNVDGYITYVKRIVGDIKKRKAENELHKTMKELPS

83.

>gi|568453348|ref|XP_006460239.1|_CPD_photolyase_Agaricus_bisporus_var._bisporus_H97

MEAAAVVDANPPYFELKQLIEHGMPDPDKGKVVFYWMRFADLRITDNRALHKASEQAQKDGIPLAVLFVLSPEDYFAHDRSSRRIDFVLRNLKLLQEAFSKLHIPLYVITHKPRRTLAERVVESIKEYGCRHLYANLEHEVDELRRDIRMWQLGERHGIQVNLFHDKCIVEPGVVLTKQARGYTIFTPYWRNWVDTLNANLGKYTEKCPVPLPNNESIRRDKKLSFLFQTSVPGSINGFELSDNDAANMKEFWPAGEATAIQVLDRFLKTKSRSSQLGAVDPLSPGAQDGANHNRIHKYHQSRDQMDRDTTSRLSVYLSAGVISVRECVRQTMQLTGSKKVDGNRSAGVGRWIQELAWRDFYTGILVHYPRVSMGRPYLEKYSRVVWENHQAPQDTTGVHEHHDSENFKKWKEGMTGVAIVDAAMRCLNKMGWVHNRARMIVAMYLTKDLMIDWRLGERYFMQTLIDGDLASNNGGWQWSASTGVDPCPYFRIFNPHSQSLNADPTGEFIRYWVPELQKLHGPEIHDPSASTADKLGYPRKVIEHSAARDRALRRFKNPGEA

84.

>gi|612392706|ref|XP_007511873.1|_CPD_photolyase_Bathycoccus_prasinos

MLLRTATRCCSPLLNNNRFALFKSSKRTPARRSATTCAFFPSSSLFTNLTTTTTTASATARGFQDHFARRSNRASSNRTLLTLLQSPNGGRFTTRRKKHTAAISSTFVVYAGTTNTTIDINENNNNNKNEDKNMIVHPDRVMLINDQPILREGNGPIVYWMSRDQRVNDNWAMLYAIELANKEKKPLVVVFNVVTKFLGAGARQFGFMLRGLREVESALEERDIPFKLLHGGDEPNAEIEKFCNEVNASAVVTDFSPLRLGLKWRDDFAKETKRSVRVVDAHNIVPCWVASPKLEVGARTLRGKLAKLYGDFMVPFPDNFPNVENKDAALHAKIKSVKTDWDDVLGQALERGKDVPEVTWAVPGEKAAMAVLDNFLTKRMSLYGLRNDPAKPQALSGLSPYLHFGQISGQRCAMKALEAKKGSNGKAVDVFFEELVVRRELADNFCYYSPQYDTIEGQKYDWAKDTLRMHAGDKREYTYTYEEFEQAKTHDNLWNAAQRELVYGGKMHGFMRMYWAKKILEWSDTPENALKYAIALNDRWSLDGRDPSGYVGCMWSIVGVHDQGWKEREIFGKIRYMAYSGCEKKFKIPEYIKRVDALVEAVQKCEVSYKSNPGAWEIGRDDVLPKVAKSDNDDDDERKSKKQKK

85.

>gi|670394653|ref|XP_008677763.1|_cryptochrome_2_isoform_X1_Zea_mays

MTASQSSMSGGGEPGVRTVVWFRRDLRVEDNPALAAAARTAGEVVPAYVWSPEEDGPYYPGRVSRWWLSQSLKHLDASLRRLGACRLVTRRSSDAVVALIDLVRSTGATHLFFNRLYDPLSLVRDHRVKEQLSAEGITVQSFNADLLYEPWEVLDDDGFPFTMFAPFWNRCLCMPDPAAPLLPPKRINSGDLSRCPWDELIFEDESEKGSNALLARAWSPGWQNADKALTAFLDGPLVDYSANHKKADSASTSLLSPYLHFGELSVRKVFHQVRMKQLMWSNDGDRAGEESCTLFLRAIGLREYSRYLTFNHPCSLEKPLLSHLRFFPWVVDEVHFKVWRQGRTGYPLVDAGMRELWATGWVHDRIRVVVSSFFVKVLQLPWRWGMKYFWDTLLDADLESDALGWQYISGSLPDGRELDRIDNPQFEGYKFDPHGEYVRRWLPELARLPTEWIHHPWDAPESVLQAAGVELGSNYPRPIVELDAANSRLQGALSEMWELEAASCAAIENGMEEGLGDSTDEPPIDFPQELRMEVDRQPAQPAIIHTPVVAGWRREDQRVPSMTSSLIRAETELTADFGNTSEDSRPEVPSNIHLQARAEREETVDGATGNTVRVNGNQQQQNLQNNMHRVLGIAPSISEASSSWTGREGGLVPVWSPPAASVHSDPYTADEADISSRSYLDRHQQSNTMMNWSQLSQSLTTGWEVDN

86.

>gi|723724759|ref|XP_010325452.1|_deoxyribodipyrimidine_photolyase_isoform_X1_Solanum_lycopersicum

MASAIPVVQSGRIRVVKQGSGPLVGPVVYWMFRDQRIRDNWALIHAVDQANKANVPVAIAFNLFDQFLGAKARQLGFMLRGLEKLQGNLESTLRIPFFLFQGEAIDTIPNFLKECGASLLVTDFSPLRDVRSWKEKICERVDESVTVHEVDAHNIVPLWVASNKLEYSARTIRGKINKLLPEYLIELPAIEPLKIKWSSSNAPIDWPKLVSDVVRKGAEVPELEWCEPGEDAAFEVLMGSKKGFLTTRLKTYSTDRNNPLKPQALSGLSPYLHFGQISAQRCALEANKVRKNYTQAVDTFLEEMIVRRELSDNFCYYQPQYDSLLGAWEWARKTLMEHASDKREHIYTREQLEKAQTADVCFGQLWNASQLEMVHYGKMHGFMRMYWAKKILEWTNGPEEALAITIYLNDKYHIDGRDPSGYVGCMWSICGVHDQGWRERPVFGKIRYMNYAGCKKKFNVDGYISYVKRLVGESKKRKAEVILDKKAKELRN

87.

>gi|1567546042|ref|XP_012014825.2|_cryptochrome-1_isoform_X1_Ovis_aries

MGVNAVHWFRKGLRLHDNPALKECIQGADTIRCVYILDPWFAGSSNVGINRWRFLLQCLEDLDANLRKLNSRLFVIRGQPADVFPRLFKEWNITKLSIEYDSEPFGKERDAAIKKLATEAGVEVIIRISHTLYDLDKIIELNGGQPPLTYKRFQTLISKMEPLEIPVETITSEVMEKCTTPLSDDHDEKYGVPSLEELGFDTDGLPSAVWPGGETEALTRLERHLERKAWVANFERPRMNANSLLASPTGLSPYLRFGCLSCRLFYFKLTDLYKKVKKNSSPPLSLYGQLLWREFFYTAATNNPRFDKMEGNPICVQIPWDKNPEALAKWAEGRTGFPWIDAIMTQLRQEGWIHHLARHAVACFLTRGDLWISWEEGMKVFEELLLDADWSINAGSWMWLSCSSFFQQFFHCYCPVGFGRRTDPNGDYIRRYLPVLRGFPAKYIYDPWNAPEGIQKVAKCLIGVNYPKPMVNHAEASRLNIERMKQIYQQLSRYRGLGLLASVPSNPNGNGGLMGYSPGENIPGCSSSASCTQGSGILHYAHGDSQQTHLLKQGRSSTAAGLGSGKRPSQEEDTQSVGPKVQRQSTN

88.

>gi|802581644|ref|XP_012069829.1|_cryptochrome-1_Jatropha_curcas

MGSNKTIVWFRRDLRIEDNPALASAARDGCVFPVFIWCPQEEGQFYPGRVSRWWLKQSLAHLGHSLNSLGAELVVIKTHSTLAALLDCINAIGATRVVFNHLYDPVSLVRDHNIKEKLVEVGISVQSYNGDLLLEPWEVYDESGHAFVTFDAYWDKCLHMQMEPVSHLPPWRLVPVAGAVEKCSLEELGLENEAEKSSNSLLGRGWSPGWSKADKALAEFVEQHLIDYSKNMLRVGGNSTSLLSPYLHFGELSVRKVFQCVQMKRLLWLKEENSAGKESVTLFLRSIGLREYSRYLCFNYPFTHERSLLSNLKYFPWDINQAHFKAWRQGRTGYPLVDAGMRELWATGWVHNRIRVIVSSFAVKVLLLPWRWGMKYFWDTLLDADLESDILGWQYISGSLPDGHELERLDSPEVQGSKFDPEGEYVRQWLPELARVPTEWIHHPWDAPLIVLKAAGLELGQNYPKPIIELDLARERLTEAIFKMWEMEATARASNSGGTNEVVVDNTVGTENLAIPKVLVKEKVPCHTDSSNDQKVPTVQKPKNFPDHRKRSKYMEEEIIKRPNLDKLQNHSGIEGTSRAEDDLCSTAESSAAKKQATSRCSFSVPQCCSTTESKPLYECESSDLKQPWQVQIDVEQSSSEDVATGT

89.

>gi|802623866|ref|XP_012076312.1|_cryptochrome_DASH_chloroplastic/mitochondrial_Jatropha_curcas

MAGLYSSISSLSLKKLIQPSQRISTLTYPILTAHSKLCIRQIMNSSSSSSSTLTCQVPALDSDEMDRIADHTFDRYSSKIVKRVGKGTAIVWFRNDLRVLDNEALFMAWVSSEAVLPVYCVDPRLFQTTYHFGFPKTGALRAQFIIECLADLRKNLMKKGLNLLIRHGKPEEILPSLAEAFAAHTVYAQKETCSEETNVERLISKALQQVKLSPSPEKSKSHGSSKSPKLQLVWGSTMYHMDDLPFYTNDIPDVYTQFRKSVEAKCAIRSCTKMPTSLAPPPSVEDWGCVPSIDDLGFQPQKVNKGMRFLGGESAALSRVYEYFWKKDLLKIYKETRNGMLGPDYSTKFSPWLASGSLSPRFIYEEVKRYEKERAANDSTYWVLFELIWRDYFKFISVKYGNSLFHLGGPRKVERRWTQDQRLFESWRDGCTGYPLIDANMKELLSTGFMSNRGRQIVCSFLVRDMGIDWRMGAEWFETCLLDYDPCSNYGNWTYGAGVGNDPREDRYFSIPKQAQTYDPEGEYVAYWLPQLQKLEKDKRHFPGKSYTMQVVPLKFRNPKKQYSQDRAYATRQTNHAGRQTKGFKR

90.

>gi|884859557|ref|XP_012998342.1|_cryptochrome-1_isoform_X1_Cavia_porcellus

MGVNAVHWFRKGLRLHDNPALKECIRGADTIRCVYILDPWFAGSSNVGINRWRFLLQCLEDLDANLRKLNSRLFVIRGQPADVFPRLFKEWNITKLSIEYDSEPFGKERDAAIKKLASEAGVEVIVRISHTLYDLDKIIELNGGQPPLTYKRFQTLISKMEPLEIPVETITSEVVEKCVTPLSDDHDEKYGVPSLEELGFDTDGLPSAVWPGGETEALTRLERHLERKAWVANFERPRMNANSLLASPTGLSPYLRFGCLSCRLFYFKLTDLYKKVKKNSSPPLSLYGQLLWREFFYTAATNNPRFDKMEGNPICVQIPWDRNPEALAKWAEGRTGFPWIDAIMTQLRQEGWIHHLARHAVACFLTRGDLWVSWEEGMKVFEELLLDADWSINAGSWMWLSCSSFFQQFFHCYCPVGFGRRTDPNGDYIRRYLPVLRGFPAKYIYDPWNAPEGIQKVAKCLIGVNYPKPMVHHAEASRLNIERMKQIYQQLSRYRGLGLLASVPSNPNGNGGLLGYAPGESTPGSGGGSCVPGSSSAGVSHCAQGEAPQAPPGRDPAGPGLGGGKRPSQEEDAQSTGHKIQRQSPD

91.

>gi|1197621000|ref|XP_013222407.2|_cryptochrome-2_partial_Columba_livia

LLFLICRFLLQSLEDLDNSLRKLNSRLFVVRGQPTDVFPRLFKEWGVTRLTFEYDSEPFGKERDAAIIKLAKEAGVEVVIENSHTLYDLDRIIELNGHKPPLTYKRFQAIISRMELPKKPVSTIMSQQMEACKVDIQENHDDVYGVPSLEELGFPTDGLAPAVWQGGETEALARLDKHLERKAWVANYERPRMNANSLLASPTGLSPYLRFGCLSCRLFYYRLWELYKKVKRNSTPPLSLYGQLLWREFFYTAATNNPKFDRMEGNPICIQIPWDRNPEALAKWAEGKTGFPWIDAIMTQLRQEGWIHHLARHAVACFLTRGDLWISWESGVRVFDELLLDADFSVNAGSWMWLSCSAFFQQFFHCYCPVGFGRRTDPSGDYVKRYLPKLKGFPSRYIYEPWNAPESVQKAAKCIIGVDYPKPMVNHAETSRLNIERMKQIYQQLSRYRGLCLLASVPSCVEDLSGPVTDSASGQGCSTSTAMRLSQADQSSPKRKHEGAEELCTEELYKRARVTDLPTAEIPGKSV

92.

>gi|927384477|ref|XP_013938084.1|_DNA_photolyase_PHR1_Trichoderma_atroviride_IMI_206040

MLARSVYTVTRSQITSTSYLPSLSSYSKFLQPKFTFSTMPPKGSKRKTASPVGKANGVETKRVKHEANQDELHQPHPFAKDAEEHGIVLRRFYPHEMSTSRAYAYNSNEIERPMEGLVAALEETAGARKQAKVRHAVVHWFKMDLRHSDNRSLALASAKAKEAGVPLICVYIISPQDFEAHLTSPVRVDFMLRTLSVLKDDLAALDIPLHVETVDKRKDIPRRILELMEEWGSNHLFANMEYEVDELRRETRMIRQFAENNMSFEVVHDTCVVPPGELHSGTGKQYAVYTPWYRAWMAHIHENLDLLEIYDRPEKNPGATRRTFKTLFDCPIPDAPKNKQLNDEEKKRFHGLWPCGEHEAMSRLEKFCDEAVTSYHDRRNIPGDNGTSCLSVHLASGTISSRTCVRTARDRNKTKRLDGGHQGIHVWISEVAWRDFYKHVLVNWPYVCMNKPFKPEYANIEWSYNMDHFEAWCEGRTGFPIVDAAMRQLNHMGYMHNRCRMIVASFLSKDLLIDWRMGEKYFMEHLVDGDFASNNGGWGFSASVGVDPQPYFRVFNPLLQSEKFDPNGEYIRKWIPELKALSDKEIHDPYNRGAGTKAKKQGYPKQIVDHKGARERALSAYKDGLERGI

93.

>gi|929426245|ref|XP_014121765.1|_cryptochrome-2_Zonotrichia_albicollis

MELPKKPVSTVISQQMETCKVDIQENHDDVYGVPSLEELGFPTDGLAPAVWQGGETEALARLDKHLERKAWVANYERPRMNANSLLASPTGLSPYLRFGCLSCRLFYYRLWELYKKVKRNSTPPLSLYGQLLWREFFYTAATNNPKFDRMEGNPICIQIPWDRNPEALAKWAEGKTGFPWIDAIMTQLRQEGWIHHLARHAVACFLTRGDLWISWESGVRVFDELLLDADFSVNAGSWMWLSCSAFFQQFFHCYCPVGFGRRTDPSGDYVKRYLPKLKGFPSRYIYEPWNAPESVQKAAKCIIGVDYPKPMVNHAETSRLNIERMKQIYQQLSRYRGLCLLASVPSCVEDLSGPVTDSASGQGCSTSTAVRLSQADQASPKRKHEGAEEPCPEELYKRAKVTDLPASEISGKSL

94.

>gi|951033962|ref|XP_014515872.1|_(6-4)DNA_photolyase_isoform_X1_Vigna_radiata_var._radiata

MGSGSGSLVWFRKGIRIHDNPALEFASRTASHLYPVFVIDPHYMKPDPNAFSPGSSRAGLNRIKFLLESLVDLDLNLKNLGSRLLILKGDPAEVVIHCLKEWNVSKLCFEYDTEPYYQALDVKVKNFALAAGIEVFSPVSHTLFNPTDIIHKNGGKPPLSYQSFVKLAGEPPSPLTTVYSSLPPVGHLGSCDIFEVPTITDLGYGEAEQDEFSPFKGGESEALKRLDECMKDKKWVANFEKPKGNPSAFLKPATTVLSPYLKFGCLSSRYFYQRIQDIYRSMPKHSSPPVSLSGQLLWREFFYTAAFGTPNFDRMKGNRICKQIPWKDDDKLLEAWREARTGFPWIDAIMIQLRRWGWMHHLARHSVACFLTRGDLFVHWEKGRDVFERLLIDSDWAINNGNWMWLSCSSFFYQYNRIYSPTTFGKKYDPNGDYIRHFLPVLKDMPKEYIYEPWTAPKSIQTKANCIIGKDYPIPVVSHDSASKECRRKMGEAYALNKELNGLVGEDDLKNLRRKLDESEREEPEGKRYKQKLIG

95.

>gi|966985534|ref|XP_014969885.1|_cryptochrome-2_Macaca_mulatta

MAATVATAAAVAPAPAPGTDGASSVHWFRKGLRLHDNPALLAAVRGARCVRCVYILDPWFAASSSVGINRWRFLLQSLEDLDTSLRKLNSRLFVVRGQPADVFPRLFKEWGVTRLTFEYDSEPFGKERDAAIMKMAKEAGVEVVTENSHTLYDLERIIELNGQKPPLTYKRFQAIISRMELPKKPVGSVTSQQMESCRAEIQENHDETYGVPSLEELGFPTEGLGPAVWQGGETEALARLDKHLERKAWVANYERPRMNANSLLASPTGLSPYLRFGCLSCRLFYYRLWDLYKKVKRNSTPPLSLFGQLLWREFFYTAATNNPRFDRMEGNPICIQIPWDRNPEALAKWAEGKTGFPWIDAIMTQLRQEGWIHHLARHAVACFLTRGDLWVSWESGVRVFDELLLDADFSVNAGSWMWLSCSAFFQQFFHCYCPVGFGRRTDPSGDYIRRYLPKLKGFPSRYIYEPWNAPESIQKAAKCIIGVDYPRPIVNHAETSRLNIERMKQIYQQLSRYRGLCLLASVPSCVEDLSHPVAEPSSSQAGSVNSAGPRPLPSGPASPKRKLEAAEEPPGEELSKRAKVAELPTPELPSKDA

96.

>gi|966974323|ref|XP_015008131.1|_cryptochrome-1_isoform_X1_Macaca_mulatta

MGVNAVHWFRKGLRLHDNPALKECIQGADTIRCVYILDPWFAGSSNVGINRWRFLLQCLEDLDANLRKLNSRLFVIRGQPADVFPRLFKEWNITKLSIEYDSEPFGKERDAAIKKLATEAGVEVIVRISHTLYDLDKIIELNGGQPPLTYKRFQTLISKMEPLEIPVETITSEVIEKCTTPLSDDHDEKYGVPSLEELGFDTDGLSSAVWPGGETEALTRLERHLERKAWVANFERPRMNANSLLASPTGLSPYLRFGCLSCRLFYFKLTDLYKKVKKNSSPPLSLYGQLLWREFFYTAATNNPRFDKMEGNPICVQIPWDKNPEALAKWAEGRTGFPWIDAIMTQLRQEGWIHHLARHAVACFLTRGDLWISWEEGMKVFEELLLDADWSINAGSWMWLSCSSFFQQFFHCYCPVGFGRRTDPNGDYIRRYLPVLRGFPAKYIYDPWNAPEGIQKVAKCLIGINYPKPMVNHAEASRLNIERMKQIYQQLSRYRGLGLLASVPSNPNGNGGFMGYSTENIPGCSSSGSCSQGSGILHYTHGDSQQTHLLKQGRSSMGTGLSGGKRPSQEEDTQSIGPKVQRQSTN

97.

>gi|971433169|ref|XP_015154168.1|_cryptochrome_4_isoform_X1_Gallus_gallus

MRHRTIHLFRKGLRLHDNPALLAALQSSEVVYPVYILDRAFMTSSMHIGALRWHFLLQSLEDLRSSLRQLGSCLLVIQGEYESVVRDHVQKWNITQVTLDAEMEPFYKEMEANIRGLGEELGFQVLSLMGHSLYNTQRILELNGGTPPLTYKRFLRILSLLGDPEVPVRNPTAEDFQRCSPPELGLAECYGVPLPTDLKIPPESISPWRGGESEGLQRLEQHLADQGWVASFTKPKTVPNSLLPSTTGLSPYFSTGCLSVRSFFYRLSNIYAQAKHHSLPPVSLQGQLLWREFFYTVASATPNFTKMAGNPICLQIRWYEDAERLHKWKTAQTGFPWIDAIMTQLRQEGWIHHLARHAAACFLTRGDLWISWEEGMKVFEELLLDADYSINAGNWMWLSASAFFHHYTRIFCPVRFGRRTDPEGQYIRKYLPILKNFPSKYIYEPWTASEEEQKQAGCIIGRDYPFPMVDHKEASDHNLQLMKQAREEQHRIAQLTRDDADDPMEMKLKRDHSEESFTKTKAARMTEQT

98.

>gi|992231580|ref|XP_015412200.1|_deoxyribodipyrimidine_photolyase_Aspergillus_nomius_NRRL_13137

MRRISPVIGLSRVTLLYSSTLSSRSRSTLTPTIVRNMPQKRKPSQNVTRENGTNSTSHSNKRGKADLSSPHPNARQAEEFGIVLRQFYPPEMSNERCQAYNDGTLERPMEALNRVCEETVDARLSIRPNAAVVHWFKSDLRLHDNRALRKAYELAREHSIPLIALYILSPEDLTAHLSSPARVDLTLRTLEQLKRDLGELDIPLYMETQEKRRGIPQRIIDLCQEWGANHLFANIEYEVDELRREAKLVRLCVENGIAFDLLHDTCVVPPGLLSSQQGKQYAVYSPWFRAWQVFLMDNPDYLEASEEPGANPGNTRKVFKSLFESEIPGAPDNKGLSDEEREHFRELYPAGEHEALDRLERFLEEKATDYDDMRNSLCKQTTSVLSPYFASGSLSARTAVAQAKKANKNQLDRNDLGFVSWISEVAWRDFYKHVLVHWPFICMNKCFKSEFTDLEWEYNEDYFNAWCEGKTGYPIVDAAMRQINSVAWMHNRSRMIVASFLSKDLLIDWRRGERYFMEHLIDGDFASNHGGWGFGSSTGVDPQPYFRIFNPLRQSERFDPDGEYIRRWVPELREIQGSAIHDPYERGAGDLAEKNGYPGPIVDHATRRALALDRYKKAAGGNSK

99.

>gi|1002303097|ref|XP_015614933.1|_deoxyribodipyrimidine_photolyase_isoform_X1_Oryza_sativa_Japonica_Group

MPPTSVSPPRTAPGPANPSPAHPSRVRVIHPGGGKPGGPVVYWMLRDQRLADNWALLHAAGLAAASASPLAVAFALFPRPFLLSARRRQLGFLLRGLRRLAADAAARHLPFFLFTGGPAEIPALVRRLGASTLVADFSPLRPVREALDAVVGDLRREAPGVAVHQVDAHNVVPVWTASAKMEYSAKTFRGKVSKVMDEYLVEFPELPAVVPWDREQPEGVDWDALIARVCSEAENVPEIDWCEPGEEAAIEALLGSKDGFLTKRIKSYETDRNDPTKPRALSGLSPYLHFGHISAQRCALEAKKCRHLSPKSVDAFLEELVVRRELADNFCYYQPQYDSLSGAWEWARKTLMDHAADKREHIYTREQLENAKTHDPLWNASQLEMVHHGKMHGFMRMYWAKKILEWTSGPEEALSTAIYLNDKYEIDGRDPSGYVGCMWSICGLHDQGWKERPVFGKIRYMNYAGCKRKFDVDAYISYVKRLAGQSKKRNAEESPNPVVKLSKSQH

100.

>gi|1002239385|ref|XP_015623929.1|_(6-4)DNA_photolyase_Oryza_sativa_Japonica_Group

MDAAATAATATAAAAMVWFRKGLRVHDNPALDAARRGGAAARLYPVFVLDPRYLRPDQAAPSPGSARAGVARVRFLLESLSDLDARLRRLGSRLLLLRARDDGDVAGTVCAALKDWNIGKLCFESDTEPYALARDKKVMDFAAASGIDVFSPVSHTLFDPAEIIEKNGGRPPMTYQSFVAIAGEPPEPIMEEYSELPPVGDTGEYELLPVPRVEELGYGDISQEDLSLFRGGETEALKRMRESLHDKEWVAKFEKPKGDPSAFLKPATTVLSPYLKFGCLSSRYFYHCIQDIYRSTKKHTNPPVSLTGQLLWRDFFYTVAFGTPNFDQMKGNKICKQIPWTENEELFPAWRDGRTGYPWIDAIMIQLRKWGWMHHLARHSVACFLTRGDLFIHWEKGRDVFERLLIDSDWAINNGNWMWLSCSSFFYQYHRIYSPTSFGKKYDPNGNYIRHFIPVLKDMPKEYIYEPWTAPLSIQKKANCIIGKDYPKPVVDHAIASKECKKMMGEAYASNRLDDDKPDKGKSSNSSRRKLSAGSQVTPNSSKTKQLKRSS

101.

>gi|1002264531|ref|XP_015636604.1|_cryptochrome-1_isoform_X1_Oryza_sativa_Japonica_Group

MSVSSSSMGGGGGGDAGGRTVVWFRRDLRVEDNPALAAAARAGGEVVPAYVWAPEEDGPYYPGRVSRWWLSQSLKHLDASLRRLGAGKLVTRRSADAVVALLQLVRDTGATRLFFNHLYDPISLVRDHRLKEMMAAEGIIVQSFNADLLYEPWEVVDDEGQSFTMFAPFWNRCLSMPYDPAAPLLPPKRINSGDLSMCPSDDLIFEDDSERGSNALLARAWSPGWQNADKALTAFLNGPLIHYSVNRKKADSASTSLLSPYLHFGELSVRKVFHLVRMKQLVWSNEGNRAAEESCTLFLRSIGLREYSRYLSFNHPCSHEKPLLAHLRFFPWVINECYFKIWRQGRTGYPLVDAGMRELWATGWLHDRIRVVVSSFFVKVLQLPWRWGMKYFWDTLLDADLESDALGWQYISGSLPDGRELDRIDNPQLEGYKFDPHGEYVRRWLPELARLPTEWIHHPWDAPASVLQAAGVELGSNYPLPIVGLDAANARLQEALSEMWQLEAASRAAMDNGMEEGLGDSSEVPPIEFPRELQMEVDREPARVTANVLTTARRREDQMVPTMTSSLNRAETEISADFMNSVDSRAEVPTRVNFEPRTEREENFRTTAGNVARTNGIHEHNNFQQPQHRMRNVLAPSVSEASSGWTGREGGVVPVWSPPAASDHSETFASDEADISSRSYLDRHPQSHRLMNWSQLSQSLTTGREVENSMQPNWIG

102.

>gi|1002279585|ref|XP_015644117.1|_cryptochrome_DASH_chloroplastic/mitochondrial_isoform_X1_Oryza_sativa_Japonica_Group

MLHFLSSSSPLNPQFLLLPRQSARLRVLLSIPVSAMSSSSSSSSRGALAAAAVPSLSADEAGAAADEAFLRYTSPSMRRSGGGGVAIVWFRNDLRVLDNEAVVRAWAASDAVLPVYCVDPRISAGSTHYFGFPKTGALRAQFLIECLEDLKRNLTKQGLDLLIRHGKPEDILPSIAKAVTAHTVYAHKETCSEELLVEHLVRKGLEQVVIPQGGASNQKKPRNPKLQLIWGATLYHVDDLPFSVNNLPDVYTQFRKAVESKSSVRNCSKLPPSLGPPPGSGLDEIGGWGTVPTLESLGLSMTKSEKGMHFVGGESAALGRVHEYFWKKDQLKVYKETRNGMLGPDYSTKFSPWLASGSLSPRYICEEVKRYEKQRIANDSTYWVLFELIWRDYFRFISAKYGNSIFHLGGPRNVESKWSQDQALFESWRDGRTGYPLIDANMKELLATGFMSNRGRQIVCSFLVRDMGIDWRMGAEWFETCLLDYDPASNYGNWTYGAGVGNDPREDRYFSIPKQAKTYDPDGEYVAYWLPELRSIAKERRNFPGASYIKQVVPLKFDGGHQKRDQQFNRQRRPGHMYRRQK

103.

>gi|1009576523|ref|XP_015919899.1|_deoxyribodipyrimidine_photolyase_isoform_X1_Parasteatoda_tepidariorum

MFGTSLILFNRGFTLFSIRLSKFTMSEKKHKLSEKDGPVKKAKLDETVQNDSDLVENIKKSRLSCAASIRDFKFNKKRVRVLTTAKDIPEEAKCIVYWMSRDQRVQDNWAFLYAQNLAFKINLPLCVCFCLVPKFLEATIRHYRFMLKGLQEVEEECKSLNIHFHLLLGESKNVLPKFVKDNQVGGVVTDFSPLRVPQKWVSELASKLPPDVPLCQVDAHNIVPCWVASDKQEYGARTIRKKIHDKLKEYLTDFPPVVKNKLTPECEFEPVDWNSVEKILEVNMDVDEVKWAIPGTTAGLKQLSSFCKDRLKHFHDCRNDPTKNNLSNLSPWFHFGQLSIQRTILVVSKLRSKYPASVDAFVEEAVIRRELSDNFCFYNKKYDQVDGAYDWAKKTLKDHSKDKREYIYTKEQFENAKTHDLLWNAAQRQLKKEGKMHGFLRMYWAKKILEWTNSPEEALEFAIYFNDKYNLDGRDPNGYVGCMWSICGIHDQGWAERAVFGKIRFMNFKGCQRKFDVNAFIQRYREK

104.

>gi|1034088063|ref|XP_016776215.1|_cryptochrome-2_Pan_troglodytes

MGGVHVAYRGGAGVAGAVWTVMAATVATAAAVAPAPAPGTDGASSVHWFRKGLRLHDNPALLAAVRGARCVRCVYILDPWFAASSSVGINRWRFLLQSLEDLDTSLRKLNSRLFVVRGQPADVFPRLFKEWGVTRLTFEYDSEPFGKERDAAIMKMAKEAGVEVVTENSHTLYDLDRIIELNGQKPPLTYKRFQAIISRMELPKKPVGSVTSQQMESCRAEIQENHDETYGVPSLEELGFPTEGLGPAVWQGGETEALARLDKHLERKAWVANYERPRMNANSLLASPTGLSPYLRFGCLSCRLFYYRLWDLYKKVKRNSTPPLSLFGQLLWREFFYTAATNNPRFDRMEGNPICIQIPWDRNPEALAKWAEGKTGFPWIDAIMTQLRQEGWIHHLARHAVACFLTRGDLWVSWESGVRVFDELLLDADFSVNAGSWMWLSCSAFFQQFFHCYCPVGFGRRTDPSGDYIRRYLPKLKAFPSRYIYEPWNAPESIQKAAKCIIGVDYPRPIVNHAETSRLNIERMKQIYQQLSRYRGLCLLASVPSCVEDLSHPVAEPSSSQAGSMSSAGPRPLPSGPASPKRKLEAAEEPPGEELSKRARVAELPTPELPSKDA

105.

>gi|1034098191|ref|XP_016779596.1|_cryptochrome-1_isoform_X1_Pan_troglodytes

MGVNAVHWFRKGLRLHDNPALKECIQGADTIRCVYILDPWFAGSSNVGINRWRFLLQCLEDLDANLRKLNSRLFVIRGQPADVFPRLFKEWNITKLSIEYDSEPFGKERDAAIKKLATEAGVEVIVRISHTLYDLDKIIELNGGQPPLTYKRFQTLISKMEPLEIPVETITSEVIEKCTTPLSDDHDEKYGVPSLEELGFDTDGLSSAVWPGGETEALTRLERHLERKAWVANFERPRMNANSLLASPTGLSPYLRFGCLSCRLFYFKLTDLYKKVKKNSSPPLSLYGQLLWREFFYTAATNNPRFDKMEGNPICVQIPWDKNPEALAKWAEGRTGFPWIDAIMTQLRQEGWIHHLARHAVACFLTRGDLWISWEEGMKVFEELLLDADWSINAGSWMWLSCSSFFQQFFHCYCPVGFGRRTDPNGDYIRRYLPVLRGFPAKYIYDPWNAPEGIQKVAKCLIGVNYPKPMVNHAEASRLNIERMKQIYQQLSRYRGLGLLASVPSNPNGNGGFMGYSAENIPGCSSSGSCSQGSGILHYAHGDSQQTHLLKQGKNEALEHTVLFPFPILNIHFLNVQEEAPWALVSVVGNVLVRKRTHRVLVLKSRDRALIRKHSGGILLQLKLVGSSILFN

106.

>gi|1070334966|ref|XP_018235323.1|_cryptochrome_Fusarium_oxysporum_f._sp._lycopersici_4287

MAKPRVIYWFRTDLRLHDSPALKAALDLDPAVLWPIFTWDPHYVYRARGGLNRWQFLLDCQNDLSRSISQVNPKSKLFVLREAPQTLFPKLFKAWKVTHLVFEKDTDSYGRERDGVVVQAAKDAGVEVLVRSGRTLWDSDQIVEKHGGKPTMSITQLQTAGSKLGEIRKPIPAPKHLPDPGDMPVNFEQDEPSTKPDFNAGFRTEGDKAYTRIAGPNDDFAIETMEELGFPPATTPHRGGETRALKELNKLIADKKYTATFQKPKTNPAQFEPQATTLLSPFLHFGALSVRLFYWRVREIVDSYGKGASTPPESLIGQLLFRDMYFAAQAALGYVFSQTANNPYCRFIPWHLPSKRDSETGLITGEYHIDSEEAEIWFRRWRVGMTGFPWIDALMRQLKDEGWIHHLGRHAVACFLTRGGCYIDWERGCEVFEEWLIDHEPACNAGNWQWLSCTAFFSQYFRCYSPIAFGQKWDKEGNFIRRYVPELKNMDSKYIYEPWKAPLPDQKKAGVRIKGDGVEQYRGGNISKTNV

107.

>gi|1070556872|ref|XP_018383275.1|_photolyase_Alternaria_alternata

MPPKRKASIPPNAASARNYTDVSGDAPNKRSRIAKPLSKAFADSAPPIDSIARNTENGAPGEAVKKEEEEEEEEEEEEEEEEEEEEEEEGEEGEEGEGEFDHSRPEERAGIVDRRYYPAEMSNERCALYNANEIPRPIEILAKTLESTKDRRMAIREANKAQFGDAVVHWFKRDLRIRDNTGLSQAAQLAKAKGVGVIGVWFMSPQDWEAHLVSPPKCDFELRSVESLKQELEEFDIPLYVETIAERKNVTKRLVELAESWNAKNVFCNLEYEPDELRREERLVRKMLEKGINFDPQHDDCVVPPGSLKTGGGKQYAVYSPWYRAWVAYLHAHPHLLNERPIPERNSPNFRQKFTQLFDSKVPDLPDCKSLTQEEKERFHRLWPAGEAAAIDRLERFLIEKIGKYKDTRNFPAKNSTGRVSVHHAAGTLAARTSVRMARDVNSAKKLDGGKDGVKGWIGEVAWRDFYRHVLVHWPYVCMNKPFKFEYTNIEWEYNDAHFQAWTQGRTGYPIVDAAMRCMNHTGYMHNRLRMIAASFLAKHLLLDWRLGEQYFLTHLVDGDFSSNNGGWGFSASTGVDPQPYFRIFNPWTQSERFDEEGEFIKLWVPELEEIEGPAIHNPYGAGGKAAQAAKSKGYPEPVVEHKFARERCLARYKAGIGRETA

108.

>gi|1092963636|ref|XP_018750188.1|_cryptochrome_Fusarium_verticillioides_7600

MSNPRVIYWFRTDLRLHDSPALKAALDLDPAVLWPIFTWDPHYVYRARGGLNRWQFLLDCQNDLSRSISQVNPKSKLFVLREAPQTLFPKLFKAWKVTHLVFEKDTDSYGRERDSVVVQAAKDAGVEVLVRSGRTLWDSDQIVEKHGGKPTMSITQLQTAGSKLGEIRKPIPAPKHLPDPGDMPVNFEQDEPNTKPDFNAGFRTEGDKSYTRIAGPNDDFAIETMEELGFPPATTPHRGGETLALKELNKLIADEKYTATFQKPKTNPAQFEPQATTLLSPYLHFGALSVRLFYWRVREIVDSYGNGASTPPESLLGQLLFRDMYFAAQAALGYVFSQTANNPYCRFIPWHLPSKRDSETGLITGEYHIDSEEADIWFRRWRVGMTGFPWIDALMRQLKDEGWIHHLGRHAVACFLTRGGCYIDWERGCEVFEEWLIDHEPACNAGNWQWLSCTAFFSQYFRCYSPIAFGQKWDKEGNFIRRYVPELKNMDSKYIYEPWKAPLPDQKKAGVRIKGDGLNSIEEGTYPKPMFDFAKRRDVCISAMKTAYQVGLHGNDGQALDGTWRKLFPTDRGEIQGDIESDGDEHAGYGDDEGGREDNEAKEKGEGIKSIENGDQSMSKRSSRRHSSENTTKRQKT

109.

>gi|1179529173|ref|XP_020685617.1|_(6-4)DNA_photolyase_isoform_X1_Dendrobium_catenatum

MESSSSSNCMIWFRKGLRIHDNPALELAQKGSKHLFPVFVLDPYYIDPDSLASSPGSSRAGINRIQFLLESLVDLDCGLRRLESRLLVLKGEPVQVIARLLKDWNIGKLFFEFDTEPYAQTRDNKVKDIASASGIEVFSPVSHTLFDPAEVIRKNGGKAPLTYKSFVAIAGKPSAPLRSMYSKLPPIGDIAGYEILGVPSIHDLGYKDVKQEFSPFRGGETEALKRLKENLVNKKWVAEFEKPKGDPSEFIKPATTVLSPYLKFGCLSSRYFFQCVDDAYKTVKKHTQPPVSLAGQLLWRDFFYTVAFGTPNFDRMQGNKICKQIPWRDDEKLFVAWRDAQTGYPWIDAIMIQLKKWGWMHHLARHSVACFLTRGDLFIHWEKGRDVFERLLIDSDWAINNGNWLWLSCSSFFYQYHRIYSPISFGKKYDPSGNFIRHFLPVLKDMPKEYIYEPWTAPLSIQRQANCIIGRDYPKPVVPHDLASKECKRQIGAAYALNLSGETADAEEKLNSLRRKLEEDDYDGIQNMKQKKRMSK

110.

>gi|1190968235|ref|XP_020887646.1|_(6-4)DNA_photolyase_Arabidopsis_lyrata_subsp._lyrata

MQRFSVCSPSSYRLNPITSMATRSGSLIWFRKGLRVHDNPALEFASKGSEFMFPVFVIDPHYMESDPSAFSPGSSRAGVNRIRFLLESLKDLDSSLKKLGSRLLVLKGEPGEVLFRCLQEWKVKRLCFEYDTDPYYKALDVKVKDYASSTGVEVFSPVSHTLFNPADVIEKNGGKPPLSYQSFLKIAGEPSCAKSELVMSYSSLPPVGDVGNLGISEVPSLEELGYRDDDEQADWTPFRGGESEALKRLTKSISDKAWVANFEKPKGDPSAFLKPATTVMSPYLKFGCLSSRYFYQCLQNIYKDVKKHTSPPVSLLGQLLWREFFYTTAFGTPNFDKMKGNRICKQIPWNEDHAMLAAWRDGKTGYPWIDAIMVQLLKWGWMHHLARHCVACFLTRGDLFIHWEQGRDVFERLLIDSDWAINNGNWMWLSCSSFFYQYNRIYSPISFGKKYDPDGKYIRHFLPVLKDMPKQYIYEPWTAPLSVQTKANCIVGKDYPKPMVLHDSASKECKRKMGEAYALNKKMDGKVDEENLRDLRRKLEKDEHEESKIRNQRPKLK

111.

>gi|1193802275|ref|XP_020987354.1|_(6-4)DNA_photolyase_isoform_X1_Arachis_duranensis

MLSGSGSSVMWFRKGLRIHDNPALQLASQGASHLYPLFVVDPHYMEPDPTSFSPGSSRAGLNRTKFLLESLVDLDLSLKNLGSRLLVLKGDPAEVLIRCLKEQWNVRKLCFEYDTEPYYQALDTKVKNFALGAGIEVFSPVSHTLFNPTEIIERNGGKPPLTYQSFTKIAGQPPPPLTITHSSLPPIGILGSCDISEVPTIEDLGYGDAKQDEFSPFKGGESEALKRLAECMKDKAWVAKFEKPKGNPSAFLKPATTVLSPYLKFGCLSSRYFYRQIQDVYETMPKHTSPPVSLLGQLLWRDFFYTVAFGTPNFDRMKDNKICKQIPWKDDDKLLEAWRNGRTGFPWIDAIMVQLHQWGWMHHLARHCVACFLTRGDLFVHWERGRDVFERLLIDADWAINNGNWLWLSCSSFFYQYNRIYSPTSFGKKYDPNGDYIRHFLPVLKDMPRQYIYEPWSAPLSIQTKANCIIGKDYPKPVVLHDSASKECKRKMGEAYALSKELDGVVNEDDLKNLRRKLDEGKEQETKAKRPRNTRVCMFRMLFCLLITSL

112.

>gi|1205956693|ref|XP_021316231.1|_cryptochrome-1-like_isoform_X1_Sorghum_bicolor

MSASQSSMSGAAGEPGMRTVVWFRRDLRVEDNPALAAAARTAGEVVPAYVWAPEEDGPYYPGRVSRWWLSQSLKHLDASLRRLGAGRLVTRRSNDAVVALLDLVRSTGATHLFFNHLYDPLSLVRDHRVKEQLTAEGITVQSFNADLLYEPWEVLDDDGCPFTMFAPFWNRCLCMPDPAAPLLPPKRINSGDLSRCPWDELIFEDESERGSNALLARAWSPGWQNADKALTAFLNGPLMDYSVNRKKADSASTSLLSPYLHFGELSVRKVFHQVRMKQLMWSNDGNHAGEESCTLFLRSIGLREYSRYLTFNHPCSHEKPLLSHLRFFPWVVNEVYFKVWRQGRTGYPLVDAGMRELWATGWVHDRIRVVVSSFFVKVLQLPWRWGMKYFWDTLLDADLESDALGWQYISGSLPDGRELDRIDNPQFEGYKFDPHGEYVRRWLPELARLPTEWIHHPWDAPESVLQAAGVELGSNYPRPIVELDAANSRLQDALSEMWELEAASRAAMENGMEEGLGDSTDEPLIDFPQELRMEVDRQPAQPAIHTPAVAGRRREDQMVPSMTSSFIRAETELTADFGNTSEDSRPEVPSNIHLQARAEREETVDGGTGNTVRMNGNHQQQNLQNNMHRVLGIAPSVSEASSSWTGREGGVVPVWSPPAASGHSDPYAADEADISSRSYLDRHPQSHTMMNWSQLSQSLTTGWEVDN

113.

>gi|1226790434|ref|XP_021844547.1|_deoxyribodipyrimidine_photo-lyase_Spinacia_oleracea

MTSKPVPTTTVQPERIRVLKPGSNPNGAVVYWMFRDQRVRDNWALIHAVDEANKRNAPVAVAFNLFDGFKGANARQLGFMLRGLKLLQASLHNSLHIPFFLFQGEVVETIPKFLVECGASLLVTDFTPLREIRGFKEELCKRVGDSVSIHEVDAHNVVPVWEASSKLEYGARTIRTKINKLLPTYLTDYPILQPPNCSWESSSPVIQWDQLIEDRLKKGAEVPEIDWCKPGETAALEVLKGSQNGFLTKRLKSYATDRNIPLKPGALSGLSPYLHFGQISAQRCAFEARNVRKVAPEAVDAFTEELIVRRELADNFCYYQPNYDSLMGAWEWARKTLMDHASDKREHLYTREQLEKAQTADPLWNASQLEMVHFGKMHGFMRMYWAKKILEWTSGPEEALAIAIYLNDKYEMDGRDPNGYVGCMWSICGLHDQGWRERPVFGKIRYMNYAGCKRKFNVDGYIAYVRKLVVDTKKRKAEADISSEKKKEPRC

114.

>gi|1226796804|ref|XP_021847704.1|_cryptochrome-1_Spinacia_oleracea

MGSNSKTIVWFRRDLRIEDNPALAAAARDGSVLPVFIWCPKEEAQFFPGRVSRWWMKESLAHLDNSLRSLGAELVFIRAESTLDALLECICATGATKVVFNRLYDPVSLVRDHIIKQKLGELNISVYSYNGDLLYEPWDVCDEKGNAFTTFAAFWDKCLNMQMEPSTLPTPFRLVPATGSFKKFSIGDLGLENESEKPSNALLRRAWSPGWTNANKALMEFVEQHLLDYQQSRVTVGGSSTSLLSPYLHFGELSVRKVFHSVQMKQMLWANEGNIRGCESVTMFLRAIGFREYSRYICFNFPFTHERSLLCNLKYFPWNSDQERFKAWRQGRTGYPMVDAGMRELWATGWTHNRMRVIVASFCVKVLLLHWRWGMKYFWDTLLDADLECDILGWQYISGSLPDGHDLHRLDDPELQGSKYDPEGEYIRQWLPELARLPTEWIHHPWDAPVSVLKAAGVELGSNYPKPIIEIETARDNLTEAICLMQGKAGAEETNCISEVVVDSSEKGGNTESARFNNPESFREPSIPEVVLNGKPSCITGSSRDQRVPSMQHFSNNLLQNGKRPRISVEDRAPDPNVNACYVNTEALSVQEHEADLCSTAESSSAKKQATSSFSFCVPRACSVSSKGKDSLDCESSEVKQPWKEHVDEE

115.

>gi|1226797871|ref|XP_021848229.1|_(6-4)DNA_photolyase_Spinacia_oleracea

MRFLNSLSSNHHLLLPTKLQTAKASSIVTKMTASSNSSITWFRKGLRVHDNPALEHAATGSDFVYPIFVIDPHYMDPDPDAFSPGSSRAGVNRIKFLLESLLDLDCGLRKIGSRLLVFKGEPGDVLIHCLKQLNIKKLCFEYDTDPYYQAIDARVEKYSSQEGIEVFSPVSHTLFDPRVIIHKNGGKPPLTYQSFLKLAGQPSWVSSLVSTGPSSIPPPGDVGDLQISNVPTMEELGYGNVAQHEFSPFKGGESEALKRLKLSLDDKAWVAKFEKPKGDPSSFLKPATTVLSPYLKFGCLSSRYFYQCIQEIYKNVKQHTSPPVSLHGQLLWRDFFYTAAFGTPNIDRMRGNRICKQIPWNEDSELFAAWRDGRTGYPWIDAIMIQLHKWGWMHHLARHCVACFLTRGDLFIHWEKGRDVFERLLIDSDWAINNGNWLWLSCSSFFYEYHRIYSPISFGKKHDPNGDFIRHFLPVLKDMPKQYIYEPWTAPKSIQVKAKCIIGKDYPGPVVAHDTASKECKRKLAEAYALNRLSNGSVSEDDLTKLRRKLEEDQQTPKQAVKRQKQKLITDSL

116.

>gi|1249010769|ref|XP_022533499.1|_deoxyribodipyrimidine_photo-lyase_Astyanax_mexicanus

MHCFVVAAKRTLNIFQTAAKVPRAAGLHALLDGRAASMSAKKAELKRRGGSGQAEGAGGKKQRSGESAAAAGGREDGWLGLEVAELRAKNTGCKFNDKRVRFLSEEQKVKQSCSGVLYWMSRDQRVQDNWALIYAQRLALAEELPLHICFCLVPRYLDAAYRQYAFMIRGLQEVAKECKSLDIQFHFLRGDPEQLLLDFVKSWNIGALVTDFNPLRLHLQWIENVRKGLPSNIPFLQVDAHNVVPCWEASPKLEYGARTIRGKITKQLPDFLTEFPPVDTHPHASKKTAKSVNWEEVLDSVEVDRTVGEVEWARPGTSGGMAMLESFIQQRLRLFATERNNPNSEAVSHLSPWLHAGQLSAQRVVKEVQRWGKNARESVASFTEELVVRRELADNFCYYNKEYDSIAGAYDWAKTTLKIHAKDKRAYLYTQEQLETGKTHDQLWNAAQRQLLLEGKMHGFMRMYWAKKILEWTSSPEEALTIALYLNDHYSLDGCDPNGYVGCMWSICGIHDQGWAERPVFGKVRYMNYAGCKRKFDVSRFERKYAVKTD

117.

>gi|1269931430|ref|XP_022714863.1|_(6-4)DNA_photolyase_isoform_X1_Durio_zibethinus

MKLPLSLLNSTMPSGSGSLVWFRKGLRIHDNPALEYASRGSNYVYPLFVIDPHYMEPDPNAFSPGSTRAGINRIHFLLESLHDLDLNLKKLGSRLLVLKGEPSQVLIHCLKEWDVKKLCFEYDTDPYYQALDNRVKNYASTAGIEVFSPVSHTLFNPADIIEKNGGRPPLNYQSFLKLAGEPPWASSPLSVDLSSIPPVGDVGSCEILQVPTLKELGYVEKDQDEFTPFRGGESEALRRLRESLSDKEWVANFEKPKGDPSAYVKPATTVLSPYLKFGCLSSRYFYQCLKDVYKNVKRHTSPPVSLVGQLLWREFFYTVAFGTPNFDKMKGNKICKQIPWNVDDKLLAAWREARTGYPWIDAIMVQLRKWGWMHHLARHCVACFLTRGDLFVHWEKGRDVFERLLIDSDWAINNGNWLWLSCSSFFYQYNRIYSPTSFGRKYDPNGNYIRHFLPVLKDMPKEYIYEPWTAPLSVQTKAKCIIGRDYPKPVVSHDSASKECRRKMREAYALNQKLKGLVSEEDLKKLGSKLDEDEDEGQEPNPRRKRQKLIN

118.

>gi|1280985442|ref|XP_022976147.1|_cryptochrome_DASH_chloroplastic/mitochondrial_Cucurbita_maxima

MNTLRISFSSFPLLKTLPNSSSLKPAQIAANSAHRRIFVMNSSSKLDSRSSSSSICQVPGLESEEMDRIAEQMFRRYASPSSSSVKRGKGVAIVWFRNDLRVLDNEALYKAWISSEAVLPVYCVDPRLFGSTCYFGFPKTGALRAQFIVECLADLKRNLINRGLNLLIQHGKPEEILPSLAKALGAHTVYAQMETCSEELYVERMVSKGLKTVVLSPTSEKSAKPSSAKSLTLQLVWGTTMYHIDDLPFDTNSLPDVYTQFRKSVEAKCAIRDCIRLPALLGPPASIDNWGCVPSLDKLELQPPSVVKGMRFIGGETAALSRIYEYFWKKDLLRIYKETRNGMLGPDYSTKFSPWLASGSISPRLIHEEVKRYEKEREANQSTYWVLFELIWRDYFRFLSVKYGNSLFHIGGPRKVDSKWSRDKNLFESWRDGRTGYPLIDANMKELSTTGFMSNRGRQIVCSFLVRDMGIDWRMGAEWFETCLLDYDPCSNYGNWTYGAGVGNDPREDRYFSIPKQAQTYDPEGEYVAYWLPQLRMLPKDKRHFPGKMLYMEQVVALKFGNAGRPQSQDYARRKNFGGRQAKDFRR

119.

>gi|1281042848|ref|XP_023006104.1|_cryptochrome-1_Cucurbita_maxima

MGCNKTIVWFRRDLRIEDNPALNAAARDGFVYPVYIWCPKEEGQFYPGRVSRWWLKQSLAHLKQSLKSLGSDLVLMKTQSTIFSLLECINAIGATKVAFNCLYDPISLVRDHNIKEKLVELGISVQSYNADLLYEPWDVYDENGNAFTTFKDYWGKCLLLQKEFISTLPPWKLQHAAGSVGSCSIEELGLENESEKSSNALLARAWSPGWSNADKALAEFVENHLLEYAKNRQQLGGSSTSLLSPYLHFGEVSVWKVFQKVRMKQILWAREENAVGEQSTNLFLRAIGLREYSRYICFNFPFTHERSLLSSLKFFPWHASQNNFKAWRQGRTGYPLVDAGMRELWATGWIHNRIRVIVSSFAVKVLLLPWKWGMKYFWDTLLDADLESDILGWQYISGSLPDGHELERLDDPQIQGSKYDPDGEYIRHWLPELARMPTEWIHHPWDAPQTVLKVSGVELGLNYPTPIVDLDLAANRLRESIIKMREIEAAAGANSNGTNEVVMDNADRIQSLGTANVVAEPKTCATYSSNDQKVPMIQTSKVDNPLSRKRSKPMEEKGEFQYNIRNNVQSEAGTSKPDEDLCSTAESSSSKKPSTSRTSFSVPQFCSSSKGLPESSEGTTDR

120.

>gi|1304933189|ref|XP_023095565.1|_cryptochrome-2_Felis_catus

MAAAVVTAAAAAAPAPAAGADGASSVHWFRKGLRLHDNPALLAAVRGARCVRCVYILDPWFAASSSVGINRWRFLLQSLEDLDTSLRKLNSRLFVVRGQPADVFPRLFKEWGVTRLTFEYDSEPFGKERDAAIMKMAKEAGVEVVTENSHTLYDLDRIIDLNGQKPPLTYKRFQAIISRMELPKKPVGSVTSQQMESCRAEIQENHDEAYGVPSLEELGFPTEGLGPAVWQGGETEALARLDKHLERKAWVANYERPRMNANSLLASPTGLSPYLRFGCLSCRLFYYRLWDLYKKVKRNSTPPLSLFGQLLWREFFYTAATNNPRFDRMEGNPICIQIPWDRNPEALAKWAEGKTGFPWIDAIMTQLRQEGWIHHLARHAVACFLTRGDLWVSWESGVRVFDELLLDADFSVNAGSWMWLSCSAFFQQFFHCYCPVGFGRRTDPSGDYIRRYLPMLKGFPSRYIYEPWNAPESIQKAAKCIIGVDYPRPIVNHAETSRLNIERMKQIYQQLSRYRGLCLLASVPSCVEDLSNPVAEPSSSQTGNVSSAGPRALPSGPASPKRKLEAAEEPPGEELSKRARVAGLPAPELPSRDV

121.

>gi|1333565547|ref|XP_023509705.1|_cryptochrome-2_Equus_caballus

MAAAAAVTAAAAAPAAAAAAAGAEGASSVHWFRKGLRLHDNPALLAAVRGARCVRCVYILDPWFAASSSVGINRWRFLLQSLEDLDTSLRKLNSRLFVVRGQPADVFPRLFKEWGVTRLTFEYDSEPFGKERDAAIMKMAKEAGVEVVTENSHTLYDLDRIIELNGQKPPLTYKRFQAIISRMELPRKPVGSVTSQQMESCRADIQENHDETYGVPSLEELGFPTEGLGPAVWQGGETEALARLDKHLERKAWVANYERPRMNAASLLASPTGLSPYLRFGCLSCRLFYYRLWDLYRKVKRNSTPPLSLFGQLLWREFFYTAATNNPRFDRMEGNPICIQIPWDRNPEALAKWAEAKTGFPWIDAIMTQLRQEGWIHHLARHAVACFLTRGDLWVSWESGVRVFDELLLDADFSVNAGSWMWLSCSAFFQQFFHCYCPVGFGRRTDPSGDYIRRYLPKLKAFPSRYIYEPWNAPEAVQKAAKCIIGVDYPRPIVNHAETSRLNIERMKQIYQQLSRYRGLCLLASVPSCVEDLSNPVAEPSSSQAGSVSSAGPRPPPSGPASPKRKLEAAEEPPGEELSKRARVAEPPSRAV

122.

>gi|1335140825|ref|XP_023612446.1|_cryptochrome-2_isoform_X1_Myotis_lucifugus

MAANAVTAAAAAPAPAAGTDGASSVHWFRKGLRLHDNPALLAAVRGARCVRCVYILDPWFAASSSVGINRWRFLLQSLEDLDTSLRKLNSRLFVVRGQPADVFPRLFKEWGVTRLTFEYDSEPFGKERDAAIMKMAKEAGVEVVTENSHTLYDLDRIIELNGQKPPLTYKRFQAIISRMELPKKPVASVTRHQMESCPAEIQENHDETYGVPSLEELGACFCLVGFPTEGLGPAVWQGGETEALARLDKHLERKAWVANYERPRMNANSLLASPTGLSPYLRFGCLSCRLFYYRLWDLYKKVKRNSSPPLSLFGQLLWREFFYTAATNNPRFDRMEGNPICIQIPWDRNPEALAKWAEGKTGFPWIDAIMTQLRQEGWIHHLARHAVACFLTRGDLWVSWESGFRVFDELLLDADFSVNAGSWMWLSCSAFFQQFFHCYCPVGFGRRTDPSGDYIRRYLPKLKGFPSRYIYEPWNAPESIQKAAKCIIGVDYPRPIVNHAETSRLNIERMKQIYQQLSRYRGLCLLASVPSCMEDLSNPVAEPSLSQTGSMSSAGPKPLPSGPASPKRKLEAAEEPPGEELSKRARVAELPAAELTSRDV

123.

>gi|1370461229|ref|XP_024304612.1|_cryptochrome-1_isoform_X1_Homo_sapiens

MGVNAVHWFRKGLRLHDNPALKECIQGADTIRCVYILDPWFAGSSNVGINRWRFLLQCLEDLDANLRKLNSRLFVIRGQPADVFPRLFKEWNITKLSIEYDSEPFGKERDAAIKKLATEAGVEVIVRISHTLYDLDKIIELNGGQPPLTYKRFQTLISKMEPLEIPVETITSEVIEKCTTPLSDDHDEKYGVPSLEELGFDTDGLSSAVWPGGETEALTRLERHLERKAWVANFERPRMNANSLLASPTGLSPYLRFGCLSCRLFYFKLTDLYKKVKKNSSPPLSLYGQLLWREFFYTAATNNPRFDKMEGNPICVQIPWDKNPEALAKWAEGRTGFPWIDAIMTQLRQEGWIHHLARHAVACFLTRGDLWISWEEGMKVFEELLLDADWSINAGSWMWLSCSSFFQQFFHCYCPVGFGRRTDPNGDYIRRYLPVLRGFPAKYIYDPWNAPEGIQKVAKCLIGVNYPKPMVNHAEASRLNIERMKQIYQQLSRYRGLGLLASVPSNPNGNGGFMGYSAENIPGCSSSGSCSQGSGILHYAHGDSQQTHLLKQGKNEALEHTVLFPFPILNIHFLNVQEEAPWALVSVVGNVLVRKRTHRVLVLKSRDRALIRKHSGGILLQLKLVGSSILFN

124.

>gi|1375848827|ref|XP_024452973.1|_(6-4)DNA_photolyase_Populus_trichocarpa

MQLSLCLSKPAQHLKNPLMAASGSGSIIWFRKGLRIHDNPALEYASKGSDFVYPVFVIDPHYMEPDPKAFSPGSRLAGLNRIRFLLESLVDLDTSLKKLGSRLLILRGEPGQVLTRCLKEWGVKKLCFEYDTDPHYQALDIRVKEYASAAGIEVFSPVSHTLFNPADIIQRNGGKPPLTYQSFLKLAGQPSWASSPLLTSISSLPPVGDVGSCEISEVPTIKDLGYGDIEQEWIPFRGGESEALKRLKESISDKEWVANFEKPKGNPSAFVKPATTVLSPYLKFGCLSSRYFYQCLQDVYKNVQKHTSPPVSLAGQLLWRDFFYTVAFGTPNFDRMEGNKLCKQIPWNDDDELLAAWREARTGYPWIDAIMVQLRKWGWMHHLARHSVACFLTRGDLFLHWERGRDVFERLLIDSDWAINNGNWLWLSCSSFFYQYNRIYSPISFGKKYDPNGDYIRHFLPVLKDMPKEYIYEPWTAPPGIQRKAKCIIGRDYPKPVVYHDSASKECKRKLAEAYALNKKLNGQLSQEDLDNLRRKLEQDEDQEPKIRRQRQKVGHLT

125.

>gi|1375887318|ref|XP_024466341.1|_cryptochrome-1_isoform_X1_Populus_trichocarpa

MDRSKTIVWFRRDLRIEDNPALAAAARDGCVFPVFIWCPKEEGQFYPGRVSRWWLKQSLAHLGQSLKSLGAELVLIKTHSTVAALLDCIETIGATRVVFNHLYDPVSLVRDHNIKEKLVELGISVQSYNGDLLYEPWEIYDERGHAFTTFEAYWDRCLHMQMEPVSHLPPWRLVPAAGTVMKCSVEELGLEDEAEKSSNSLLGRGWSPGWSNADKALTEFAEQHLIDYVESRLKVGTSTSLLSPYLHFGELSVRKVFQCVQLKQLLWAKEENLMGKESVTLFLRSIGLREYSRYLCFNFPFTHERSLLRNLKYFPWNDNQVHFKAWRQGRTGYPLVDAGMRELWATGWIHNKIRVIVSSFAVKVLLLPWRWGMKYFWDTLLDADLESDILGWQYISGSLPDAHELERLDNPEIQGSKFDPEGEYVRRWLPELARMPAEWIHHPWDASIAVLKAAGVELGINYPKPIIDIDLARERLMEAIFKMWEMEAAARASNTNGTNEVVVDNTDDTENLAIPKVVLKDKVTCPTNSSNDQRVPTNQNSKNIPAYRKRSKYMEEERPQPDKLHNDGNVVGTTRKDEDLCSTAESSSAKKQATSSCSFSVPQYCSSSEGKPLQESESSDLRQPLQAQIEMEQSSSKDGKQLHFIV

126.

>gi|1377713737|ref|XP_024550731.1|_Bccry2_Botrytis_cinerea_B05.10

MIRLCSTTKSILDLTTSSISRKQRHQQIATIAKMSHSNILIYLMRRDLRVGDNPVLHSLVDNKDHGFTHLLPLYVFAAQQIEVSGFITTDGCKSPYPEARSQIGAFWRCGPHRAKFLAESVWDLKGGLEKIESGLAIRVGMVDEVVKDLIEGFQKTGGSKVSAVWMTSEEGVEEKREERSTEKICDKAGVDFQLWQDEKYLIDDRDIPFKDPKDLPDVYTTYRKSVEPLREAPRPALPKPEKNSLPPFPTDVPTQHSPFAIPTNYEEIESALLKPINAQPLIKNPPSYPENSLSVHPFTGGESHAQERLEHLITSGSINAYKSSRNGLMGTDFSTKLSAYLALGSITSRQIHSSMSIFENGSDSDNRYKDLEGYGKGENEGTYGVRFELLWRDYMRLCTRKFGPKLFQLGGFKDEENAHSKWSRLDSPRDGVSKEQIQEIIERFLNGTTGMGFIDASQRECYHTGYTSNRARQNVASFLAKHLYIDWRIGAEWYECMLVDYDVSSNWGNWQYVAGVGNDPRGNDRIFNPVKQAFDYDPKAEYVLAWVDELRGVDELGQIFQAWTINDQEKKEELGIADTEMVTNPLKRIDFKINRGRGGGGGRGGGRGRPPYRPHGGDRWMGRRSGPGDQGRGGFHGGRGDRGFHQARRLYRGGRGSGSELRTGMMDKEREAAADNE

127.

>gi|1431632403|ref|XP_025610306.1|_cryptochrome-1-like_isoform_X1_Arachis_hypogaea

MSGGGGCSIVWFRRDLRVEDNPALAAGVRAGAVVAVFVYAPEEEGQYYPGRVSRWWLKNSLAHLDSSLRSLGTPLITKRSTDSVSSLLDVVKSTGATQLFFNHLYDPLSLIRDHRAKEVLTAQGITVRSFNSDLLYEPWDVNDSNGQPFTTFSAFWERCLSMPYDPQAPLLPPKRIIPGDVSRCQGDTLVFEDESEKASNALLARAWSPGWSNADKALTAFINGPLIEYAKNRRKADSATTSFLSPHLHFGEVSVKKVFHLVRIKQVLWANEGNQAGEESVNLFLKSIGLREYSRYISFNHPYSHERPLLGHLKFFPWVINESHFKAWRQGRTGYPLVDAGMRELWATGWLHDRIRVVVSSFFVKVLQLPWRWGMKYFWDTLLDADLESDALGWQYISGTLPDGREFDRIDNPQFEGYKFDPNGEYVRRWLPELSRLPTEWIHHPWNAPESVLQAAGIELGSNYPLPIVGIDAAKARLQEALIQMWQLEAASRAAIENGVEEGLGDSTELAPIAFPEDIQMEESHEPVRNNPPVGPRRYEDQMVPSMTSSLVRVEEEEASSVLRNLAEESRAEVPTNATAQQNARETVNQGVLQNVNRNTQVQHNNTTAWLRNAAEDSTAESSSSTRRERDGGVVPVWSPPSSSYAEPFVDDESGIGASSSYLQNHPQSHRLMNWTRLPQTG

128.

>gi|1432025511|ref|XP_025673554.1|_cryptochrome_DASH_chloroplastic/mitochondrial-like_Arachis_hypogaea

MAVPFFTTTFVPSLSSKTITPITPSKSTIAFIRFLTFSTMNSSPSPSSPSIYHVPDLGANDMDRVADHTFRTYTSNNNANVAKRSGKGSAIVWFRNDLRVLDNEVLYKAWLSSETVLPVFCVDPRLFSTTYHFGFPKTGALRAQFFVECLADLRKNLTKFGLNLLIQHGKPEDILPSLAKAFKAHTVYAQKETCSEELNVERLVSKGLRQVAMPSGESSSSTNSNNFPKLQLIWGTTLYHIDDLPFDAGSLPDVYTQFRKTVEAKCSIRPCIKLPVLLGPPPLVEDWGCVPSLEKLGLSSQNVSKGMKFVGGETAALSRVYEYFWKKDLLKVYKETRNGMLGPDYSTKFSPWLAFGSLSPRLIHEEVKRYEKERIANGSTYWVLFELIWRDYFRFLSVKYGNSIFYLGGPRKVQQKWSQDKILFESWRDGRTGYPLIDANMKELSTTGFMSNRGRQIVCSFLVRDMGIDWRMGAEWFETCLLDYDPCSNYGNWTYGSGVGNDPREDRYFSIPKQAQTYDPEGEYVAYWLPRLQTIPKEKRNFPGNLYIRQIVPLKYGRLQNDGARSSNDRRNDRRWNRN

129.

>gi|1432029722|ref|XP_025674864.1|_(6-4)DNA_photolyase-like_isoform_X1_Arachis_hypogaea

MLSGSGSSVMWFRKGLRIHDNPALQLASQGASHLYPLFVVDPHYMEPDPTSFSPGSSRAGLNRIKFLLESLVDLDLSLKNLGSRLLVLKGDPAEVLIRCLKEQWNVRKLCFEYDTEPYYQALDTKVKNFALGAGIEVFSPVSHTLFNPTEIIERNGGKPPLTYQSFTKIAGQPPPPLTITHSSLPPIGILGSCDISEVPTIEDLGYGDAKQDEFSPFKGGESEALKRLAECMKDKAWVATFEKPKGNPSAFLKPATTVLSPYLKFGCLSSRYFYRQIQDVYETMPKHTSPPVSLLGQLLWRDFFYTVAFGTPNFDRMKDNKICKQIPWKDDDKLLEAWRDGRTGFPWIDAIMVQLRQWGWMHHLARHCVACFLTRGDLFVHWERGRDVFERLLIDADWAINNGNWLWLSCSSFFYQYNRIYSPTSFGKKYDPNGDYIRHFLPVLKDMPRQYIYEPWSAPLSIQTKANCIIGKDYPKPVVLHDSASKECKRKMGEAYALSKELDGVVNEDDLKILRRKLDEGKEQETKAKRSRNTSGLA

130.

>gi|1448256400|ref|XP_025983854.1|_cryptochrome_1_isoform_X1_Glycine_max

MSGGGGSIVWFRRDLRIEDNPALTAGVRAGAVVAVFVWAPEEEGQYYPGRVSRWWLKNSLAHLHSSLRNLGTPLITKRSTDTLSSLLEVVKSTGATQLFFNHLYDPLSLVRDHRAKEVLTAQGITVRSFNADLLYEPWEVNDAHGRPFTTFAAFWERCLSMPYDPESPLLPPKRIIPGDASRCPSDTLLFEDELEKASNALLARAWSPGWSNANKALTTFINGPLIEYSKNRRKADSATTSLLSPHLHFGELSVKKVFHLVRIKQVLWANEGNKAGEESVNLFLKSIGLREYSRYISFNHPYSHERPLLGHLKFFPWVVNEGYFKAWRQGRTGYPLVDAGMRELWATGWLHDRIRVVVSSFFVKVLQLPWRWGMKYFWDTLLDADLESDALGWQYISGSLPDGREIDRIDNPQFEGYKFDPNGEYVRRWLPELARLPTEWIHHPWNAPESVLQAAGIELGSNYPLPIVGIDAAKTRLLEALSEMWQQEAASRAAMENGTEEGLGDSSESVPAAFPQDMQMEETHEPVRNNPLPVARRYQDQMVPSITSSLLRVEEEETSSDLRHSAEESSRAEVPVTANAQQNVGVTLNERMLQTTNRNAQTQYNTTMELRNVAEDSAVESSSGTRRERDGGVVPVWSPPASSYSEQFVGEENGITNSSSFLQRHPQSHQMLNWRQLPQTG

131.

>gi|1527515270|ref|XP_027107300.1|_cryptochrome_DASH_chloroplastic/mitochondrial-like_isoform_X1_Coffea_arabica

MVTYSSLSHPIASPFITLRKSATKVSFLLLLLKQPFPRNCKFVAAMHSNSSGTMAVSVPGVSPQEMVAIAQETFRRCTSSSSSGSSLPERRGKGVAIFWFRSDLRILDNEALIKAWLSSQALLPVYCVDPRLFTSSTHYFGFPKTGVLRAQFLMESLADLKNNLKSRGLDLLIKQGKPEDILPLLAKAHGAHTVFAQKETCSEELNVERLVAKNLRQVDQPLLKGLSTKPESKTGTKLQLIWGGSLYHIDDIPFDCKCLPDVYTQFRKSVESKSTVRACLKIPTTLGPPPNISDWGTVPEITELGFQKPKVEKGMRFVGGESAALSRLHEYFWKKDLLRIYKETRNGMLGPDYSTKFSPWLAAGNLSARFIYEEVKRYEAERQSNNSTYWVLFELIWRDYFRFLSIKEGNTLFNPGGPRKVEVNWNQDSILFDAWRDGHTGYPLIDANMRELSTTGFMSNRGRQIVCSFLVRDMGIDWRMGAEWFESCLLDYDPCSNYGNWTYGAGVGNDPREDRYFSIPKQAQNYDPEGEFVAYWLPELRALPKEKRNFPGHLYIKPVVGLKHGGSNKTSSKTRTAGRAKTWK

132.

>gi|1527533303|ref|XP_027116568.1|_cryptochrome-1_isoform_X1_Coffea_arabica

MDGKAKTIVWFRRDLRIEDNPALAAAARDGCVFPVFIWCPKEEGQFYPGRVSRWWLKQSLIQLEQSLTSLGAKLVLIKAQSTLEALLECIGAAGATKVVYNHLYDPVSLVRDHDIKQKLGELGISVQSYNGELLYEPWEVHGDDGHAFTTFDAFWDNCVHMQNEPASQLPPWRLGLCAGSVDGCSIDELGLEDESEKSSNALLGRGWSPGWSNADKALTEFVENHLCDYSKDRLRVAGNSTSLLSPHLHFGELSVRKVFHLVRMKHLLWSKEGNHDQEESANLFLRAIGLREYSRYLCFNFPFTHERSLLSTLKFFPWHADQSHFKAWRQGRTGYPLVDAGMRELWATGWIHNRIRVIVSSFFVKFLLLPWQWGMKYFWDTLLDADLESDILGWQYISGSLPDGHELERLDSPEVQGFKFDPEGEYVRHWLPELARMPAEWIHHPWDAPISVLKASGVELGLNYPKPIVDIDVARDRLIEAIFTMRGKEATARATNFNGTDEVVFDNSETSEIVGNPKAILREKLPCPTSSSHDQRVPSLQNSKNVILNRKRPMPAEDKPPLRDNVHNCNHNGETSKTDDDLRSTAESSSTKKQTTSSRTSFSVPQAVSLPLKVKPFPECESSGLKLPVEEEIDTEETSRENRAVGV

133.

>gi|1567514899|ref|XP_027834775.1|_cryptochrome-2_isoform_X1_Ovis_aries

MAAAAAATASAAAAAQAPAPRGDGASSVHWFRKGLRLHDNPALLAAVRGAHCVRCVYILDPWFAASSSVGINRWRFLLQSLEDLDRSLRKLNSRLFVVRGQPADVFPRLFKEWGVTRLTFEYDSEPFGKERDAAIMKMAKEAGVEVVTENSHTLYDLDKIIELNGQKPPLTYKRFQAIISRMELPRKPVGSVTSQQMEGCQAEIQESHDETYGVPSLEELGFPTEGLGPAVWRGGETEALARLDKHLERKAWVASYERPRMNASSLLASPTGLSPYLRFGCLSCRLFYYRLWDLYRKVKRNSTPPLSLFGQLLWREFFYTAATNNPRFDRMEGNPICIQIPWDRNPEALAKWAEGKTGFPWIDAIMAQLRQEGWIHHLARHAVACFLTRGDLWVSWESGVRVFDELLLDADFSVNAGSWMWLSCSAFFQQFFHCYCPVGFGRRTDPSGDYIRRYLPKLKGFPSRYIYEPWNAPESIQKAAKCIIGVDYPRPIVNHAEASRLNIERMKQVYQQLSRYRGLCLLASVPSCVEDLSTPVAEPSSSQAGSSSSAGPRPLPGGPASPKRKLEAAEEPPGGELSKRARVAESLPSELPSRGV

134.

>gi|49478047|ref|YP_037258.1|_deoxyribodipyrimidine_photolyase_Bacillus_thuringiensis_serovar_konkukian_str._97-27

MQNKIIVMFQKDFRLYDNPALFEAAQSGEVVPVYVHDETFSMGSASKWWLHHAIIDVKKQLEALGSTLIIRKGSTQEEILSLVEQLGITAVYWNICYDPDRLQFNQKMKMMLEHKGMICKEFNSHLLLEPWVIKKKDNTEYKVFTPFYNAFQKQVIHKPISKVQSIKGGNSLPVSLSVSELHLLPTIPWTSHMESIWEPTEEGAYKTWKEFFSSKLASYSEGRDFPNQNAHSMLAPYLSFGQISVKLIYHYLINKSTESQCSLFEKQVNSFIRQLIWREFSYYLLYHYPFTAYKPLNKSFEHFPWNNEEELLRVWQKGDTGYPFIDAGMRELWQTGFMHNRTRMAVASFLVKHLLIPWQEGAKWFMDTLLDADIANNTMGWQWVAGSGADASPYFRIFNPITQGEKFDKNGEYIREWVPELKDMPNKYIHKPWEAPEHILQKANIQLGHTYPLPVVDHKAARERALCAYKSMKEFV

135.

>gi|55978285|ref|YP_145341.1|_DNA_photolyase_(plasmid)_Thermus_thermophilus_HB8

MGPLLVWHRGDLRLHDHPALLEALARGPVVGLVVLDPNNLKTTPRRRAWFLENVRALREAYRARGGALWVLEGLPWEKVPEAARRLKAKAVYALTSHTPYGRYRDGRVREALPVPLHLLPAPHLLPPDLPRAYRVYTPFSRLYRGAAPPLPPPEALPKGPEEGEIPREDPGLPLPEPGEEAALAGLRAFLEAKLPRYAEERDRLDGEGGSRLSPYFALGVLSPRLAAWEAERRGGEGARKWVAELLWRDFSYHLLYHFPWMAERPLDPRFQAFPWQEDEALFQAWYEGKTGVPLVDAAMRELHATGFLSNRARMNAAQFAVKHLLLPWKRCEEAFRHLLLDGDRAVNLQGWQWAGGLGVDAAPYFRVFNPVLQGERHDPEGRWLKRWAPEYPSYAPKDPVVDLEEARRRYLRLARDLARG

136.

>gi|221234432|ref|YP_002516868.1|_deoxyribodipyrimidine_photolyase_Caulobacter_vibrioides_NA1000

MQVRNDSGDSKANLDAVIVWFRKDLRIADNPALRHAAQSGRPVIPLYILDETPGIRPMGGASLWWLDKSLKSLAASLETLGTKLVLRKGVAAEVLDQLIAQSGARSVVWNRLYDKPSTDRDAAIKAALRDRGVDCQSFNAGLLNEPWTVKNGSDQPYKVFTPYWRAAREHLTDVAVTAAPGHLVAPARFPASESLASWNLHPTKPDWSKGFDLWTPGEAGAHARLDAFLKGPIKGYGDQRDIPGVEATSKLSPHLHFGEIGPRQVWLATRSAADQGDIPLAEADKFLSEIGWREFNHSILYNWPHMPSANFKPEFDGFPWVKDEGALEAWKRGQTGYPIVDAGMRELWTTGFMHNRVRMIVASFLIKHLMIDWREGEAWFWDTLLDADLANNVGNWQWTAGSGADAAPYFRIFNPIAQGEKFDPKGDYVRRWVPELRNVSDDVIHKPWTKPLHLPAGAKRLYSRPIVDHAMARARALEAYHGL

137.

>gi|375135573|ref|YP_004996223.1|_deoxyribodipyrimidine_photolyase_(photoreactivation)_FAD-binding_protein_Acinetobacter_pittii_PHEA-2

MSNVNQLIWFRQDLRVRDHAALWHASQQGPCIGLIILSPEQWQTHHDAPIKINFYLRQLQQLKKELEQLNIPLIIQVIPYWKDIADYIGELSIQLNIENVYSNIEFGVNELKRDKTVQDFLNQQGKELFLFHDRTIFPLCSIRNQSQQPYQVFGAFKKACYSKLDISGLPQCYPIPEKQSSYPASFSKINSLTLEDIEAFFDPSVSKEQQGLWPAGENFALEQLDIFIKDHLSDYKLERDFPNVRGTSQLSPYLNIGILSIRQCLQALFRAEHGNFHLTNEGQQTWLDELLWREFYQHILFDFPHVSKHIPFKKNTQKIKWSHNPEHLTAWQTGQTGIPIIDAGMRQLLKTGWMHNRVRMITAMFLCKNMLIDWRVGEQWFMEHLIDGDLAANNGGWQWCASTGTDAVPYFRIFNPIAQSKKFDPNGDYIRQWVQELAHLDNKAIHEPYSTKTNIQLNYPKPIVDLKETRLKAIETFKSI
